# Expression of the aryl hydrocarbon receptor contributes to the establishment of intestinal microbial community structure in mice

**DOI:** 10.1038/srep33969

**Published:** 2016-09-23

**Authors:** Iain A. Murray, Robert G. Nichols, Limin Zhang, Andrew D. Patterson, Gary H. Perdew

**Affiliations:** 1 Center for Molecular Toxicology & Carcinogenesis Department of Veterinary and Biomedical Sciences, The Pennsylvania State University, University Park, PA-16802, USA; 2 Huck Institutes of the Life Sciences, The Pennsylvania State University, University Park, PA-16802, USA

## Abstract

Environmental and genetic factors represent key components in the establishment/maintenance of the intestinal microbiota. The aryl hydrocarbon receptor (AHR) is emerging as a pleiotropic factor, modulating pathways beyond its established role as a xenobiotic sensor. The AHR is known to regulate immune surveillance within the intestine through retention of intraepithelial lymphocytes, functional redistribution of Th17/Treg balance. Consequently, environmental/genetic manipulation of AHR activity likely influences host-microbe homeostasis. Utilizing C57BL6/J *Ahr*^−/+^ and *Ahr*^−/−^ co-housed littermates followed by 18 days of genotypic segregation, we examined the influence of AHR expression upon intestinal microbe composition/functionality and host physiology. 16S sequencing/quantitative PCR (qPCR) revealed significant changes in phyla abundance, particularly *Verrucomicrobia* together with segmented filamentous bacteria, and an increase in species diversity in *Ahr*^−/−^ mice following genotypic segregation. Metagenomics/metabolomics indicate microbial composition is associated with functional shifts in bacterial metabolism. Analysis identified *Ahr*^−/−^-dependent increases in ileal gene expression, indicating increased inflammatory tone. Transfer of *Ahr*^−/−^ microbiota to wild-type germ-free mice recapitulated the increase *Verrucomicrobia* and inflammatory tone, indicating *Ahr*^−/−^-microbial dependence. These data suggest a role for the AHR in influencing the community structure of the intestinal microbiota.

The mammalian gastrointestinal tract represents an expansive and dynamic microbial ecosystem, comprising numerous phyla and a largely uncharacterized multitude of microbial species encompassing >10^10^ individuals. The collective microbiota (and its associated microbiome) interact cooperatively and competitively as a community through both intra- (microbe-microbe) and inter-kingdom (host-microbe) communication, to profoundly influence host homeostasis and physiology. Once established, typically shortly after birth, the high-level (phyla) composition of the intestinal microbiota may remain temporally stable. Despite the apparent stability, individual microbial species and their metabolic activity are in a constant state of flux, being highly sensitive to perturbation by environmental stimuli, including fluctuation in diet^1^,^2^,^3^,^4^, xenobiotics^5^, antibiotics^6^, stress^7^ and exposure to extrinsic commensal and/or pathogenic bacteria^8^. Additionally, intrinsic genetic factors impinge upon the composition of the microbiota. Indeed, for mammals, the maternal microbiota is the primary influence upon colonization and establishment of the nascent microbiota and subsequent programming of the immune system^9^. An increasing number of genetic loci are reported to impact the microbiota. Many of these loci are associated with immune function and bring in to focus the critical relationship between the immune system, microbiota and host physiology. Recently, the aryl hydrocarbon receptor (AHR) has gained considerable attention as a modulator of immune function, especially in the context of dietary AHR activation^10^. However, its role as a genetic determinant of microbial community structure has not been fully investigated.

Historically, AHR function was restricted to regulation of cytochrome P450-mediated detoxification and as a modulator of environmental pollutant-mediated toxicity (e.g. dioxin and polychlorobiphenyls); however, with the identification of endogenous, dietary and microbial ligands, the AHR is now increasingly recognized as a pleiotropic factor^11^,^12^. An increasing volume of evidence supports a pivotal role for the AHR as a modulator of immunological development, surveillance and function within barrier tissues such as the intestine^13^. Indeed, *Ahr*^−/−^ mice are particularly susceptible to intestinal insults, such as exposure to dextran sodium sulfate. Conversely, expression of AHR in conjunction with ligand activation has been shown to be protective^14^. Of particular importance is the role of AHR and its activation by dietary or microbial-derived tryptophan derivatives such as indole-3 carbinol in facilitating gene expression associated with intestinal retention and function of group 3 innate lymphoid cells and intraepithelial lymphocytes^15^,^16^. Microbial stimulation of such cells, in combination with AHR transcriptional activity, drives the expression of IL22, which in turn stimulates epithelial cells to release anti-microbial factors including RegIII and defensins, thus contributing to epithelial barrier integrity and microbial homeostasis. Furthermore, AHR activity is a determinant in the differentiation of Th17 and Treg cells, the balance of which can profoundly influence the response to immunological stimuli at mucosal surfaces^17^.

The capacity of the AHR to direct immune functionality is therefore likely to impact host-microbe homeostasis, particularly within the intestine where microbial pressure is most intense. Here we provide evidence, using *Ahr* heterozygous and null C57BL6/J littermates, that the AHR contributes to the community structure of the microbiota and that genotypic segregation reinforces alterations in microbiota composition. Furthermore, we demonstrate that divergence of the microbiota between *Ahr* genotypes results in changes in metabolite abundance and host gene expression, which likely impacts overall host physiology.

## Results

### Genotypic segregation of *Ahr* ^−/+^ and *Ahr* ^−/−^ littermates alters the community structure of the cecal microbiota

**Figure 1:**
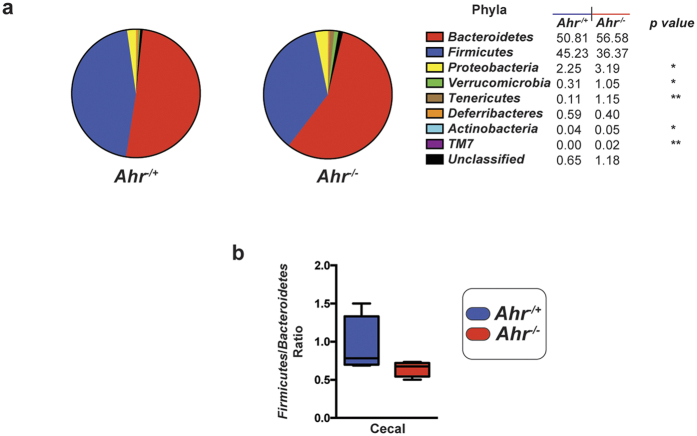
Genotypic segregation of *Ahr*^−/+^ and *Ahr*^−/−^ littermates alters the community structure of the cecal microbiota. **(a)** Pie chart representation of the phyla level community structure of the cecal microbiota from genotypically segregated *Ahr*^−/+^ and *Ahr*^−/−^ littermates as determined through 16S rDNA gene Illumina MiSeq sequencing. Data represent mean percentage abundance (*n* = 4/genotype, *p < 0.05, **p < 0.01 Student’s *t*-test). **(b)** *Firmicutes*/*Bacteroidetes* ratio based on phyla-specific 16S rDNA gene percentage abundance within cecal luminal contents.

To examine the contribution of mouse AHR expression with regard to maintenance of the community structure of the intestinal microbiota, littermates (*Ahr*^−/+^ and *Ahr*^−/−^) from congenic female C57BL6/J (*Ahr*^−/−^) and male C57BL6.129-*Ahr*^tm/Bra^/J (*Ahr*^−/+^) matings were co-housed for 6 months prior to separation based upon genotype, as illustrated in the experimental overview (Supplemental Figure 1). We adopted this approach of heterozygous and null *Ahr* littermates in favor of homozygous and null animals to facilitate equivalent microbiota exposure at birth. Additionally, heterozygous *Ahr* mice exhibit similar physiological and transcriptional responses to homozygous *Ahr* counterparts^18^. Following 18 days of genotypic isolation under identical environmental and dietary conditions within the same vivarium, bacterial 16S rDNA gene profiling was performed with DNA isolated from cecal luminal contents (Fig. 1). Phyla level abundance analysis obtained from 16S rDNA gene sequencing (mean 180,000 reads/sample, *n* = 4/genotype) between each *Ahr* genotype revealed significant differences in the percentage abundance of 16S reads associated with *Tenericutes* (10-fold), *TM7* (3-fold), *Verrucomicrobia* (3-fold), *Proteobacteria* (1.5-fold) and *Actinobacteria* (1.3-fold). No significant change in 16S reads was observed between *Ahr* genotypes with regard to *Deferibacteria* nor *Bacteroidetes* and *Firmicutes*, which together constitute the dominant (by percentage abundance) phyla in both mice and humans (Fig. 1a and Supplemental Figure 2). Although not significant, we observed opposing trends with 16S reads associated with *Bacteroidetes* and *Firmicutes* (increased and decreased, respectively) in *Ahr*^−/−^ compared to *Ahr*^−/+^ counterparts. However, this was insufficient to significantly alter the *Firmicutes*/*Bacteroidetes* ratio (Fig. 1b). To validate these 16S rDNA gene sequencing observations we performed quantitative PCR analysis upon microbial DNA isolated from the cecal contents using selected phyla-specific primers (Supplementary Figure 3a). Such analysis of phyla and total bacterial abundance confirmed the lack of significant change with regard to *Firmicutes* or *Bacteroidetes* and established no overt influence of *Ahr* genotype upon total bacterial burden within the cecum.

Quantitative PCR analysis of individual taxa or species, based on amplification of microbial DNA, is typically normalized to total bacterial abundance, in essence representing bacteria normalized to bacteria and thus lacks independent normalization. Additionally, a significant fraction of isolated microbial DNA may be derived from non-viable or metabolically inactive bacterial cells and therefore biologically or functionally irrelevant at the moment of isolation^19^. Furthermore, reports have demonstrated that 16S gene sequencing is prone to taxa-specific bias resulting in misrepresentation of abundance^20^. Therefore, to establish that these 16S rDNA gene sequencing observations accurately reflect quantification of viable, functionally relevant microbial taxa and provide an independent normalization parameter, we utilized an RNA-based quantitative PCR approach. Total RNA isolated from intact ceca (cecum and luminal microbial contents) was used as a template for quantitative reverse transcriptase PCR analysis and normalized to bacteria-independent, eukaryotic *Rpl13a* expressed by cecal tissue (Supplementary Figure 3b). Quantitative analyses of phyla abundance based on RNA isolated from intact ceca corroborated both DNA-based PCR and 16S rDNA gene sequencing data demonstrating no significant change in overall bacterial burden, *Firmicutes* or *Bacteroidetes* but confirmed that the enrichment of *Verrucomicrobia* likely represented a 3-fold increase in viable bacteria. These data indicate that AHR expression influences the high-level community structure of the microbiota within the cecal lumen albeit through adjustment of lower abundance phyla including *Verrucomicrobia*.

**Figure 2:**
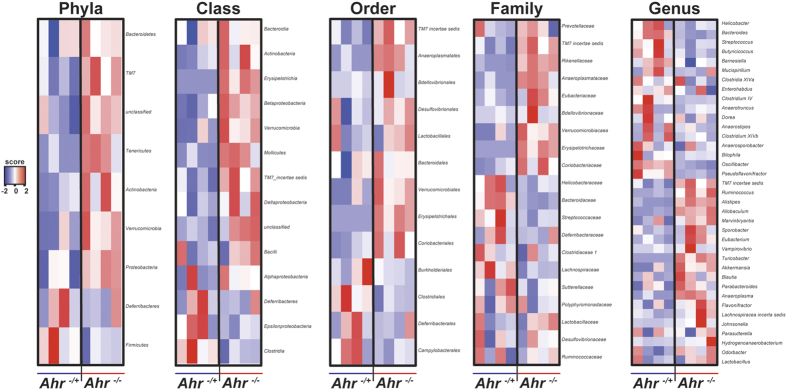
Analysis of microbial taxonomic population changes in ceca of *Ahr*^−/+^ and *Ahr*^−/−^ mice following genotypic segregation. Heat map representation of individual percentage 16S read abundance at taxonomic (phyla, class, order, family and genus) levels of cecal microbiota from genotypically segregated *Ahr*^−/+^ and *Ahr*^−/−^ littermates. Data are represented as Log_2_ transformed 16S read percentage abundance for each individual (*n* = 4/genotype) with high to low abundance indicated by red to blue, respectively.

Expanded sequence analysis identified numerous significant differences in 16S read abundance between *Ahr*^−/+^ and *Ahr*^−/−^ mice at the taxonomic class, order, family, and genus levels (Fig. 2). The trend towards depletion of 16S reads associated with *Firmicutes* in *Ahr*^−/−^ mice correlated with a reduction in the class *Clostridia*, principally unclassified genera belonging to the family *Lachnospiraceae (r* = 0.9802, *p* < 0.05). Conversely, the trend towards enrichment of 16S reads associated with *Bacteroidetes* in *Ahr*^−/−^ mice correlated with an increase in the class *Bacteroidia*, principally unclassified genera belonging to the order *Bacteroidales (r* = 0.9981, *p* < 0.01). The observed *Ahr*^−/−^ mice significant enrichment of *Proteobacteria*-associated 16S reads correlated with an increase in unclassified genera belonging to the class *Alphaproteobacteria (r* = 0.9862, *p* < 0.05). The significant enrichment of *Actinobacteria*-associated 16S reads correlated with an increase in unclassified genera belonging to the family *Coriobacteriaceae (r* = 0.9994, *p* < 0.001). The significant enrichment of 16S reads associated with *Tenericutes* in *Ahr*^−/−^ mice was attributable to an increase in the genus *Anaeroplasma*. The trend towards depletion of *Deferribacteres* observed in *Ahr*^−/−^ mice was solely attributable to a decrease in the genus *Mucisprillium*. The significant enrichment of 16S reads associated with *Verrucomicrobia* in *Ahr*^−/−^ mice appeared to be solely attributable to an increase in the genus *Akkermansia*.

To examine potential relationships between the cecal microbiota within and between each of the *Ahr* genotypes, intra-taxa level correlations were determined (Supplemental Figure 4 and Supplementary file 1). Analysis of phyla-phyla interactions within *Ahr*^−/+^ mice indicated that *Firmicutes* abundance is positively correlated with *Tenericutes* but inversely correlated with *Bacteroidetes*. Despite retaining the inverse relationship with *Bacteroidetes* in *Ahr*^−/−^ mice, *Firmicutes* failed to exhibit the correlation with *Tenericutes* within this genotype. A number of discordant class-class relationships were observed between the *Ahr* genotypes. The most significant relationship in *Ahr*^−/+^ mice was exhibited by the positive correlation between *Mollicutes* and *Alphaproteobacteria. Mollicutes* abundance in *Ahr*^−/+^ mice also correlated positively with *Clostridia*, and *Erysipelotrichia* but negatively with *Bacteroidia*. However, these relationships were not apparent in *Ahr*^−/−^ mice. At the level of order-order interactions, *Ahr*^−/+^ mice exhibited significant positive correlations between *Erysipelotrichales, Clostridales* and *Anaeroplasmatales*. A significant positive correlation was also observed between *Verrucomicrobiales* and *Bdellovibrionales* in *Ahr*^−/+^ mice. None of these relationships were evident with *Ahr*^−/−^ mice. Conversely, *Ahr*^−/−^ mice exhibited a significant negative correlation that was absent in *Ahr*^−/+^ mice between *Lactobacillales* and *Burkholderiales*. Significant *Ahr*^−/+^-associated family-family level interactions included positive correlations between *Bdellovibrionaceae* and *Streptococcaceae, Verrucomicrobiaceae; Anaeroplasmataceae* and *Eubacteriaceae, Erysipelotrichaceae*. Within *Ahr*^−/−^ animals, the positive correlation between *Bdellovibrionaceae* and *Streptococcaceae* was reversed and the positive association with *Verrucomicrobiaceae* lost and replaced with a negative correlation with *Sutterellaceae. Ahr*^−/−^ mice also exhibited a loss of association between *Anaeroplasmataceae* and *Eubacteriaceae, Erysipelotrichaceae* but acquired a positive correlation with *Prevotellaceae*. Additionally, significant positive correlations observed in *Ahr*^−/+^ animals between *Clostridiaceae* and *Prevotellaceae; Eubacteriaceae* and *Porphyromonadaceae*; and a negative association between *Rikenellaceae* and *Prevotellaceae* were not exhibited by *Ahr*^−/−^ mice. Numerous Genus-Genus level differences were evident between *Ahr* genotypes, including positive associations between *Akkermansia* and *Vampirovibrio, Ruminococcus, Allobaculum; Clostridium XVa* and *Oscillibacter* and *Odorbacter*; and *Lactobacillus* and *Bilophila, Hydrogenoanerobacterium* and *Psuedoflavonifracter* in *Ahr*^−/+^ animals that were not exhibited by *Ahr*^−/−^ mice. Combined, these data indicate differences in the steady-state cecal microbial landscape of AHR expressing and non-expressing mice, suggesting a role for AHR in maintaining the bacterial ecosystem of the intestine.

### Segregation of cecal *Akkermansia muciniphila* abundance between *Ahr* genotypes

**Figure 3:**
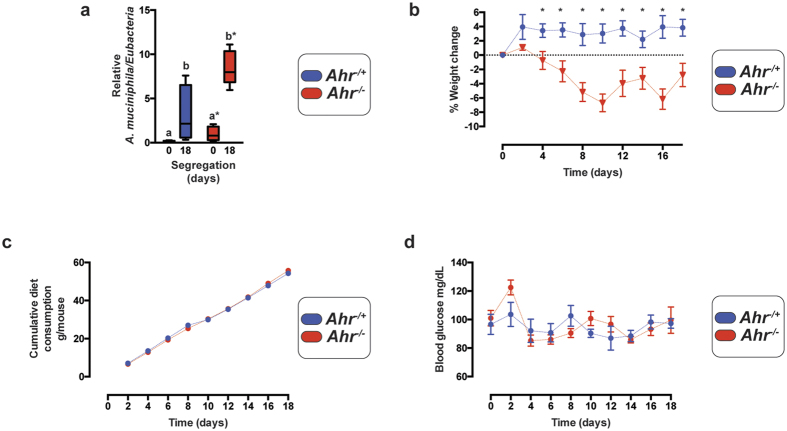
Segregation of *Akkermansia muciniphila* abundance between *Ahr* genotypes. (**a**) Quantitative real time PCR analysis of fecal *Akkermansia muciniphila* abundance prior to and following genotypic segregation. Data represent min-max and median fecal *Akkermansia muciniphila* 16S rDNA gene abundance normalized to *Eubacteria* from *Ahr*^−/+^ (blue) and *Ahr*^−/−^ (red) (n=4/genotype). Analyses were performed using Student’s *t*-test *p < 0.05. (**b**) Quantification of percentage weight change during the course of genotypic segregation. Mice were weighed every 48 h at 10–11 am and percentage weight change relative to initial weight calculated. Data represent mean percentage weight change +/− SD in *Ahr*^−/+^ (blue circle) and *Ahr*^−/−^ (red triangle) (*n* = 4/genotype). Analyses were performed using Student’s *t*-test *p < 0.05. (**c**) Quantification of food intake during genotypic segregation. Data represent cumulative food intake expressed as g/mouse (n = 4/genotype) from *Ahr*^−/+^ (blue circle) and *Ahr*^−/−^ (red circle). (**d**) Analysis of blood glucose during genotypic segregation. Blood glucose levels were determined every 48 h at 10–11 am through tail clip bleed and a hand-held glucose monitor. Data represent mean glucose concentration (mg/dL) +/− SD from *Ahr*^−/+^ (blue circle) and *Ahr*^−/−^ (red circle).

16S rDNA gene sequencing analyses identified *Verrucomicrobia* as a prominent phyla-level divergence between genotypically segregated *Ahr*^−/+^ and *Ahr*^−/−^ mice (Fig. 1). Using primers specific for *Akkermansia muciniphila (A. muciniphila*), the only currently cultured species of *Verrucomicrobia* known to colonize rodents and humans, quantitative PCR analysis performed on RNA isolated from intact ceca further established that the increase in 16S reads associated with *Verrucomicrobia* observed in *Ahr*^−/−^ animals is likely attributable to increased colonization by viable *A. muciniphila* (Supplemental Figure 3). To further examine this change in *A. muciniphila* representation further, fecal abundance was assessed from the point of genotypic segregation (Fig. 3a). Quantitative PCR performed on fecal DNA revealed an increased *A. muciniphila* load in *Ahr*^−/−^ mice when compared to *Ahr*^−/+^ animals. Following segregation, *Ahr*^−/−^ mice exhibited an increase in *A. muciniphila*, which was significantly different from than that observed in *Ahr*^−/+^ animals. An examination of body weight revealed a significant degree of weight loss by *Ahr*^−/−^ mice upon segregation, which was not evident in *Ahr*^−/+^ littermates (Fig. 3b). Examination of food intake revealed a mean intake of ~3 g/mouse/day by both genotypes thus indicating that the weight loss exhibited by *Ahr*^−/−^ animals could not be due to diminished appetite (Fig. 3c). Analysis of blood glucose levels did not reveal any significant difference between the *Ahr* genotypes over the time course of segregation, suggesting that the observed increase in *A. muciniphila* does not impact serum glucose levels (Fig. 3d).

### Cecal microbiota from *Ahr* ^−/+^ and *Ahr* ^−/−^ mice exhibit divergent metagenomic metabolic pathway profiles

**Figure 4:**
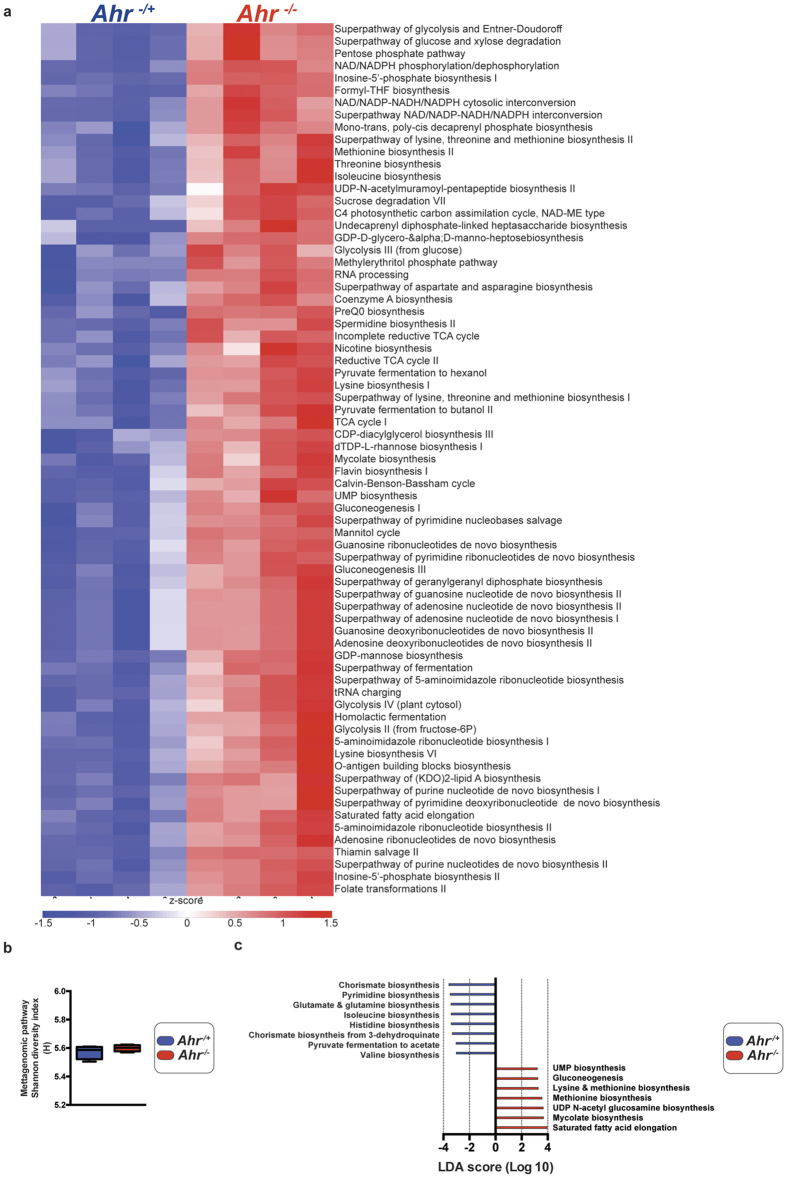
HUMAnN2 metagenomic metabolic pathway analysis. (**a**) Metagenomic pathways exhibiting significant changes in representation. Microbial DNA from cecal contents was sequenced and pathway coverage determined using the HUMAnN2 pipeline. The pathway coverage output representing the number of gene families associated with each pathway were processed to include only those pathways with >50% coverage. Data represent those pathways exhibiting a difference in abundance below a significance threshold of *p* < 0.001 (Student’s *t*-test) expressed as a *z*-score. (**b**) Shannon diversity index associated with metagenomic pathways. Diversity indices were calculated and presented as min-max and median H values (n = 4/genotype). (**c**) Linear discriminant analysis (LDA) of metagenomic pathways. Pathway abundance data were applied to the LDA algorithm with a α = 0.05 threshold for Kruskal-Wallis and pairwise Wilcoxon tests combined with a 2.0 logarithmic LDA cut-off to identify pathway components which most significantly discriminate between *Ahr* genotypes. Data represent Log_10_ LDA score for indicated pathways within *Ahr*^−/+^ (blue) and *Ahr*^−/−^ (red) mice.

Having established that genotypically segregated *Ahr*^−/+^ and *Ahr*^−/−^ littermates harbor divergent cecal microbial populations without an overall change in total bacterial burden, we utilized whole genome metagenomic pathway analysis, adopting the HMP Unified Metabolic Analysis Network 2 (HUMAnN2) pipeline, to assess the potential differences in metabolic pathway representation within the differing microbial community structures. HUMAnN2 analyses allows for identification and assessment of those pathways that are represented with greater than 50% pathway coverage. Such analyses were performed and identified 1144 MetaCyc pathways or superpathways that were represented in either or both *Ahr* genotypes. Of these represented pathways, 468 (~40%) exhibited a significant difference in abundance between *Ahr*^−/+^ and *Ahr*^−/−^ animals, indicating the potential for metabolic consequences within the host (Fig. 4a). The 70 most prominent differences were observed with pathways associated with amino acid biosynthesis and degradation; purine/pyrimidine biosynthesis/salvage; folate transformations; glycolysis and anaerobic respiration, all of which exhibited significantly enhanced representation in cecal microbiota from *Ahr*^−/−^ mice. Despite these changes in pathway abundance, analysis of the Shannon diversity index associated with the cohort of metagenomic pathways in each genotype failed to identify a significant separation (Fig. 4b). Linear discriminate analysis (LDA) was performed to identify those pathways that discriminate between *Ahr* genotypes (Fig. 4c). In Particular, LDA analysis identified UMP, lysine and methionine, UDP N-acetyl glucosamine, mycolate biosynthetic pathways, gluconeogenesis and fatty acid elongation processes as the most discriminatory factors within the metagenome of *Ahr*^−/−^ associated cecal microbiota. Whereas the *Ahr*^−/+^ cecal metagenome was most associated with pyrimidine salvage, chorismate, glutamate and glutamine, isoleucine, histidine, valine and chorismate from 3-dehydroquinate biosynthetic pathways, as well as pyruvate to acetate processes. These results support 16S rDNA gene sequencing data demonstrating a functionally altered community structure.

### Alteration of microbial community structure is associated with changes in cecal metabolite signatures in genotypically segregated *Ahr* ^−/+^ and *Ahr* ^−/−^ littermates

**Figure 5:**
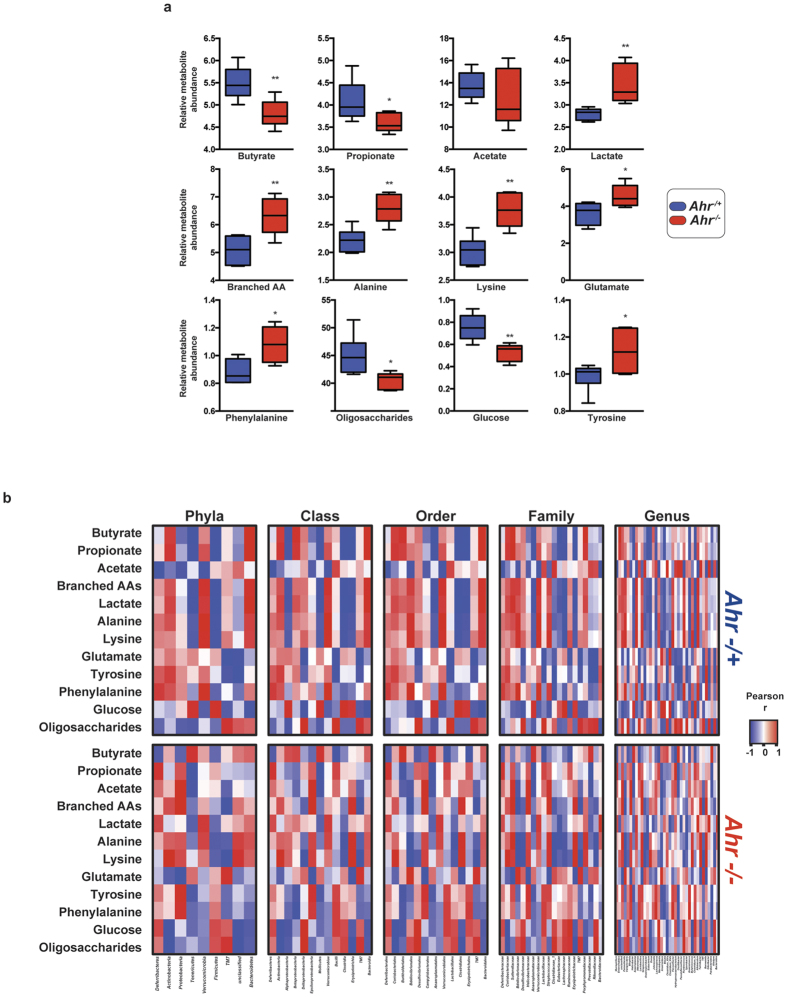
Genotypically segregated *Ahr* mice exhibit differential cecal metabolite profiles. (**a**) Cecal contents from genotypically segregated *Ahr*^−/−^ and *Ahr*^−/+^ mice. Data represent relative min-max and median peak integration for the indicated metabolite. (**b**) Heat map representations of correlative taxa-level interactions between cecal microbiota and metabolites from genotypically segregated *Ahr*^−/+^ and *Ahr*^−/−^ littermates. Data represent Pearson correlation (*r*) coefficients with positive to negative correlation indicated by red to blue, respectively. Correlation coefficients and *p* values are detailed in Supplementary file 2.

Our observation that genotypic segregation of *Ahr*^−/+^ and *Ahr*^−/−^ littermates facilitates changes in the representation of selected microbial metabolic pathways prompted an examination of the metabolite profiles associated with the cecal luminal contents (Fig. 5a). Relative quantification through ^1^H-NMR peak integration analyses identified significant decreases in the short-chain fatty acids (SCFAs) butyrate and propionate within the luminal contents of *Ahr*^−/−^ mice, indicative of reduced microbial fermentation. *Ahr*^−/−^ mice exhibited a significant increase in lactate that inversely correlated with glucose levels, suggesting an elevated glycolytic flux, which is supported by metagenomic pathway analyses indicating greater representation of the microbial glycolytic pathway in *Ahr*^−/−^ animals. Consistent with our pathway analyses demonstrating enrichment of various amino acid biosynthetic pathways in microbiota from *Ahr*^−/−^ mice, we observed significant increases in the relative abundance of branched-chain amino acids (valine, leucine and isoleucine), alanine, lysine, glutamate, tyrosine and phenylalanine within cecal luminal contents.

To examine potential associations between the observed changes in cecal metabolites and the altered microbial landscape in *Ahr*^−/−^ mice, Pearson correlation coefficients were determined (Fig. 5b and Supplemental file 2). The reduction in the SCFA butyrate failed to associate at the phylum or class level but exhibited a significant positive correlation with the order *Burkholderiales (r* = 0.9794, *p* < 0.05) and the genera *Oscillibacter* and *Parabacteroides (r* = 0.998, *p* < 0.01). In contrast, cecal butyrate levels in *Ahr*^−/+^ mice exhibited positive correlations with the phylum *Bacteroidetes (r* = 0.9595, *p* < 0.05) and the *Sutterellaceae (r* = 0.9946, *p* < 0.01) family, combined with negative associations at family and genus levels with *Erysipelotrichiaceae (r* = −0.9748, *p* < 0.05) and *Eubacterium (r* = −0.9596, *p* < 0.05) respectively. Within *Ahr*^−/−^ animals, the reduction in the SCFA propionate negatively correlated with the *Mollicutes* class (*r* = −0.9665, *p* < 0.05). Within *Ahr*^−/+^ mice, propionate abundance negatively correlated with *Anaerotruncus (r* = −0.9183, *p* < 0.05), members of *Firmicutes*. Although no significant difference in the SCFA acetate was observed between *Ahr* genotypes, its abundance exhibited divergent microbiota associations, being negatively correlated with *Muscispirillum (r* = −0.969, *p* < 0.05) and positively with *Oscillibacter (r* = 0.9579, *p* < 0.05) in *Ahr*^−/+^ mice but not *Ahr*^−/−^ animals. The significant increase in cecal lactate observed in *Ahr*^−/−^ mice negatively associated with *Clostridium XVb (r* = −0.9684, *p* < 0.05) and positively with *Allobaculum (r* = 0.9988, *p* < 0.05) but negatively with *Clostridales (r* = −0.9543, *p* < 0.05) in *Ahr*^−/+^ animals. Cecal glucose significantly positively correlated with *Ruminococcaceae (r* = 0.9637, *p* < 0.05) in *Ahr*^−/−^ mice but with *Lachnospiraceae (r* = 0.9683, *p* < 0.05) in *Ahr*^−/+^ animals. Cecal oligosaccharide content exhibited a significant negative correlation with *Coriobacteriaceae (r* = −0.9870, *p* < 0.05) in *Ahr*^−/−^ mice. In contrast, within *Ahr*^−/+^ animals oligosaccharide levels were positively associated with *Lactobacilliaceae (r* = 0.9757, *p* < 0.05). These data provide supporting evidence that the altered microbiota together with concomitant changes in metabolic pathway representation observed within *Ahr*^−/−^ mice leads to quantifiable metabolite differences.

### Segregation of ileal segmented filamentous bacteria abundance between *Ahr* genotypes

**Figure 6:**
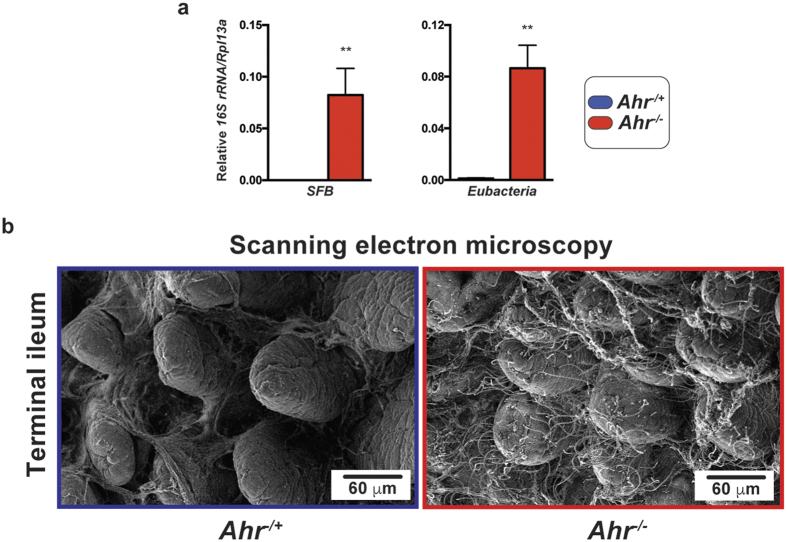
Segregation of ileal Segmented filamentous bacteria abundance between *Ahr* genotypes. (**a**) Real time PCR quantification of ileal SFB abundance together with total bacterial load normalized to eukaryotic *Rpl13a* using specific primers upon cDNA generated from total RNA isolated from intact ileal tissue (ileum and luminal contents) from *Ahr*^−/+^ (blue) and *Ahr*^−/−^ (red) mice. Data represent mean (*n* = 4/genotype) 16S rRNA gene abundance −/+ SD. Analyses were performed using Student’s *t*-test *p < 0.05, **p < 0.01. (**b**) Scanning electron microscopy of terminal ileum excised from *Ahr*^−/+^ and *Ahr*^−/−^ mice demonstrating increased SFB colonization. Scale bar indicates 60 μm. Data are representative of images from multiple mice of each *Ahr* genotype.

Previous studies have highlighted the significance of ileum-specific segmented filamentous bacteria (SFB) (*Candidatus savagella* or *arthromitus*) in determining host-microbe responses^21^. We therefore examined the degree of ileal colonization of this *Clostrida* species within genotypically segregated *Ahr* mice. Quantitative PCR analysis of terminal ileum RNA revealed a marked and significant increase in viable SFB abundance in *Ahr*^−/−^ mice when compared to their heterozygous littermates (Fig. 6a). PCR quantification of total bacterial burden within the ileum of these mice also indicated a significantly increased bacterial load. The similar magnitude of the increases associated with SFB and total bacteria suggested that the elevated bacterial burden within the terminal ileum of *Ahr*^−/−^ mice might be largely attributable to SFB expansion. To further examine the *Ahr*^−/−^-associated increase in SFB, we visually examined the terminal ilea of these mice through scanning electron microscopy (Fig. 6b). Visualization of SFB revealed marginal to non-existent colonization in *Ahr*^−/+^ animals but identified large numbers of *Ahr*^−/−^ epithelia-associated SFB, confirming the quantitative expansion observed in these mice. The established influence of SFB in determining host immunological responses to the microbiota and other antigenic sources suggests that the distinct difference in colonization is likely to influence the physiology of these mice^16^.

### Genotypically segregated *Ahr* ^−/−^ mice display an enhanced inflammatory tone within the intestine

**Figure 7:**
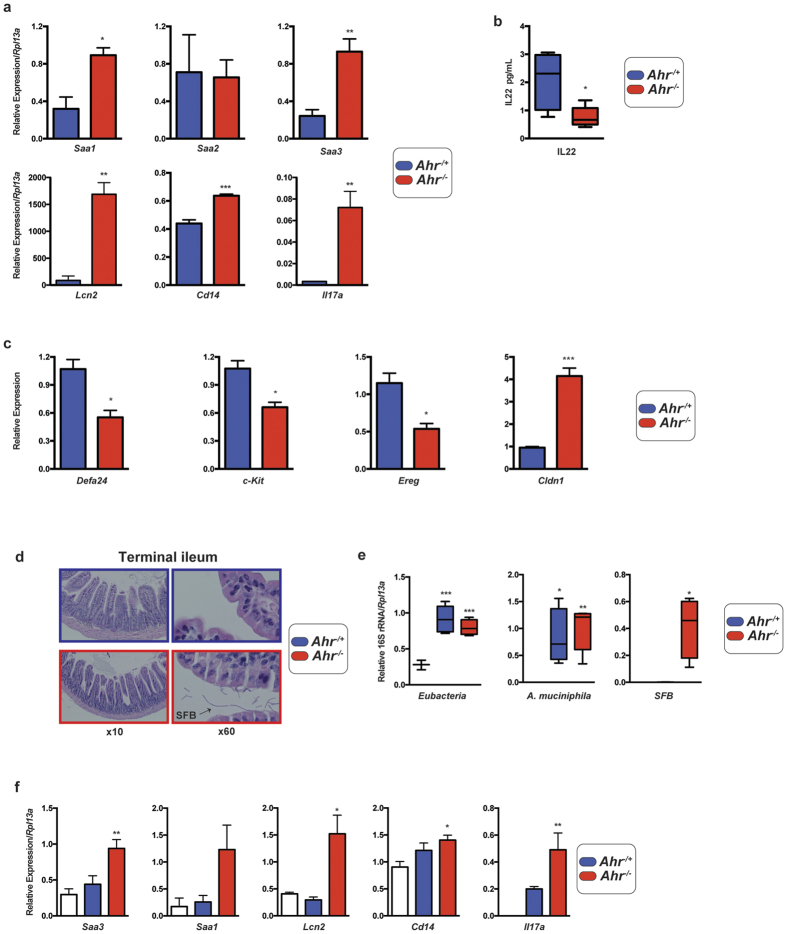
Genotypically segregated *Ahr* mice exhibit differential ileal gene expression profiles. (**a**) Real time PCR quantification of ileal gene expression normalized to eukaryotic *Rpl13a* using specific primers upon cDNA generated from total RNA isolated from intact ileal tissue (ileum and luminal contents) from *Ahr*^−/+^ (blue) and *Ahr*^−/−^ (red) mice. Data represent mean (*n* = 4/genotype) relative gene expression −/+ SD. Analyses were performed using Student’s *t*-test *p < 0.05, **p < 0.01, ***p < 0.001. (**b**) ELISA quantification of ileal IL22 from *Ahr*^−/+^ (blue) and *Ahr*^−/−^ (red) mice. Data represent min-max and median IL22 (*n* = 4/genotype) level from *Ahr*^−/+^ (blue) and *Ahr*^−/−^ (red) mice expressed as pg/mL. Analyses were performed using Student’s *t*-test *p < 0.05. (**c**) NanoString™ quantification of ileal gene expression. Total RNA isolated from ilea was processed through the NanoString nCounter system. Data were normalized to positive control probes (assay and house-keeping genes) and represent mean relative expression level +/− SD. Analyses were performed using Student’s *t*-test *p < 0.05, **p < 0.01, ***p < 0.001. (**d**) Histological sections of terminal ilea of *Ahr*^−/−^ and *Ahr*^−/+^ mice. Terminal ilea were excised, formalin fixed, sectioned (5 μm) and stained with hematoxylin & eosin. Data are representative of 10× and 60× magnification images of multiple sections from each genotype (*n* = 4/genotype). (**e**) Cecal microbiota profile from genotypically segregated *Ahr* mice is transmissible to wild type germ free animals. Pooled cecal contents isolated from genotypically segregated mice (*n* = 4/genotype) were used to orally inoculate wild type germ free animals. Cecal abundance of *Eubacteria, A. muciniphila* and *SFB* were determined by qPCR performed on total RNA following 5 days of germ free animals. Data represent min-max and median abundance of indicated species following normalization to eukaryotic *Rpl13a* in the ceca of formerly germ free animals (*n* = 4/treatment; *p < 0.05, **p < 0.01 and ***p < 0.001). (**f**) Ileal gene expression in wild type germ free animals inoculated with cecal contents from genotypically segregated *Ahr* mice. Ileal expression of indicated targets were determined by qPCR on RNA isolated following 5 days of colonization. Data represent mean expression normalized to *Rpl13a (n* = 4/treatment; *p < 0.05, **p < 0.01).

Our experimental approach using *Ahr*^−/+^ and *Ahr*^−/−^ littermates cohoused for 6 months allowed for initial exposure to the same maternal microbiota, yet analysis identifies microbial divergence following subsequent genotypic segregation, thus implicating host influences. The ileum is regarded as a major site of host-microbiota interaction, surveillance and education through modulation of host immunity. We therefore examined gene expression within the terminal ilea of genotypically segregated *Ahr* mice to determine if it may account for the observed microbial divergence. Quantitative PCR and NanoString™ expression analysis were performed upon ileal samples and identified significant differences in gene targets previously reported to be influenced by or modulate the microbiota (Fig. 7). Ileal expression of the acute phase reactants *Saa1, 2 & 3*, which are induced in response to commensal bacteria, particularly segmented filamentous bacteria (SFB), displayed *Ahr* genotype-dependent expression with significantly elevated levels of *Saa1* and *Saa3* but not *Saa2* in *Ahr*^−/−^ mice (Fig. 7a). Associated with the increase in *Saa1/3*, we also observed a corresponding significant increase in *Il17a* expression, which is consistent with the expansion of SFB and Th17 development in these mice^21^. However, ELISA-based quantification of ileal IL22, a contributor to microbiota-dependent epithelial *Saa* induction, exhibited decreased abundance in *Ahr*^−/−^ animals (Fig. 7b). These mice also displayed enhanced expression of the siderophore *Lcn2* and the LPS and pathogen-associated molecular pattern co-receptor *Cd14*. In conjunction with this increase in pro-inflammatory markers, NanoString™ quantification revealed a significant reduction in ileal expression of the anti-microbial defensin *Def24a*. Significantly reduced ileal expression of *c-kit (Cd117*) and *Ereg* were also observed in *Ahr*^−/−^ mice (Fig. 7c). Conversely, a significant increase in tight junction-associated *Cldn1* was evident in *Ahr*^*−/−*^ animals. Additionally, *Ahr*^−/−^ mice exhibited a trend towards enhanced expression of the cytokines *Il1b, Il6, Cxcl5* and *Il10*, each suggestive of an inflammatory response, although these increases failed to achieve statistical significance (data not shown). Despite the observed changes in gene expression suggestive of heightened inflammatory signaling and diminished anti-microbial expression, histological examination of ileal tissue failed to identify any overt evidence of gross inflammation in *Ahr*^−/−^ animals whilst maintained under a specific pathogen-free environment (Fig. 7d).

### Inflammatory gene expression associated with the microbiota of *Ahr* ^−/−^ mice is transmissible to germ-free animals

To assess whether the microbiota associated with *Ahr*^−/−^ animals is a causative agent behind the enhanced inflammatory gene expression observed in these mice, we examined the effect of cecal microbiota transfer from genotypically segregated *Ahr* mice into germ free wild type animals (Fig. 7e). Germ free wild type mice were administered with a single oral inoculum of pooled cecal contents from *Ahr*^−/−^ or *Ahr*^−/+^ animals and maintained under a germ free environment for 5 days prior to analysis of cecal bacterial abundance and host ileal gene expression. This colonization timeframe was adopted to maintain the original inoculate community structure and limit microbial drift arising from the *Ahr*^+/+^ status of the germ free recipients. Quantitative PCR analysis of bacterial abundance performed on RNA isolated from ceca confirmed microbial colonization had occurred in formerly germ free animals. Inoculation from *Ahr*^−/−^ or *Ahr*^−/+^ mice resulted in similar levels of *eubacteria* colonization (Fig. 7e). Consistent with the increased abundance in donor animals, germ free animals inoculated with cecal contents from *Ahr*^−/−^ mice exhibited a greater degree of colonization by *A. muciniphila* than that obtained with *Ahr*^−/+^ animals. Similarly, SFB colonization of germ free animals was established to a greater degree following inoculation with *Ahr*^−/−^ animal-derived cecal contents. Analysis of host gene expression identified significantly increased expression of *Saa3, Lcn2, Cd14* and *Il17a* within the ilea of germ free animals exposed to *Ahr*^−/−^-derived cecal microbiota when compared to those inoculated with cecal contents from *Ahr*^−/+^ mice and control non-inoculated germ free animals (Fig. 7f).

## Discussion

A number of genetic and toxicological studies demonstrate the involvement of the AHR and its ligands in the establishment, maintenance and regulation of immune function^5^,^10^,^15^,^22^. Given the intimate association between host immunity and the microbiota we have therefore examined the impact of genetic AHR ablation upon the composition of the intestinal microbiota. Using a mouse model of co-housed *Ahr*^−/+^ and *Ahr*^−/−^ littermates, subsequently segregated based upon genotype we demonstrate significant differences with regard to microbial community structure between these genotypes. This demonstrates that despite the opportunity for acquisition of a common microbiota within littermates the AHR genotype contributed to a selective pressure on the microbiota of the offspring. Despite the importance of AHR in facilitating the development and function of many immunological cell types *e.g.* Th17, Treg, B cells and mast cells, microbial overgrowth was not evident within the ceca of *Ahr*^−/−^ mice. The dominant (by percentage abundance) phyla, *Firmicutes* and *Bacteroidetes*, were not overtly influenced by genetic AHR ablation; consequently, no change in *Firmicutes*/*Bacteroidetes* ratio was observed indicating that a major phyla level shift in community structure had not occurred. However, numerous significant differences between *Ahr* genotypes were observed at sub-phyla taxonomic levels, indicating that *Ahr*^−/−^ mice harbor similar microbiota that undergoes a significant divergence upon segregation. Furthermore, metagenomic and metabolomic analyses indicate that redistribution of the microbiota in *Ahr*^−/−^ animals was associated with significant changes in the cecal microbiome and metabolites and is thus likely to contribute to host physiology.

The most prominent microbial differences witnessed between the *Ahr* genotypes were marked *Ahr*^−/−^-associated expansions of *Akkermansia muciniphila (A. muciniphila*) and segmented filamentous bacteria (SFB) within the cecum and ileum respectively. Interestingly, both species are widely studied and reported to exert profound effects upon rodent and human physiology arising from their proximity to the intestinal epithelium^21^,^23^. In humans and rodents, *A. muciniphila* typically accounts for <1–5% of the total microbiota^23^,^24^. Occupying a niche within the luminal surface of the mucus layer, *A. muciniphila* degrades host mucin oligosaccharides liberating nutrients for other microbes to utilize^25^,^26^. Importantly, increased *A. muciniphila* abundance is suggested to confer a number of beneficial effects with regard to maintenance of epithelial barrier integrity, and protection from inflammatory intestinal conditions, colorectal cancer, insulin resistance, dyslipidemia and obesity, although the mechanisms have not yet been established^27^,^28^,^29^,^30^. Our observation of *A. muciniphila* enrichment in *Ahr*^−/−^ mice is intriguing and suggests that these mice may exhibit some of the health benefits associated with *A. muciniphila* expansion. Indeed, *Ahr*^−/−^ mice are reported to be resistant to diet-induced obesity and protected from insulin insensitivity, although this was not examined in the context of the microbiota^31^. Our data, which demonstrates a significant weight loss without a concomitant reduction in food intake, which tracked with increased fecal *A. muciniphila* abundance in *Ahr*^−/−^ animals, is supportive for a role of *A. muciniphila* in reducing adiposity. However, the association of weight loss and *A. muciniphila* in segregated *Ahr*^−/−^ mice could not be attributed to SCFAs, which are reported to exhibit anti-adipogenic activity^32^. Indeed, with the exception of phenylalanine in *Ahr*^−/+^ mice, we failed to identify any correlation between *A. muciniphila* and cecal metabolites. We did not observe any overt inflammation within *Ahr*^−/−^ mice under pathogen-free conditions which is consistent with other reports^15^. AHR activity has previously been shown to modulate local and systemic expression of inflammatory mediators, cytokines and acute phase reactants^33^,^34^,^35^. In addition, previous studies have demonstrated that ablation of AHR expression renders increased sensitivity to a range of intestinal insults including models of inflammatory bowel disease and pathogenic infection although these observations however, were not specifically examined in the context of the microbiota^14^,^36^,^37^. The elevation of inflammation-associated markers, such as *Saa, Lcn2* and *Cd14* within the ileum of *Ahr*^−/−^ animals suggests that these mice may be subject to low-grade inflammatory stress^38^,^39^.

The etiology of enhanced inflammatory tone and the change in the microbial landscape exhibited by *Ahr*^−/−^ mice is likely to be multi-factorial and interrelated. SCFA levels, which are diminished in *Ahr*^−/−^ animals as a likely consequence of a decrease in *Clostridia* species, has previously been shown to enhance mucosal barrier function, thus bacterial translocation may be more permissive in these mice and would be consistent with the observed increase in expression of ileal *Lcn2* and *Cd14*. Additionally, loss of AHR expression is demonstrated to disrupt the immunological milieu of the intestine allowing for decreased microbial surveillance that may in part account for the expansion of *A. muciniphila*. Reduced colonization of intra-epithelial lymphocytes reported in *Ahr*^−/−^ mice is likely to exert a profound effect upon inflammatory signaling and the microbial ecosystem^15^. Indeed, we observed significantly decreased IL22 levels that were associated with decreased expression of the anti-microbial *Def24a*. It is conceivable that reduced IL22-mediated defensin production may have contributed to the preferential expansion of *A. muciniphila* due to their proximity to the normally anti-microbial peptide rich mucus. Similarly, T and B cell-deficient *Rag1*^−/−^ mice also exhibit expansion of *A. muciniphila*. Additionally, the ilea of *Ahr*^−/−^ mice demonstrated a robust expansion of SFB, which penetrate the mucus layer to physically attach to the epithelial layer^40^. Therefore reduced anti-microbial peptide activity is likely to facilitate SFB expansion. Importantly, these studies were performed using co-housed *Ahr*^−/+^ and *Ahr*^−/−^ littermates, indicating *Ahr*^−/−^ animals are acutely receptive to SFB colonization and expansion when compared to *Ahr*^−/+^ littermates, despite an equivalent initial colonization environment. Consistent with previous studies, SFB expansion in *Ahr*^−/−^ mice was accompanied by significant increase in *Il17a* expression within the ileum despite studies indicating the apparent requirement for AHR signaling to generate a Th17 polarizing environment^15^,^16^. A heightened IL17A response is likely to contribute to the manifestation of inflammation and increased sensitivity to insults observed within the *Ahr*^−/−^ mice.

Transfer of *Ahr*^−/−^ animal-derived microbiota into formerly germ free wild type animals and a subsequent increase in inflammation-associated gene expression within these animals suggests that a microbial component is causative of the change in inflammatory tone in both *Ahr*^−/−^ and formerly germ free wild type animals. Whether colonization of germ free animals with *Ahr*^−/−^-derived microbiota directly facilitated the increase in inflammatory markers or inoculation with microbial metabolites, such as LPS stimulated expression is unclear. However, considering gene expression was assessed after five days argues against such metabolite-mediated changes. Furthermore, the enhanced expression however of genes known to be sensitive to SFB colonization, e.g. *Saa1/3* and *Il17a*, suggests a direct consequence of microbial interaction with the epithelium and education of intestinal immune cells.

The data presented herein suggest that absence of AHR expression has a marked effect upon the microbiota, particularly those that are in close proximity to the epithelium, and provides further evidence that host genotype exerts a significant influence. It is unclear whether colonization or altered abundance of species, such as *A. muciniphila* or SFB, is causative or a marker of the overall redistribution and change in diversity of the microbiota in *Ahr*^−/−^. However, the influence of SFB in determining the immunological status of the host, together with the capacity of *A. muciniphila* to provide nutrients for the benefit of other species, which in turn cooperate and compete in a complex network, may point to important roles for these bacteria in shaping the intestinal microbiota and host physiology.

The use of global *Ahr*^−/−^ animals in this study did not allow the relative contribution of AHR expression within different intestinal cell types to be addressed. However, studies utilizing conditional intestinal epithelia-specific *Ahr*^−/−^ mice may highlight roles for AHR in non-immune cells in determining the intestinal microbial ecosystem. Furthermore, this and future studies may provide a framework to assess the influence of dietary AHR ligands upon the composition of the intestinal microbiota, microbiome and host physiology. Importantly, the observed differences in microbiota composition demonstrated here and the growing evidence supporting the importance of the microbiota in shaping host physiology suggest that studies utilizing *Ahr* deficient mice and/or AHR ligands may require assessment of the influence of the microbiota for a comprehensive interpretation of results.

## Methods

### Animals and husbandry

All animal studies were performed with approval and under the auspices of the Institutional Animal Care and Use Committee (IACUC, The Pennsylvania State University). All animal experiments were carried out in accordance to IACUC guidelines. C57BL6/J mice were originally purchased from Jackson Laboratories (Bar Harbor, ME.) and subsequently bred in-house. *Ahr* null mice (B6.129-*Ahr*^*tm1Bra*^/J) were obtained as a gift (Dr. Christopher Bradfield, University of Wisconsin, Madison, WI.) and subsequently bred in-house. Animals were housed in autoclaved polypropylene cages with corncob bedding under a specific pathogen-free environment within the same vivarium under a standard 12 h light/dark cycle (light 0600–1800 h eastern standard time) with *ad libitum* access to autoclaved standard animal chow and water.

### *Ahr* genotyping

Female *Ahr*^−/+^ and *Ahr*^−/−^ littermates were genotyped using genomic DNA isolated from tail clips as previously described (www.Jax.org B6;129-*Ahr*^tmBra^/J strain # 0027272).

### Bacterial DNA isolation

Bacterial genomic DNA was isolated from 100 mg cecal contents and fecal pellets using Powersoil DNA isolation kit (Mobio, Carlsbad, CA.) according to the manufacturer instructions. For metagenomic studies, cecal bacterial DNA was extracted using EZNA stool DNA kit (Omega Bio-tek, Norcross, GA) following the manufacturer recommended instructions. Following extraction using either method, DNA concentration was normalized to 20 ng/µl and stored at −80 °C.

### 16S rDNA gene Illumina MiSeq sequencing

Bacterial DNA extracted using a Powersoil DNA isolation kit and 20 ng template DNA were amplified across the V4/V4 region of the 16S rDNA gene utilizing the primers (515F: TCGTCGGCAGCGTCAGATGTGTATAAGAGACAGGTGCCAGCMGCCGCGGTAA and 806R: GTCTCGTGGGCTCGGAGATGTGTATAAGAGACAGGGACTACHVGGGTWTCTAAT) in combination with FastStart high-fidelity amplification kit (Roche, Indianapolis, IN.) and the following cycling conditions (94 °C, 3 min; 94 °C, 15 sec, 55 °C, 45 sec; 72 °C, 1 min for 30 cycles; 72 °C, 8 min). Verification of product amplification was demonstrated through agarose gel electrophoresis and the visualization of a single product of 359 bp. Amplified V4 16S rDNA gene products were supplied to the Genomics Core Facility (The Pennsylvania State University) for 16S sequencing using the Illumina MiSeq platform. De-multiplexed sequencing reads were processed through the Mothur software package^41^. Reads were trimmed at 320 bp and subsequently aligned to the Silva reference genome and chimaeras removed with the Uchime software package^42^. Reads were classified using the Ribosome Database Project classifier set to 75% and used to compile a phylogenetic tree on Mothur with clearcut. Classified reads were normalized to total reads to yield relative taxonomic abundance.

### Whole genome pathway metagenomic analysis

Microbial DNA was isolated from cecal contents using ENZA stool DNA kit. Whole genome sequencing was performed using the Illumina HiSeq 2500 platform within the Pennsylvania State University Genomics core facility. Raw sequencing reads, averaging 18 × 10^6 ^reads/sample, were processed through the HUMAnN2 v0.99 (http://huttenhower.sph.harvard.edu/humaan2/manual#markdown-header-workflows)^43^ pipeline using default parameters to generate gene family, pathway abundance and pathway coverage outputs. Pathway abundance and coverage outputs were merged to identify pathways with greater than/equal to 50% gene family coverage within a given pathway. Pathways with greater than/equal to 50% coverage were further processed through linear discriminate analysis (LDA) effective size bio-discovery algorithm (LEfSe) (http://huttenhower.org/galaxy). Shannon diversity indices were calculated using the formula 
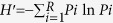
, where *Pi* represents the abundance of *i*^th^ pathway as a fraction of the sum of all pathway abundance.

### Scanning electron microscopy

Scanning electron microscopy was performed using a JSM 5400 SEM (JEOL) microscope (The Pennsylvania State University). Briefly, ilea were excised, flushed with sterile PBS and cut longitudinally. 5 mm^2^ sections were mounted on needles and fixed in 2% gluteraldehyde overnight at 4 °C. Samples were washed three times with 0.1 M sodium cacodylate buffer, pH 7.4, and then post fixed with 1% osmium tetroxide in 0.1 M sodium cacodylate buffer. The samples were washed and dehydrated through a graded ethanol series, then critical point dried, mounted, and coated with 10 nm gold palladium alloy. Images were acquired on a JSM 5400 SEM (JEOL).

### Quantitative PCR expression analysis

Mice were euthanized through carbon dioxide asphyxiation and terminal ilea (1 cm distal to cecal junction) or whole cecum excised and immediately frozen in liquid nitrogen and stored at −80 °C. Tissues were homogenized in 1 ml Tri Reagent (Sigma, St. Louis, MO.) together with 10–20 1 mm zirconia/silica beads (BioSpec Products, Bartletsville, OK) using a Bertin Precellys 24 homogenizer (VWR, Radnor, PA.) and total RNA isolated following manufacturers directions. 2 μg RNA were utilized as template for cDNA synthesis using the High Capacity cDNA Archive kit (Applied Biosystems, Foster City, CA.) following manufacturers directions. cDNA were diluted 1:10 in nuclease-free water and 6 μl used as a template for quantitative PCR. 20 μl reactions comprised template, 9 μl PerfeCTa SYBR mastermix (Quanta Biosciences, Gaithersburg, MD), 2 μl 3 nM forward primer, 2 μl 3 nM reverse primer and 1 μl 10 mg/ml BSA. Reactions were performed using the cycling conditions (95 °C, 3 min; 95 °C, 30 sec, target-specific annealing °C, 72 °C, 45 sec for 40 cycles; 72 °C, 5 min) on a CFX Connect platform (Biorad). Sequences of primers are described in Supplemental Material. Melt curve analysis of amplicons identified single melt peaks.

### ELISA

Mice were euthanized through carbon dioxide asphyxiation and terminal ilea (1 cm distal to cecal junction) excised and immediately frozen in liquid nitrogen and stored (−80 °C). Tissues were homogenized in PBS supplemented with complete mini protease inhibitor cocktail (Roche), followed by centrifugation (10,000 × g for 10 min at 4 °C). Supernatants were transferred to fresh tubes, normalized to 400 μg/ml protein and stored at −80 °C. Samples were shipped and analyzed (Eve Technologies, Calgary, Canada).

### ^1^H-NMR cecal metabolite analysis

^1^H-NMR analyses were performed as previously described^5^. Briefly, cecal content samples (~50 mg) were subjected to three consecutive freeze-thaws and directly extracted by precooled phosphate buffer with homogenization using the Precellys Tissue Homogenizer. After extraction, 550 μL of each extract was centrifuged and then transferred to 5 mm NMR tubes. Cecal extracts were recorded at 298 K on a Bruker Avance III 600 MHz spectrometer (Bruker Biospin, Germany) operating at 600.08 MHz for ^1^H, equipped with a Bruker inverse cryogenic probe.

### Cecal microbiota transfer

Female *Ahr*^−/+^ and *Ahr*^−/−^ littermates were derived from C57BL6/J × (B6.129-*Ahr*^*tm1Bra*^/J) matings and cohoused for 8 weeks prior to genotypic segregation. Following 14 days of segregation mice were euthanized and cecal contents extracted directly into anaerobic liquid dental medium (Anaerobe Systems, Morgan Hill, CA.). Cecal contents from 4 mice were pooled for each genotype to eliminate inter-animal variation in microbial composition. Pooled cecal contents were administered by gavage to 8 week-old germ-free C57BL6/J mice housed in isolation chambers within the Pennsylvania State University germ free facility. Mice were maintained within the germ free facility for 5 days under a standard 12 h light/dark cycle (light 0600–1800 h eastern standard time) with *ad libitum* access to sterile standard animal chow and water prior to euthanasia and sample collection.

### Statistical analysis

Where indicated, two-tailed unpaired parametric Students’ t-test was performed. Correlation analyses were performed using Pearson’s correlation coefficient (*r*) with a confidence interval of 95% and a two-tailed significance test. Significance thresholds of *p < 0.05, **p < 0.01 and ***p < 0.001 were applied. Statistical analyses and graphing was performed using Graphpad Prism v6.

## Additional Information

**How to cite this article**: Murray, I. A. *et al*. Expression of the aryl hydrocarbon receptor contributes to the establishment of intestinal microbial community structure in mice. *Sci. Rep.* **6**, 33969; doi: 10.1038/srep33969 (2016).

## Supplementary Material

Supplementary Information
