# Supplementary material for: Expression of the aryl hydrocarbon receptor contributes to the establishment of intestinal microbial community structure in mice
Source: Sci Rep. 2016 Sep 23;6:33969. doi: 10.1038/srep33969 (PMC5034278; doi:10.1038/srep33969)
Supplement: Supplementary Information [file srep33969-s1.pdf]

Expression of the aryl hydrocarbon receptor contributes to the establishment of intestinal microbial community structure in mice.

Iain A. Murray[1], Robert G. Nichols[1], Limin Zhang[1,2], Andrew D. Patterson[1] and Gary H. Perdew[1*].

[1]Center for Molecular Toxicology & Carcinogenesis Department of Veterinary and Biomedical Sciences, The Pennsylvania State University, University Park, PA-16802, USA

[2]Huck Institutes of the Life Sciences. The Pennsylvania State University, University Park, PA-16802, USA.

Supplementary information

Quantitative PCR oligonucleotide sequences

**Target**	**Forward (5′-3′)**	**Reverse (5′-3′)**
Saa1	acaccaggatgaagctactcacca	cccttggaaagcctcgtgaacaaa
Saa2	agctggctggaaagatggagacaa	tgtcctctgccgaagaattcctga
Saa3	atgccagagaggctgttcagaagt	tatcttttaggcaggccagcaggt
Cd14	ttcagaatctaccgaccatggagc	caattgaaagcgctggaccaa
Il17a	cagactacctcaaccgttccac	tccagctttccctccgcattga
Lcn2	atttcccagagtgaactggc	aatgtcacctccatcctggt
Rpl13a	ttcggctgaagcctaccagaaagt	gcatcttggcctttttccgtt
16S Eubacteria	actcctacgggaggcagcag	attaccgcggctgctgg
16S Firmicutes	gcagtagggaatcttccg	attaccgcggctgctgg
16S Bacteroidetes	gtactgagacacggacca	attaccgcggctgctgg
16S SFB	gacgctgcggcatgagagcat	gacggcacggattgttattca
16S A. muciniphila	cagcacgtgaaggtggggac	ccttgcggttggcttcagat

Supplemental figures

Supplemental figure 1

Supplemental figure 1. Experimental schematic. Illustration highlighting the overall methodologies utilized in this study.

Supplemental figure 2

Supplemental figure 2. Genotypic segregation of *Ahr* $^{-/+}$ and *Ahr* $^{-/-}$ littermates alters the community structure of the cecal microbiota. Abundance of individual microbiota phyla within the cecal luminal contents of genotypically segregated littermates (blue *Ahr* $^{-/+}$, red *Ahr* $^{-/-}$) as determined through 16S rDNA gene illumina MiSeq sequencing. Data represent min-max percentage abundance with median indicated by a horizontal bar (n = 4/genotype; $p < 0.05$*, $p < 0.01$** Student's t-test).

Supplemental figure 3

Supplemental figure 3. Quantitative analysis of cecal bacterial burden and phyla abundance in genotypically segregated *Ahr* $^{-/+}$ and *Ahr* $^{-/-}$ littermates. (**a**) Real time PCR quantification of bacterial burden and relative phyla abundance normalized to eubacteria using phyla-specific primers upon cecal microbial DNA from *Ahr*$^{-/+}$ (blue) and *Ahr*$^{-/-}$ (red) mice. (**b**) Real time PCR quantification of bacterial burden and relative phyla abundance normalized to eukaryotic *Rpl13a* using phyla-specific primers upon cDNA generated from total RNA isolated from intact cecal tissue (ceca and luminal contents) from *Ahr*$^{-/+}$ (blue) and *Ahr*$^{-/-}$ (red) mice. Data represent mean (n = 4/genotype) 16S

rDNA or rRNA gene abundance -/+ SD. Analyses were performed using Student's *t*-test $p < 0.05$*, $p < 0.01$**.

Supplemental figure 4

Supplemental figure 4. Analysis of correlative taxa-level interactions between cecal microbiota in genotypically segregated *Ahr* $^{-/+}$ and *Ahr* $^{-/-}$ mice. Heat map representations of correlative taxa-level interactions of cecal microbiota from genotypically segregated *Ahr* $^{-/+}$ and *Ahr* $^{-/-}$ littermates. Data represent Pearson correlation (*r*) coefficients with positive to negative correlation indicated by red to blue, respectively (n = 4/genotype). Correlation coefficients and *p* values are detailed in Supplementary file 1.

Supplemental file 1

Excel worksheets containing Pearson correlation coefficients and associated *p* values for taxa-taxa correlations. *Ahr* $^{-/+}$ genotype in column A (blue), *Ahr* $^{-/-}$ genotype in column A (red). Positive correlations are colored red, negative correlations are colored blue and those correlations with a $p < 0.05$ are presented in red text.

Supplemental file 2

Excel worksheets containing Pearson correlation coefficients and associated *p* values for taxa-cecal metabolite correlations. *Ahr* $^{-/+}$ genotype in column A (blue), *Ahr* $^{-/-}$ genotype in column A (red). Positive correlations are colored red, negative correlations are colored blue and those correlations with a $p < 0.05$ are presented in red text.

Supplemental figure 1

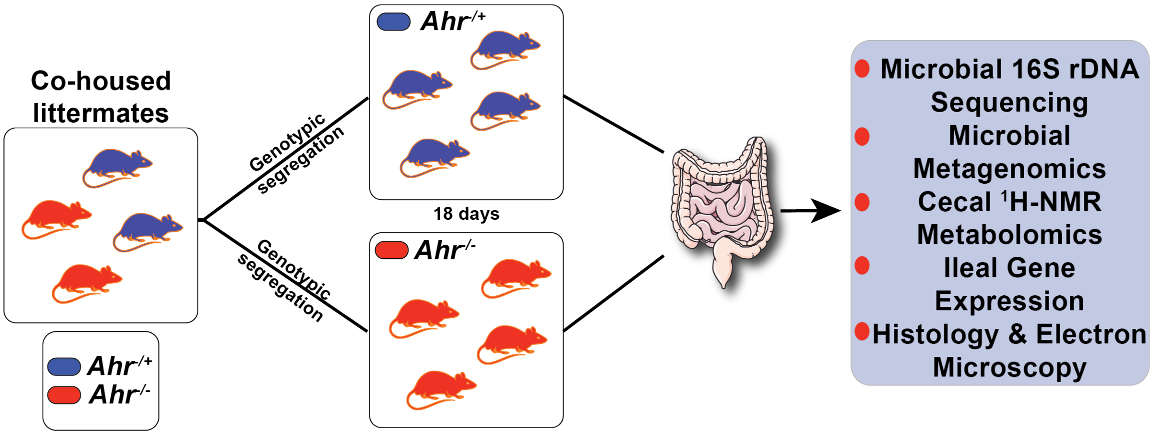

Supplemental figure 2

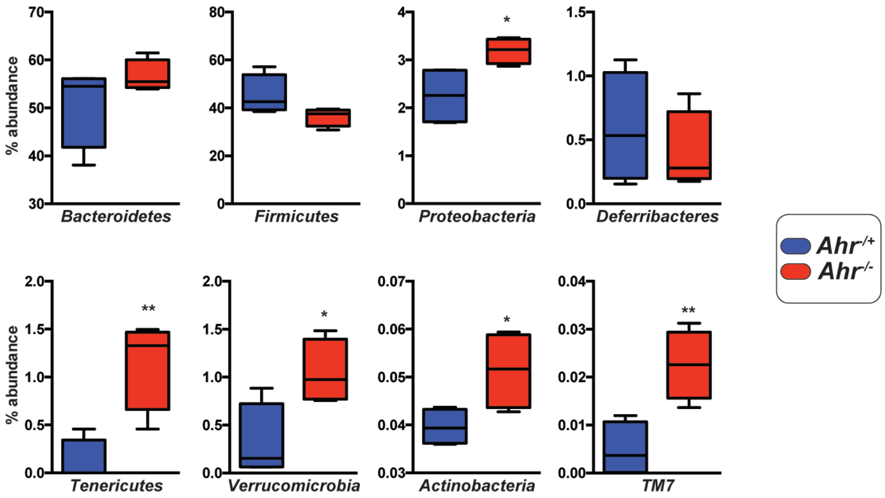

Supplemental figure 3

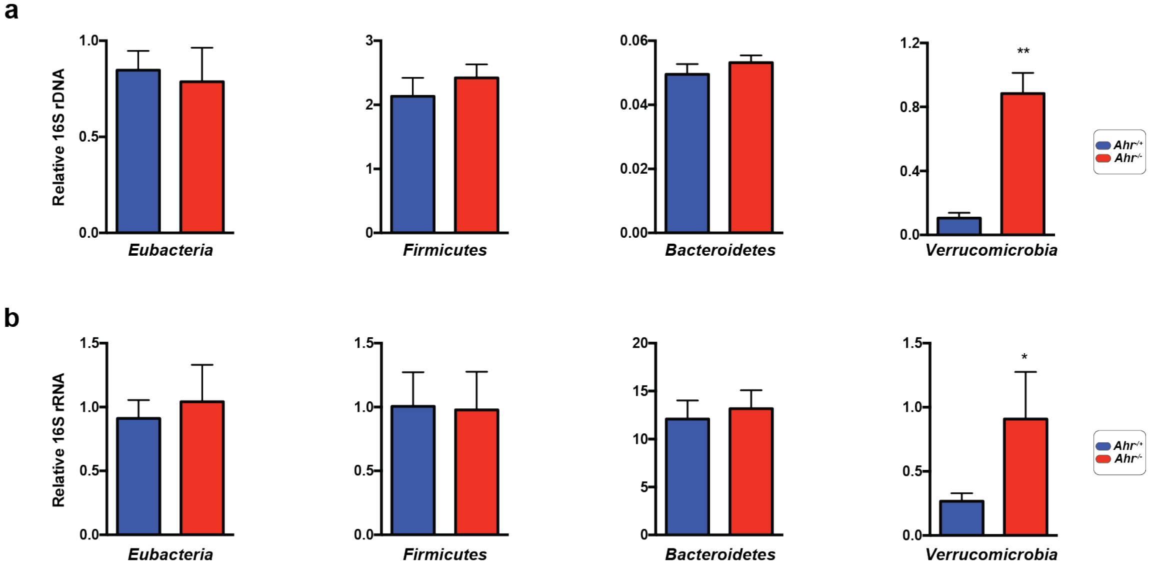

Supplemental figure 4

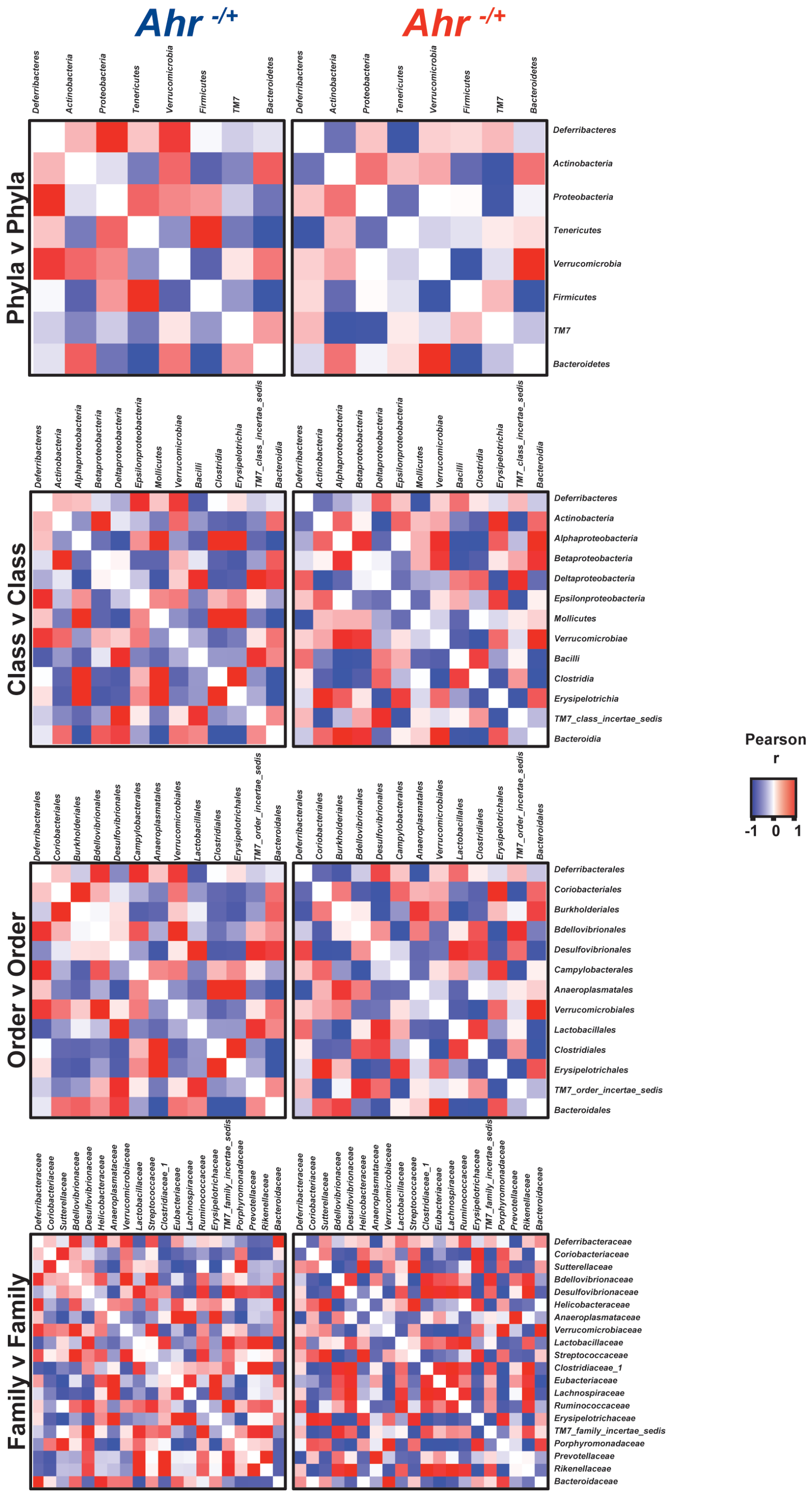

Ahr -/+
Phyla v Phyla Pearson correlation r

	"Deferribacteres"	"Actinobacteria"	"Proteobacteria"	"Tenericutes"	"Verrucomicrobia"	Firmicutes	TM7	"Bacteroidetes"
"Deferribacteres"		0.2787	0.8973	0.2273	0.7911	-0.02782	-0.196	-0.1136
"Actinobacteria"	0.2787		-0.1212	-0.4919	0.588	-0.6386	-0.453	0.6281
"Proteobacteria"	0.8973	-0.1212		0.5945	0.4601	0.3896	-0.205	-0.5206
"Tenericutes"	0.2273	-0.4919	0.5945		-0.4154	0.9639	-0.502	-0.9854
"Verrucomicrobia"	0.7911	0.588	0.4601	-0.4154		-0.6329	0.1103	0.5155
Firmicutes	-0.02782	-0.6386	0.3896	0.9639	-0.6329		-0.392	-0.9889
TM7	-0.1962	-0.4526	-0.2053	-0.5015	0.1103	-0.392		0.3755
"Bacteroidetes"	-0.1136	0.6281	-0.5206	-0.9854	0.5155	-0.9889	0.3755	

Ahr -/+
Phyla v Phyla Pearson correlation p

	"Deferribacteres"	"Actinobacteria"	"Proteobacteria"	"Tenericutes"	"Verrucomicrobia"	Firmicutes	TM7	"Bacteroidetes"
"Deferribacteres"		0.7213	0.1027	0.7727	0.2089	0.9722	0.8038	0.8864
"Actinobacteria"	0.7213		0.8788	0.5081	0.412	0.3614	0.5474	0.3719
"Proteobacteria"	0.1027	0.8788		0.4055	0.5399	0.6104	0.7947	0.4794
"Tenericutes"	0.7727	0.5081	0.4055		0.5846	0.0361	0.4985	0.0146
"Verrucomicrobia"	0.2089	0.412	0.5399	0.5846		0.3671	0.8897	0.4845
Firmicutes	0.9722	0.3614	0.6104	0.0361	0.3671		0.608	0.0111
TM7	0.8038	0.5474	0.7947	0.4985	0.8897	0.608		0.6245
"Bacteroidetes"	0.8864	0.3719	0.4794	0.0146	0.4845	0.0111	0.6245	

Ahr -/-
Phyla v Phyla Pearson correlation r

	"Deferribacteres"	"Actinobacteria"	"Proteobacteria"	"Tenericutes"	"Verrucomicrobia"	Firmicutes	TM7	"Bacteroidetes"
"Deferribacteres"		-0.5413	0.2258	-0.9361	0.1845	0.1543	0.2668	-0.1568
"Actinobacteria"	-0.5413		0.5513	0.2545	0.3398	-0.5689	-0.897	0.5442
"Proteobacteria"	0.2258	0.5513		-0.5533	-0.000918	0.01703	-0.855	-0.05405
"Tenericutes"	-0.9361	0.2545	-0.5533		-0.1768	-0.1185	0.0843	0.134
"Verrucomicrobia"	0.1845	0.3398	-0.000918	-0.1768		-0.9407	-0.121	0.9414
Firmicutes	0.1543	-0.5689	0.01703	-0.1185	-0.9407		0.2711	-0.9993
TM7	0.2668	-0.8969	-0.8549	0.08428	-0.1212	0.2711		-0.2365
"Bacteroidetes"	-0.1568	0.5442	-0.05405	0.134	0.9414	-0.9993	-0.237	

Ahr -/-
Phyla v Phyla Pearson correlation p

	"Deferribacteres"	"Actinobacteria"	"Proteobacteria"	"Tenericutes"	"Verrucomicrobia"	Firmicutes	TM7	"Bacteroidetes"
"Deferribacteres"		0.4587	0.7742	0.0639	0.8155	0.8457	0.7332	0.8432
"Actinobacteria"	0.4587		0.4487	0.7455	0.6602	0.4311	0.1031	0.4558
"Proteobacteria"	0.7742	0.4487		0.4467	0.9991	0.983	0.1451	0.946
"Tenericutes"	0.0639	0.7455	0.4467		0.8232	0.8815	0.9157	0.866
"Verrucomicrobia"	0.8155	0.6602	0.9991	0.8232		0.0593	0.8788	0.0586
Firmicutes	0.8457	0.4311	0.983	0.8815	0.0593		0.7289	0.0007
TM7	0.7332	0.1031	0.1451	0.9157	0.8788	0.7289		0.7635
"Bacteroidetes"	0.8432	0.4558	0.946	0.866	0.0586	0.0007	0.7635	

Ahr -/+

Class v Class Pearson correlation r

	Deferribacteres	Actinobacteria	Alphaproteobacteria	Betaproteobacteria	Deltaproteobacteria	Epsilonproteobacteria	unclassified	Mollicutes	Verrucomicrobiae	Bacilli	Clostridia	Erysipelotrichia	unclassified	TM7_class_incertae_sedis	unclassified	"Bacteroidia"	unclassified
Deferribacteres		0.2787	0.2458	-0.1303	-0.3163	0.8592	0.9449	0.2273	0.7911	-0.7039	-0.01016	0.1266	0.5268	-0.1962	-0.3852	-0.09197	-0.2293
Actinobacteria	0.2787		-0.4088	0.9157	-0.1019	-0.25	0.5579	-0.4919	0.588	-0.3932	-0.6122	-0.6639	-0.6563	-0.4526	-0.8688	0.5707	0.8594
Alphaproteobacteria	0.2458	-0.4088		-0.5393	-0.86	0.4278	-0.01533	0.9954	-0.3929	-0.6369	0.956	0.9536	0.6716	-0.5819	-0.0936	-0.979	-0.6607
Betaproteobacteria	-0.1303	0.9157	-0.5393		0.04369	-0.6164	0.1829	-0.6165	0.2847	-0.09972	-0.6413	-0.7501	-0.9003	-0.3695	-0.7275	0.6412	0.9856
Deltaproteobacteria	-0.3163	-0.1019	-0.86	0.04369		-0.2226	-0.1948	-0.8069	0.1969	0.8706	-0.7208	-0.6687	-0.2988	0.911	0.5811	0.7586	0.203
Epsilonproteobacteria	0.8592	-0.25	0.4278	-0.6164	-0.2226		0.6606	0.4554	0.5057	-0.4703	0.2806	0.4501	0.8722	0.08177	0.09391	-0.3612	-0.6803
unclassified	0.9449	0.5579	-0.01533	0.1829	-0.1948	0.6606		-0.05215	0.9195	-0.6503	-0.2926	-0.1807	0.224	-0.2108	-0.5532	0.1925	0.09554
Mollicutes	0.2273	-0.4919	0.9954	-0.6165	-0.8069	0.4554	-0.05215		-0.4154	-0.5742	0.9702	0.9777	0.7223	-0.5015	0.0007948	-0.9899	-0.7271
Verrucomicrobiae	0.7911	0.588	-0.3929	0.2847	0.1969	0.5057	0.9195	-0.4154		-0.3109	-0.6196	-0.5	0.02424	0.1103	-0.3829	0.5363	0.2591
Bacilli	-0.7039	-0.3932	-0.6369	-0.09972	0.8706	-0.4703	-0.6503	-0.5742	-0.3109		-0.3882	-0.3979	-0.3026	0.8275	0.7549	0.4667	0.06754
Clostridia	-0.01016	-0.6122	0.956	-0.6413	-0.7208	0.2806	-0.2926	0.9702	-0.6196	-0.3882		0.9814	0.6427	-0.422	0.1421	-0.9947	-0.7252
Erysipelotrichia	0.1266	-0.6639	0.9536	-0.7501	-0.6687	0.4501	-0.1807	0.9777	-0.5	-0.3979	0.9814		0.7779	-0.322	0.2103	-0.9882	-0.8314
unclassified	0.5268	-0.6563	0.6716	-0.9003	-0.2988	0.8722	0.224	0.7223	0.02424	-0.3026	0.6427	0.7779		0.1081	0.3883	-0.686	-0.9479
TM7_class_incertae_sedis	-0.1962	-0.4526	-0.5819	-0.3695	0.911	0.08177	-0.2108	-0.5015	0.1103	0.8275	-0.422	-0.322	0.1081		0.8237	0.4526	-0.2182
unclassified	-0.3852	-0.8688	-0.0936	-0.7275	0.5811	0.09391	-0.5532	0.0007948	-0.3829	0.7549	0.1421	0.2103	0.3883	0.8237		-0.08928	-0.6021
"Bacteroidia"	-0.09197	0.5707	-0.979	0.6412	0.7586	-0.3612	0.1925	-0.9899	0.5363	0.4667	-0.9947	-0.9882	-0.686	0.4526	-0.08928		0.7364
unclassified	-0.2293	0.8594	-0.6607	0.9856	0.203	-0.6803	0.09554	-0.7271	0.2591	0.06754	-0.7252	-0.8314	-0.9479	-0.2182	-0.6021	0.7364	

Ahr -/+

Class v Class Pearson correlation p

	Deferribacteres	Actinobacteria	Alphaproteobacteria	Betaproteobacteria	Deltaproteobacteria	Epsilonproteobacteria	unclassified	Mollicutes	Verrucomicrobiae	Bacilli	Clostridia	Erysipelotrichia	unclassified	TM7_class_incertae_sedis	unclassified	"Bacteroidia"	unclassified
Deferribacteres		0.7213	0.7542	0.8697	0.6837	0.1408	0.0551	0.7727	0.2089	0.2961	0.9898	0.8734	0.4732	0.8038	0.6148	0.908	0.7707
Actinobacteria	0.7213		0.5912	0.0843	0.8981	0.75	0.4421	0.5081	0.412	0.6068	0.3878	0.3361	0.3437	0.5474	0.1312	0.4293	0.1406
Alphaproteobacteria	0.7542	0.5912		0.4607	0.14	0.5722	0.9847	0.0046	0.6071	0.3631	0.044	0.0464	0.3284	0.4181	0.9064	0.021	0.3393
Betaproteobacteria	0.8697	0.0843	0.4607		0.9563	0.3836	0.8171	0.3835	0.7153	0.9003	0.3587	0.2499	0.0997	0.6305	0.2725	0.3588	0.0144
Deltaproteobacteria	0.6837	0.8981	0.14	0.9563		0.7774	0.8052	0.1931	0.8031	0.1294	0.2792	0.3313	0.7012	0.089	0.4189	0.2414	0.797
Epsilonproteobacteria	0.1408	0.75	0.5722	0.3836	0.7774		0.3394	0.5446	0.4943	0.5297	0.7194	0.5499	0.1278	0.9182	0.9061	0.6388	0.3197
unclassified	0.0551	0.4421	0.9847	0.8171	0.8052	0.3394		0.9478	0.0805	0.3497	0.7074	0.8193	0.776	0.7892	0.4468	0.8075	0.9045
Mollicutes	0.7727	0.5081	0.0046	0.3835	0.1931	0.5446	0.9478		0.5846	0.4258	0.0298	0.0223	0.2777	0.4985	0.9992	0.0101	0.2729
Verrucomicrobiae	0.2089	0.412	0.6071	0.7153	0.8031	0.4943	0.0805	0.5846		0.6891	0.3804	0.5	0.9758	0.8897	0.6171	0.4637	0.7409
Bacilli	0.2961	0.6068	0.3631	0.9003	0.1294	0.5297	0.3497	0.4258	0.6891		0.6118	0.6021	0.6974	0.1725	0.2451	0.5333	0.9325
Clostridia	0.9898	0.3878	0.044	0.3587	0.2792	0.7194	0.7074	0.0298	0.3804	0.6118		0.0186	0.3573	0.578	0.8579	0.0053	0.2748
Erysipelotrichia	0.8734	0.3361	0.0464	0.2499	0.3313	0.5499	0.8193	0.0223	0.5	0.6021	0.0186		0.2221	0.678	0.7897	0.0118	0.1686
unclassified	0.4732	0.3437	0.3284	0.0997	0.7012	0.1278	0.776	0.2777	0.9758	0.6974	0.3573	0.2221		0.8919	0.6117	0.314	0.0521
TM7_class_incertae_sedis	0.8038	0.5474	0.4181	0.6305	0.089	0.9182	0.7892	0.4985	0.8897	0.1725	0.578	0.678	0.8919		0.1763	0.5474	0.7818
unclassified	0.6148	0.1312	0.9064	0.2725	0.4189	0.9061	0.4468	0.9992	0.6171	0.2451	0.8579	0.7897	0.6117	0.1763		0.9107	0.3979
"Bacteroidia"	0.908	0.4293	0.021	0.3588	0.2414	0.6388	0.8075	0.0101	0.4637	0.5333	0.0053	0.0118	0.314	0.5474	0.9107		0.2636
unclassified	0.7707	0.1406	0.3393	0.0144	0.797	0.3197	0.9045	0.2729	0.7409	0.9325	0.2748	0.1686	0.0521	0.7818	0.3979	0.2636	

Ahr -/-

Class v Class Pearson correlation r

	Deferribacteres	Actinobacteria	Alphaproteobacteria	Betaproteobacteria	Deltaproteobacteria	Epsilonproteobacteria	unclassified	Mollicutes	Verrucomicrobiae	Bacilli	Clostridia	Erysipelotrichia	unclassified	TM7_class_incertae_sedis	unclassified	"Bacteroidia"	unclassified
Deferribacteres		-0.5413	-0.3233	-0.1208	0.6154	0.2252	-0.1963	-0.9361	0.1845	0.5539	0.09801	-0.1372	0.4121	0.2668	-0.3158	-0.2141	0.4333
Actinobacteria	-0.5413		0.6053	0.05037	-0.9958	0.6042	0.5606	0.2545	0.3398	-0.4786	-0.6451	0.9027	-0.4219	-0.8969	0.8218	0.5824	-0.7114
Alphaproteobacteria	-0.3233	0.6053		0.8199	-0.5953	0.009941	-0.3114	0.2953	0.8703	-0.9286	-0.9518	0.6303	-0.9546	-0.2596	0.935	0.9931	-0.9879
Betaproteobacteria	-0.1208	0.05037	0.8199		-0.05154	-0.4905	-0.7981	0.3006	0.7943	-0.8621	-0.6973	0.1009	-0.9189	0.3387	0.5689	0.8139	-0.7374
Deltaproteobacteria	0.6154	-0.9958	-0.5953	-0.05154		-0.546	-0.5538	-0.3381	-0.2912	0.5013	0.6091	-0.86	0.4322	0.8727	-0.7996	-0.5619	0.7075
Epsilonproteobacteria	0.2252	0.6042	0.009941	-0.4905	-0.546		0.7916	-0.5526	0.1176	0.3044	-0.2686	0.7735	0.2857	-0.8741	0.3498	0.06808	-0.07609
unclassified	-0.1963	0.5606	-0.3114	-0.7981	-0.5538	0.7916		-0.1284	-0.4347	0.4351	0.1758	0.4752	0.5113	-0.8285	0.03126	-0.3161	0.1817
Mollicutes	-0.9361	0.2545	0.2953	0.3006	-0.3381	-0.5526	-0.1284		-0.1768	-0.6025	-0.00965	-0.1482	-0.4764	0.08428	0.1641	0.1816	-0.3641
Verrucomicrobiae	0.1845	0.3398	0.8703	0.7943	-0.2912	0.1176	-0.4347	-0.1768		-0.6789	-0.9354	0.5753	-0.7798	-0.1212	0.8031	0.9197	-0.7995
Bacilli	0.5539	-0.4786	-0.9286	-0.8621	0.5013	0.3044	0.4351	-0.6025	-0.6789		0.7701	-0.3681	0.9869	0.05018	-0.7812	-0.8837	0.9244
Clostridia	0.09801	-0.6451	-0.9518	-0.6973	0.6091	-0.2686	0.1758	-0.009654	-0.9354	0.7701		-0.7794	0.8257	0.4048	-0.9619	-0.977	0.9344
Erysipelotrichia	-0.1372	0.9027	0.6303	0.1009	-0.86	0.7735	0.4752	-0.1482	0.5753	-0.3681	-0.7794		-0.3798	-0.8816	0.8623	0.6585	-0.6901
unclassified	0.4121	-0.4219	-0.9546	-0.9189	0.4322	0.2857	0.5113	-0.4764	-0.7798	0.9869	0.8257	-0.3798		0.006904	-0.7959	-0.9268	0.929
TM7_class_incertae_sedis	0.2668	-0.8969	-0.2596	0.3387	0.8727	-0.8741	-0.8285	0.08428	-0.1212	0.05018	0.4048	-0.8816	0.006904		-0.5808	-0.2661	0.3727
unclassified	-0.3158	0.8218	0.935	0.5689	-0.7996	0.3498	0.03126	0.1641	0.8031	-0.7812	-0.9619	0.8623	-0.7959	-0.5808		0.9382	-0.9602
"Bacteroidia"	-0.2141	0.5824	0.9931	0.8139	-0.5619	0.06808	-0.3161	0.1816	0.9197	-0.8837	-0.977	0.6585	-0.9268	-0.2661	0.9382		-0.9705
unclassified	0.4333	-0.7114	-0.9879	-0.7374	0.7075	-0.07609	0.1817	-0.3641	-0.7995	0.9244	0.9344	-0.6901	0.929	0.3727	-0.9602	-0.9705	

Ahr -/-

Class v Class Pearson correlation p

	Deferribacteres	Actinobacteria	Alphaproteobacteria	Betaproteobacteria	Deltaproteobacteria	Epsilonproteobacteria	unclassified	Mollicutes	Verrucomicrobiae	Bacilli	Clostridia	Erysipelotrichia	unclassified	TM7_class_incertae_sedis	unclassified	"Bacteroidia"	unclassified
Deferribacteres		0.4587	0.6767	0.8792	0.3846	0.7748	0.8037	0.0639	0.8155	0.4461	0.902	0.8628	0.5879	0.7332	0.6842	0.7859	0.5667
Actinobacteria	0.4587		0.3947	0.9496	0.0042	0.3958	0.4394	0.7455	0.6602	0.5214	0.3549	0.0973	0.5781	0.1031	0.1782	0.4176	0.2886
Alphaproteobacteria	0.6767	0.3947		0.1801	0.4047	0.9901	0.6886	0.7047	0.1297	0.0714	0.0482	0.3697	0.0454	0.7404	0.065	0.0069	0.0121
Betaproteobacteria	0.8792	0.9496	0.1801		0.9485	0.5095	0.2019	0.6994	0.2057	0.1379	0.3027	0.8991	0.0811	0.6613	0.4311	0.1861	0.2626
Deltaproteobacteria	0.3846	0.0042	0.4047	0.9485		0.454	0.4462	0.6619	0.7088	0.4987	0.3909	0.14	0.5678	0.1273	0.2004	0.4381	0.2925
Epsilonproteobacteria	0.7748	0.3958	0.9901	0.5095	0.454		0.2084	0.4474	0.8824	0.6956	0.7314	0.2265	0.7143	0.1259	0.6502	0.9319	0.9239
unclassified	0.8037	0.4394	0.6886	0.2019	0.4462	0.2084		0.8716	0.5653	0.5649	0.8242	0.5248	0.4887	0.1715	0.9687	0.6839	0.8183
Mollicutes	0.0639	0.7455	0.7047	0.6994	0.6619	0.4474	0.8716		0.8232	0.3975	0.9903	0.8518	0.5236	0.9157	0.8359	0.8184	0.6359
Verrucomicrobiae	0.8155	0.6602	0.1297	0.2057	0.7088	0.8824	0.5653	0.8232		0.3211	0.0646	0.4247	0.2202	0.8788	0.1969	0.0803	0.2005
Bacilli	0.4461	0.5214	0.0714	0.1379	0.4987	0.6956	0.5649	0.3975	0.3211		0.2299	0.6319	0.0131	0.9498	0.2188	0.1163	0.0756
Clostridia	0.902	0.3549	0.0482	0.3027	0.3909	0.7314	0.8242	0.9903	0.0646	0.2299		0.2206	0.1743	0.5952	0.0381	0.023	0.0656
Erysipelotrichia	0.8628	0.0973	0.3697	0.8991	0.14	0.2265	0.5248	0.8518	0.4247	0.6319	0.2206		0.6202	0.1184	0.1377	0.3415	0.3099
unclassified	0.5879	0.5781	0.0454	0.0811	0.5678	0.7143	0.4887	0.5236	0.2202	0.0131	0.1743	0.6202		0.9931	0.2041	0.0732	0.071
TM7_class_incertae_sedis	0.7332	0.1031	0.7404	0.6613	0.1273	0.1259	0.1715	0.9157	0.8788	0.9498	0.5952	0.1184	0.9931		0.4192	0.7339	0.6273
unclassified	0.6842	0.1782	0.065	0.4311	0.2004	0.6502	0.9687	0.8359	0.1969	0.2188	0.0381	0.1377	0.2041	0.4192		0.0618	0.0398
"Bacteroidia"	0.7859	0.4176	0.0069	0.1861	0.4381	0.9319	0.6839	0.8184	0.0803	0.1163	0.023	0.3415	0.0732	0.7339	0.0618		0.0295
unclassified	0.5667	0.2886	0.0121	0.2626	0.2925	0.9239	0.8183	0.6359	0.2005	0.0756	0.0656	0.3099	0.071	0.6273	0.0398	0.0295	

Ahr -/+

Order v Order Pearson correlation r

	Deferribacterales	Coriobacteriales	unclassified	Burkholderiales	unclassified	Bdellovibrionales	unclassified	Desulfovibrionales	Campylobacterales	unclassified	Anaeroplasmatales	Verrucomicrobiales	Lactobacillales	Clostridiales	unclassified	Erysipelotrichales	unclassified	TM7_order_incertae_sedis	unclassified	"Bacteroidales"	unclassified
**Deferribacterales**		0.223	0.247	-0.2014	0.1667	0.8252	-0.3925	-0.4447	0.8592	0.9449	0.2273	0.7911	-0.7058	-0.01506	0.5632	0.1266	0.5268	-0.1962	-0.3852	-0.09197	-0.2293
**Coriobacteriales**	0.223		-0.3966	0.9095	0.9842	0.2981	-0.6141	-0.09007	-0.3066	0.5047	-0.4806	0.5298	-0.3876	-0.5854	-0.1027	-0.6535	-0.6909	-0.4834	-0.8773	0.5526	0.8791
**unclassified**	0.247	-0.3966		-0.5297	-0.5523	-0.3207	-0.4803	-0.8764	0.4296	-0.01442	0.9955	-0.3918	-0.6333	0.9555	-0.4268	0.954	0.6732	-0.5806	-0.09243	-0.9791	-0.6621
**Burkholderiales**	-0.2014	0.9095	-0.5297		0.9228	-0.03444	-0.4267	0.1264	-0.6727	0.109	-0.6043	0.2108	-0.06837	-0.6064	-0.3182	-0.732	-0.9237	-0.375	-0.7036	0.619	0.9863
**unclassified**	0.1667	0.9842	-0.5523	0.9228		0.3444	-0.4648	0.08577	-0.3495	0.472	-0.6275	0.567	-0.2346	-0.7176	-0.000198	-0.7768	-0.7492	-0.3242	-0.779	0.6912	0.9212
**Bdellovibrionales**	0.8252	0.2981	-0.3207	-0.03444	0.3444		0.0242	0.129	0.6618	0.8853	-0.3222	0.9675	-0.2411	-0.5255	0.8752	-0.3661	0.2345	0.2664	-0.1705	0.437	0.02856
**unclassified**	-0.3925	-0.6141	-0.4803	-0.4267	-0.4648	0.0242		0.8363	-0.04422	-0.4393	-0.3956	-0.1395	0.9018	-0.2687	0.4954	-0.1956	0.1021	0.9688	0.9163	0.3168	-0.2825
**Desulfovibrionales**	-0.4447	-0.09007	-0.8764	0.1264	0.08577	0.129	0.8363		-0.3665	-0.3037	-0.829	0.09282	0.9098	-0.7172	0.4571	-0.6929	-0.4092	0.8629	0.5567	0.7662	0.2828
**Campylobacterales**	0.8592	-0.3066	0.4296	-0.6727	-0.3495	0.6618	-0.04422	-0.3665		0.6606	0.4554	0.5057	-0.4705	0.2739	0.6217	0.4501	0.8722	0.08177	0.09391	-0.3612	-0.6803
**unclassified**	0.9449	0.5047	-0.01442	0.109	0.472	0.8853	-0.4393	-0.3037	0.6606		-0.05215	0.9195	-0.6534	-0.2962	0.5622	-0.1807	0.224	-0.2108	-0.5532	0.1925	0.09554
**Anaeroplasmatales**	0.2273	-0.4806	0.9955	-0.6043	-0.6275	-0.3222	-0.3956	-0.829	0.4554	-0.05215		-0.4154	-0.5707	0.9693	-0.3839	0.9777	0.7223	-0.5015	0.0007948	-0.9899	-0.7271
**Verrucomicrobiales**	0.7911	0.5298	-0.3918	0.2108	0.567	0.9675	-0.1395	0.09282	0.5057	0.9195	-0.4154		-0.3149	-0.6235	0.7511	-0.5	0.02424	0.1103	-0.3829	0.5363	0.2591
**Lactobacillales**	-0.7058	-0.3876	-0.6333	-0.06837	-0.2346	-0.2411	0.9018	0.9098	-0.4705	-0.6534	-0.5707	-0.3149		-0.384	0.187	-0.394	-0.3007	0.8265	0.7567	0.4628	0.06469
**Clostridiales**	-0.01506	-0.5854	0.9555	-0.6064	-0.7176	-0.5255	-0.2687	-0.7172	0.2739	-0.2962	0.9693	-0.6235	-0.384		-0.5042	0.9799	0.6369	-0.4261	0.1386	-0.9942	-0.7202
**unclassified**	0.5632	-0.1027	-0.4268	-0.3182	-0.000198	0.8752	0.4954	0.4571	0.6217	0.5622	-0.3839	0.7511	0.187	-0.5042		-0.3224	0.343	0.6851	0.3209	0.4474	-0.2005
**Erysipelotrichales**	0.1266	-0.6535	0.954	-0.732	-0.7768	-0.3661	-0.1956	-0.6929	0.4501	-0.1807	0.9777	-0.5	-0.394	0.9799	-0.3224		0.7779	-0.322	0.2103	-0.9882	-0.8314
**unclassified**	0.5268	-0.6909	0.6732	-0.9237	-0.7492	0.2345	0.1021	-0.4092	0.8722	0.224	0.7223	0.02424	-0.3007	0.6369	0.343	0.7779		0.1081	0.3883	-0.686	-0.9479
**TM7_order_incertae_sedis**	-0.1962	-0.4834	-0.5806	-0.375	-0.3242	0.2664	0.9688	0.8629	0.08177	-0.2108	-0.5015	0.1103	0.8265	-0.4261	0.6851	-0.322	0.1081		0.8237	0.4526	-0.2182
**unclassified**	-0.3852	-0.8773	-0.09243	-0.7036	-0.779	-0.1705	0.9163	0.5567	0.09391	-0.5532	0.0007948	-0.3829	0.7567	0.1386	0.3209	0.2103	0.3883	0.8237		-0.08928	-0.6021
**"Bacteroidales"**	-0.09197	0.5526	-0.9791	0.619	0.6912	0.437	0.3168	0.7662	-0.3612	0.1925	-0.9899	0.5363	0.4628	-0.9942	0.4474	-0.9882	-0.686	0.4526	-0.08928		0.7364
**unclassified**	-0.2293	0.8791	-0.6621	0.9863	0.9212	0.02856	-0.2825	0.2828	-0.6803	0.09554	-0.7271	0.2591	0.06469	-0.7202	-0.2005	-0.8314	-0.9479	-0.2182	-0.6021	0.7364	

Ahr -/+

Class v Class Pearson correlation p

	Deferribacterales	Coriobacteriales	unclassified	Burkholderiales	unclassified	Bdellovibrionales	unclassified	Desulfovibrionales	Campylobacterales	unclassified	Anaeroplasmatales	Verrucomicrobiales	Lactobacillales	Clostridiales	unclassified	Erysipelotrichales	unclassified	TM7_order_incertae_sedis	unclassified	"Bacteroidales"	unclassified
**Deferribacterales**		0.777	0.753	0.7986	0.8333	0.1748	0.6075	0.5553	0.1408	0.0551	0.7727	0.2089	0.2942	0.9849	0.4368	0.8734	0.4732	0.8038	0.6148	0.908	0.7707
**Coriobacteriales**	0.777		0.6034	0.0905	0.0158	0.7019	0.3859	0.9099	0.6934	0.4953	0.5194	0.4702	0.6124	0.4146	0.8973	0.3465	0.3091	0.5166	0.1227	0.4474	0.1209
**unclassified**	0.753	0.6034		0.4703	0.4477	0.6793	0.5197	0.1236	0.5704	0.9856	0.0045	0.6082	0.3667	0.0445	0.5732	0.046	0.3268	0.4194	0.9076	0.0209	0.3379
**Burkholderiales**	0.7986	0.0905	0.4703		0.0772	0.9656	0.5733	0.8736	0.3273	0.891	0.3957	0.7892	0.9316	0.3936	0.6818	0.268	0.0763	0.625	0.2964	0.381	0.0137
**unclassified**	0.8333	0.0158	0.4477	0.0772		0.6556	0.5352	0.9142	0.6505	0.528	0.3725	0.433	0.7654	0.2824	0.9998	0.2232	0.2508	0.6758	0.221	0.3088	0.0788
**Bdellovibrionales**	0.1748	0.7019	0.6793	0.9656	0.6556		0.9758	0.871	0.3382	0.1147	0.6778	0.0325	0.7589	0.4745	0.1248	0.6339	0.7655	0.7336	0.8295	0.563	0.9714
**unclassified**	0.6075	0.3859	0.5197	0.5733	0.5352	0.9758		0.1637	0.9558	0.5607	0.6044	0.8605	0.0982	0.7313	0.5046	0.8044	0.8979	0.0312	0.0837	0.6832	0.7175
**Desulfovibrionales**	0.5553	0.9099	0.1236	0.8736	0.9142	0.871	0.1637		0.6335	0.6963	0.171	0.9072	0.0902	0.2828	0.5429	0.3071	0.5908	0.1371	0.4433	0.2338	0.7172
**Campylobacterales**	0.1408	0.6934	0.5704	0.3273	0.6505	0.3382	0.9558	0.6335		0.3394	0.5446	0.4943	0.5295	0.7261	0.3783	0.5499	0.1278	0.9182	0.9061	0.6388	
**unclassified**	0.0551	0.4953	0.9856	0.891	0.528	0.1147	0.5607	0.6963	0.3394		0.9478	0.0805	0.3466	0.7038	0.4378	0.8193	0.776	0.7892	0.4468	0.8075	0.9045
**Anaeroplasmatales**	0.7727	0.5194	0.0045	0.3957	0.3725	0.6778	0.6044	0.171	0.5446	0.9478		0.5846	0.4293	0.0307	0.6161	0.0223	0.2777	0.4985	0.9992	0.0101	0.2729
**Verrucomicrobiales**	0.2089	0.4702	0.6082	0.7892	0.433	0.0325	0.8605	0.9072	0.4943	0.0805	0.5846		0.6851	0.3765	0.2489	0.5	0.9758	0.8897	0.6171	0.4637	0.7409
**Lactobacillales**	0.2942	0.6124	0.3667	0.9316	0.7654	0.7589	0.0982	0.0902	0.5295	0.3466	0.4293	0.6851		0.616	0.813	0.606	0.6993	0.1735	0.2433	0.5372	0.9353
**Clostridiales**	0.9849	0.4146	0.0445	0.3936	0.2824	0.4745	0.7313	0.2828	0.7261	0.7038	0.0307	0.3765	0.616		0.4958	0.0201	0.3631	0.5739	0.8614	0.0058	0.2798
**unclassified**	0.4368	0.8973	0.5732	0.6818	0.9998	0.1248	0.5046	0.5429	0.3783	0.4378	0.6161	0.2489	0.813	0.4958		0.6776	0.657	0.3149	0.6791	0.5526	0.7995
**Erysipelotrichales**	0.8734	0.3465	0.046	0.268	0.2232	0.6339	0.8044	0.3071	0.5499	0.8193	0.0223	0.5	0.606	0.0201	0.6776		0.2221	0.678	0.7897	0.0118	0.1686
**unclassified**	0.4732	0.3091	0.3268	0.0763	0.2508	0.7655	0.8979	0.5908	0.1278	0.776	0.2777	0.9758	0.6993	0.3631	0.657	0.2221		0.8919	0.6117	0.314	0.0521
**TM7_order_incertae_sedis**	0.8038	0.5166	0.4194	0.625	0.6758	0.7336	0.0312	0.1371	0.9182	0.7892	0.4985	0.8897	0.1735	0.5739	0.3149	0.678	0.8919		0.1763	0.5474	0.7818
**unclassified**	0.6148	0.1227	0.9076	0.2964	0.221	0.8295	0.0837	0.4433	0.9061	0.4468	0.9992	0.6171	0.2433	0.8614	0.6791	0.7897	0.6117	0.1763		0.9107	0.3979
**"Bacteroidales"**	0.908	0.4474	0.0209	0.381	0.3088	0.563	0.6832	0.2338	0.6388	0.8075	0.0101	0.4637	0.5372	0.0058	0.5526	0.0118	0.314	0.5474	0.9107		0.2636
**unclassified**	0.7707	0.1209	0.3379	0.0137	0.0788	0.9714	0.7175	0.7172	0.3197	0.9045	0.2729	0.7409	0.9353	0.2798	0.7995	0.1686	0.0521	0.7818	0.3979	0.2636	

Ahr -/-

Order v Order Pearson correlation r

	Deferribacterales	Coriobacteriales	unclassified	Burkholderiales	unclassified	Bdellovibrionales	unclassified	Desulfovibrionales	Campylobacterales	unclassified	Anaeroplasmatales	Verrucomicrobiales	Lactobacillales	Clostridiales	unclassified	Erysipelotrichales	unclassified	TM7_order_incertae_sedis	unclassified	"Bacteroidales"	unclassified
**Deferribacterales**		-0.5694	-0.3167	-0.7429	0.005748	-0.1997	0.9905	0.7118	0.2252	-0.1963	-0.9362	0.1845	0.5536	0.07766	0.8701	-0.1372	0.4121	0.2668	-0.3158	-0.2141	0.4333
**Coriobacteriales**	-0.5694		0.5984	0.4981	-0.0301	-0.6914	-0.602	-0.8766	0.5799	0.5528	0.287	0.3279	-0.4932	-0.6272	-0.766	0.8884	-0.4324	-0.8862	0.8182	0.581	-0.7156
**unclassified**	-0.3167	0.5984		0.8127	0.7818	-0.4132	-0.2334	-0.8419	0.002247	-0.3222	0.2919	0.8738	-0.9293	-0.9463	-0.7367	0.6233	-0.9565	-0.2491	0.9314	0.9935	-0.986
**Burkholderiales**	-0.7429	0.4981	0.8127		0.6556	0.0769	-0.6541	-0.8502	-0.3905	-0.3495	0.7904	0.4613	-0.9643	-0.5872	-0.9197	0.257	-0.9109	-0.03985	0.667	0.7415	-0.8367
**unclassified**	0.005748	-0.0301	0.7818	0.6556		0.0579	0.1367	-0.3827	-0.4818	-0.839	0.1887	0.8132	-0.7934	-0.6768	-0.351	0.0651	-0.8681	0.3922	0.5176	0.7815	-0.6763
**Bdellovibrionales**	-0.1997	-0.6914	-0.4132	0.0769	0.0579		-0.1491	0.4085	-0.901	-0.5085	0.4852	-0.5303	0.07973	0.6601	0.1387	-0.9354	0.1295	0.8315	-0.6829	-0.4826	0.4532
**unclassified**	0.9905	-0.602	-0.2334	-0.6541	0.1367	-0.1491		0.6789	0.1242	-0.3256	-0.8895	0.266	0.4541	0.01884	0.8295	-0.1687	0.3054	0.3524	-0.278	-0.134	0.365
**Desulfovibrionales**	0.7118	-0.8766	-0.8419	-0.8502	-0.3827	0.4085	0.6789		-0.149	-0.1143	-0.5695	-0.5073	0.8445	0.7462	0.9569	-0.7026	0.7878	0.5553	-0.8912	-0.7978	0.9186
**Campylobacterales**	0.2252	0.5799	0.002247	-0.3905	-0.4818	-0.901	0.1242	-0.149		0.7916	-0.5526	0.1176	0.3042	-0.2744	0.0794	0.7735	0.2857	-0.8741	0.3498	0.06808	-0.07609
**unclassified**	-0.1963	0.5528	-0.3222	-0.3495	-0.839	-0.5085	-0.3256	-0.1143	0.7916		-0.1281	-0.4347	0.4351	0.1795	-0.04627	0.4752	0.5113	-0.8285	0.03126	-0.3161	0.1817
**Anaeroplasmatales**	-0.9362	0.287	0.2919	0.7904	0.1887	0.4852	-0.8895	-0.5695	-0.5526	-0.1281		-0.1774	-0.6017	0.01037	-0.7837	-0.1485	-0.4759	0.08436	0.1636	0.181	-0.3635
**Verrucomicrobiales**	0.1845	0.3279	0.8738	0.4613	0.8132	-0.5303	0.266	-0.5073	0.1176	-0.4347	-0.1774		-0.6791	-0.9407	-0.3173	0.5753	-0.7798	-0.1212	0.8031	0.9197	-0.7995
**Lactobacillales**	0.5536	-0.4932	-0.9293	-0.9643	-0.7934	0.07973	0.4541	0.8445	0.3042	0.4351	-0.6017	-0.6791		0.76	0.848	-0.3683	0.9869	0.05023	-0.7813	-0.8839	0.9245
**Clostridiales**	0.07766	-0.6272	-0.9463	-0.5872	-0.6768	0.6601	0.01884	0.7462	-0.2744	0.1795	0.01037	-0.9407	0.76		0.5581	-0.7784	0.8184	0.4007	-0.9573	-0.9743	0.9271
**unclassified**	0.8701	-0.766	-0.7367	-0.9197	-0.351	0.1387	0.8295	0.9569	0.0794	-0.04627	-0.7837	-0.3173	0.848	0.5581		-0.4798	0.7602	0.3934	-0.7304	-0.6641	0.8207
**Erysipelotrichales**	-0.1372	0.8884	0.6233	0.257	0.0651	-0.9354	-0.1687	-0.7026	0.7735	0.4752	-0.1485	0.5753	-0.3683	-0.7784	-0.4798		-0.3798	-0.8816	0.8623	0.6585	-0.6901
**unclassified**	0.4121	-0.4324	-0.9565	-0.9109	-0.8681	0.1295	0.3054	0.7878	0.2857	0.5113	-0.4759	-0.7798	0.9869	0.8184	0.7602	-0.3798		0.006904	-0.7959	-0.9268	0.929
**TM7_order_incertae_sedis**	0.2668	-0.8862	-0.2491	-0.03985	0.3922	0.8315	0.3524	0.5553	-0.8741	-0.8285	0.08436	-0.1212	0.05023	0.4007	0.3934	-0.8816	0.006904		-0.5808	-0.2661	0.3727
**unclassified**	-0.3158	0.8182	0.9314	0.667	0.5176	-0.6829	-0.278	-0.8912	0.3498	0.03126	0.1636	0.8031	-0.7813	-0.9573	-0.7304	0.8623	-0.7959	-0.5808		0.9382	-0.9602
**"Bacteroidales"**	-0.2141	0.581	0.9935	0.7415	0.7815	-0.4826	-0.134	-0.7978	0.06808	-0.3161	0.181	0.9197	-0.8839	-0.9743	-0.6641	0.6585	-0.9268	-0.2661	0.9382		-0.9705
**unclassified**	0.4333	-0.7156	-0.986	-0.8367	-0.6763	0.4532	0.365	0.9186	-0.07609	0.1817	-0.3635	-0.7995	0.9245	0.9271	0.8207	-0.6901	0.929	0.3727	-0.9602	-0.9705	

Ahr -/-

Class v Class Pearson correlation p

	Deferribacterales	Coriobacteriales	unclassified	Burkholderiales	unclassified	Bdellovibrionales	unclassified	Desulfovibrionales	Campylobacterales	unclassified	Anaeroplasmatales	Verrucomicrobiales	Lactobacillales	Clostridiales	unclassified	Erysipelotrichales	unclassified	TM7_order_incertae_sedis	unclassified	"Bacteroidales"	unclassified
**Deferribacterales**		0.4306	0.6833	0.2571	0.9943	0.8003	0.0095	0.2882	0.7748	0.8037	0.0638	0.8155	0.4464	0.9223	0.1299	0.8628	0.5879	0.7332	0.6842	0.7859	0.5667
**Coriobacteriales**	0.4306		0.4016	0.5019	0.9699	0.3086	0.398	0.1234	0.4201	0.4472	0.713	0.6721	0.5068	0.3728	0.234	0.1116	0.5676	0.1138	0.1818	0.419	0.2844
**unclassified**	0.6833	0.4016		0.1873	0.2182	0.5868	0.7666	0.1581	0.9978	0.6778	0.7081	0.1262	0.0707	0.0537	0.2633	0.3767	0.0435	0.7509	0.0686	0.0065	0.014
**Burkholderiales**	0.2571	0.5019	0.1873		0.3444	0.9231	0.3459	0.1498	0.6095	0.6505	0.2096	0.5387	0.0357	0.4128	0.0803	0.743	0.0891	0.9602	0.333	0.2585	0.1633
**unclassified**	0.9943	0.9699	0.2182	0.3444		0.9421	0.8633	0.6173	0.5182	0.161	0.8113	0.1868	0.2066	0.3232	0.649	0.9349	0.1319	0.6078	0.4824	0.2185	0.3237
**Bdellovibrionales**	0.8003	0.3086	0.5868	0.9231	0.9421		0.8509	0.5915	0.099	0.4915	0.5148	0.4697	0.9203	0.3399	0.8613	0.0646	0.8705	0.1685	0.3171	0.5174	0.5468
**unclassified**	0.0095	0.398	0.7666	0.3459	0.8633	0.8509		0.3211	0.8758	0.6744	0.1105	0.734	0.5459	0.9812	0.1705	0.8313	0.6946	0.6476	0.722	0.866	0.635
**Desulfovibrionales**	0.2882	0.1234	0.1581	0.1498	0.6173	0.5915	0.3211		0.851	0.8857	0.4305	0.4927	0.1555	0.2538	0.0431	0.2974	0.2122	0.4447	0.1088	0.2022	0.0814
**Campylobacterales**	0.7748	0.4201	0.9978	0.6095	0.5182	0.099	0.8758	0.851		0.2084	0.4474	0.8824	0.6958	0.7256	0.9206	0.2265	0.7143	0.1259	0.6502	0.9319	0.9239
**unclassified**	0.8037	0.4472	0.6778	0.6505	0.161	0.4915	0.6744	0.8857	0.2084		0.8719	0.5653	0.5649	0.8205	0.9537	0.5248	0.4887	0.1715	0.9687	0.6839	0.8183
**Anaeroplasmatales**	0.0638	0.713	0.7081	0.2096	0.8113	0.5148	0.1105	0.4305	0.4474	0.8719		0.8226	0.3983	0.9896	0.2163	0.8515	0.5241	0.9156	0.8364	0.819	0.6365
**Verrucomicrobiales**	0.8155	0.6721	0.1262	0.5387	0.1868	0.4697	0.734	0.4927	0.8824	0.5653	0.8226		0.3209	0.0593	0.6827	0.4247	0.2202	0.8788	0.1969	0.0803	0.2005
**Lactobacillales**	0.4464	0.5068	0.0707	0.0357	0.2066	0.9203	0.5459	0.1555	0.6958	0.5649	0.3983	0.3209		0.24	0.152	0.6317	0.0131	0.9498	0.2187	0.1161	0.0755
**Clostridiales**	0.9223	0.3728	0.0537	0.4128	0.3232	0.3399	0.9812	0.2538	0.7256	0.8205	0.9896	0.0593	0.24		0.4419	0.2216	0.1816	0.5993	0.0427	0.0257	0.0729
**unclassified**	0.1299	0.234	0.2633	0.0803	0.649	0.8613	0.1705	0.0431	0.9206	0.9537	0.2163	0.6827	0.152	0.4419		0.5202	0.2398	0.6066	0.2696	0.3359	0.1793
**Erysipelotrichales**	0.8628	0.1116	0.3767	0.743	0.9349	0.0646	0.8313	0.2974	0.2265	0.5248	0.8515	0.4247	0.6317	0.2216	0.5202		0.6202	0.1184	0.1377	0.3415	0.3099
**unclassified**	0.5879	0.5676	0.0435	0.0891	0.1319	0.8705	0.6946	0.2122	0.7143	0.4887	0.5241	0.2202	0.0131	0.1816	0.2398	0.6202		0.9931	0.2041	0.0732	0.071
**TM7_order_incertae_sedis**	0.7332	0.1138	0.7509	0.9602	0.6078	0.1685	0.6476	0.4447	0.1259	0.1715	0.9156	0.8788	0.9498	0.5993	0.6066	0.1184	0.9931		0.4192	0.7339	0.6273
**unclassified**	0.6842	0.1818	0.0686	0.333	0.4824	0.3171	0.722	0.1088	0.6502	0.9687	0.8364	0.1969	0.2187	0.0427	0.2696	0.1377	0.2041	0.4192		0.0618	0.0398
**"Bacteroidales"**	0.7859	0.419	0.0065	0.2585	0.2185	0.5174	0.866	0.2022	0.9319	0.6839	0.819	0.0803	0.1161	0.0257	0.3359	0.3415	0.0732	0.7339	0.0618		0.0295
**unclassified**	0.5667	0.2844	0.014	0.1633	0.3237	0.5468	0.635	0.0814	0.9239	0.8183	0.6365	0.2005	0.0755	0.0729	0.1793	0.3099	0.071	0.6273	0.0398	0.0295	

Ahr -/+
Family v Family Pearson correlation r

	Deferribacteraceae	Coriobacteriaceae	unclassified	Sutterellaceae	unclassified	unclassified	Bdellovibrionaceae	unclassified	Desulfovibrionaceae	unclassified	Helicobacteraceae	unclassified	Anaeroplasmataceae	Verrucomicrobiaceae	Lactobacillaceae	Streptococcaceae	unclassified	Clostridiaceae_1	Eubacteriaceae	Lachnospiraceae	Ruminococcaceae	unclassified	unclassified	Erysipelotrichaceae	unclassified	TM7_family_incertae_sedis	unclassified	"Porphyromonadaceae"	"Prevotellaceae"	"Rikenellaceae"	Bacteroidaceae	unclassified	unclassified
Deferribacteraceae		0.223	0.247	-0.09676	-0.2854	0.1667	0.8294	-0.3925	-0.3138	-0.9406	0.8591	0.9449	0.2273	0.7911	-0.7001	0.686	-0.7263	-0.6939	0.3693	-0.1372	0.1792	0.9214	0.5632	0.1266	0.5268	-0.1962	-0.3852	-0.4109	-0.6142	-0.5797	0.9414	0.7024	-0.2293
Coriobacteriaceae	0.223		-0.3966	0.8473	0.6149	0.9842	0.3036	-0.6141	-0.1162	0.1197	-0.3069	0.5047	-0.4806	0.5298	-0.4002	0.06681	0.4962	-0.8318	-0.6088	-0.4786	-0.371	0.1736	-0.1027	-0.6535	-0.6909	-0.4834	-0.8773	0.5248	-0.7515	-0.7186	0.1001	0.552	0.8791
unclassified	0.247	-0.3966		-0.8138	0.1677	-0.5523	-0.3155	-0.4803	-0.8626	-0.4159	0.43	-0.01442	0.9955	-0.3918	-0.6261	-0.4043	-0.6024	-0.01137	0.931	0.9258	-0.5169	-0.08691	-0.4268	0.954	0.6732	-0.5806	-0.09243	-0.9534	-0.2636	-0.3281	0.5553	-0.5122	-0.6621
Sutterellaceae	-0.09676	0.8473	-0.8138		0.3563	0.9244	0.2697	-0.1138	0.408	0.4063	-0.5289	0.2337	-0.8652	0.4648	0.1407	0.1747	0.7294	-0.4678	-0.9338	-0.7849	0.0005609	0.05325	0.08771	-0.9472	-0.8728	0.002622	-0.492	0.8943	-0.2923	-0.2408	-0.3323	0.5526	0.9578
unclassified	-0.2854	0.6149	0.1677	0.3563		0.5178	-0.5111	-0.7544	-0.5682	0.4828	-0.6159	-0.1434	0.08748	-0.298	-0.4151	-0.7331	0.5919	-0.4107	-0.2032	0.2974	-0.9302	-0.5409	-0.8458	-0.05948	-0.6046	-0.8315	-0.7442	0.1248	-0.5762	-0.6153	-0.1377	-0.3035	0.6032
unclassified	0.1667	0.9842	-0.5523	0.9244	0.5178		0.3484	-0.4648	0.06129	0.1769	-0.3498	0.472	-0.6275	0.567	-0.2473	0.1501	0.5566	-0.7584	-0.7285	-0.6171	-0.23	0.187	-0.000198	-0.7768	-0.7492	-0.3242	-0.779	0.6565	-0.635	-0.5919	-0.006076	0.6096	0.9212
Bdellovibrionaceae	0.8294	0.3036	-0.3155	0.2697	-0.5111	0.3484		0.0145	0.2509	-0.7218	0.6626	0.8897	-0.3178	0.9689	-0.2466	0.9519	-0.4496	-0.5565	-0.1075	-0.6481	0.5819	0.9714	0.8706	-0.3637	0.2343	0.257	-0.1792	0.09421	-0.3275	-0.2566	0.5938	0.9509	0.03063
unclassified	-0.3925	-0.6141	-0.4803	-0.1138	-0.7544	-0.4648	0.0145		0.8548	0.2103	-0.04423	-0.4393	-0.3956	-0.1395	0.9079	0.3161	0.0168	0.789	-0.2092	-0.3512	0.8191	-0.05562	0.4954	-0.1956	0.1021	0.9688	0.9163	0.3086	0.9349	0.9608	-0.5453	-0.05929	-0.2825
Desulfovibrionaceae	-0.3138	-0.1162	-0.8626	0.408	-0.5682	0.06129	0.2509	0.8548		0.3092	-0.224	-0.1904	-0.8108	0.2018	0.8657	0.4738	0.3187	0.431	-0.6519	-0.7673	0.8067	0.07974	0.5859	-0.6736	-0.303	0.9087	0.5755	0.7217	0.6727	0.7287	-0.5915	0.3151	0.2087
unclassified	-0.9406	0.1197	-0.4159	0.4063	0.4828	0.1769	-0.7218	0.2103	0.3092		-0.9802	-0.7829	-0.4235	-0.606	0.5951	-0.6522	0.9154	0.424	-0.606	-0.05402	-0.2829	-0.8654	-0.5838	-0.3777	-0.7817	0.05974	0.1019	0.621	0.3797	0.3583	-0.9337	-0.5051	0.5443
Helicobacteraceae	0.8591	-0.3069	0.43	-0.5289	-0.6159	-0.3498	0.6626	-0.04423	-0.224	-0.9802		0.6604	0.4557	0.5054	-0.4583	0.6507	-0.9662	-0.2366	0.6663	0.09733	0.3895	0.817	0.6214	0.4505	0.8724	0.08166	0.09407	-0.6615	-0.1946	-0.1765	0.8592	0.4092	-0.6806
unclassified	0.9449	0.5047	-0.01442	0.2337	-0.1434	0.472	0.8897	-0.4393	-0.1904	-0.7829	0.6604		-0.05215	0.9195	-0.6521	0.7133	-0.4662	-0.841	0.0588	-0.3797	0.155	0.9099	0.5622	-0.1807	0.224	-0.2108	-0.5532	-0.1141	-0.7103	-0.6607	0.8152	0.8603	0.09554
Anaeroplasmataceae	0.2273	-0.4806	0.9955	-0.8652	0.08748	-0.6275	-0.3178	-0.3956	-0.8108	-0.4235	0.4557	-0.05215		-0.4154	-0.5623	-0.3788	-0.6375	0.06743	0.9552	0.9274	-0.4469	-0.08541	-0.3839	0.9777	0.7223	-0.5015	0.0007948	-0.968	-0.179	-0.2432	0.5319	-0.5335	-0.7271
Verrucomicrobiaceae	0.7911	0.5298	-0.3918	0.4648	-0.298	0.567	0.9689	-0.1395	0.2018	-0.606	0.5054	0.9195	-0.4154		-0.3151	0.8665	-0.266	-0.707	-0.2608	-0.7075	0.4269	0.9079	0.7511	-0.5	0.02424	0.1103	-0.3829	0.2277	-0.4806	-0.4085	0.5473	0.9909	0.2591
Lactobacillaceae	-0.7001	-0.4002	-0.6261	0.1407	-0.4151	-0.2473	-0.2466	0.9079	0.8657	0.5951	-0.4583	-0.6521	-0.5623	-0.3151		0.02487	0.426	0.7868	-0.4827	-0.3757	0.5746	-0.3778	0.1943	-0.3839	-0.286	0.8325	0.7664	0.5685	0.9053	0.9226	-0.8434	-0.2021	0.04995
Streptococcaceae	0.686	0.06681	-0.4043	0.1747	-0.7331	0.1501	0.9519	0.3161	0.4738	-0.6522	0.6507	0.7133	-0.3788	0.8665	0.02487		-0.4643	-0.2773	-0.1179	-0.6873	0.8022	0.9147	0.9794	-0.3595	0.2999	0.5341	0.1244	0.1366	-0.02284	0.0504	0.4261	0.8685	-0.103
unclassified	-0.7263	0.4962	-0.6024	0.7294	0.5919	0.5566	-0.4496	0.0168	0.3187	0.9154	-0.9662	-0.4662	-0.6375	-0.266	0.426	-0.4643		0.05961	-0.8247	-0.3242	-0.2997	-0.6411	-0.4645	-0.6552	-0.9666	-0.04923	-0.2115	0.8104	0.0819	0.08359	-0.8009	-0.1606	0.8356
Clostridiaceae_1	-0.6939	-0.8318	-0.01137	-0.4678	-0.4107	-0.7584	-0.5565	0.789	0.431	0.424	-0.2366	-0.841	0.06743	-0.707	0.7868	-0.2773	0.05961		0.122	0.2476	0.3375	-0.5429	-0.07746	0.2642	0.1749	0.6149	0.9146	-0.02928	0.9543	0.926	-0.6354	-0.6579	-0.4672
Eubacteriaceae	0.3693	-0.6088	0.931	-0.9338	-0.2032	-0.7285	-0.1075	-0.2092	-0.6519	-0.606	0.6663	0.0588	0.9552	-0.2608	-0.4827	-0.1179	-0.8247	0.122		0.8023	-0.1689	0.1308	-0.102	0.9652	0.8946	-0.2757	0.1698	-0.9946	-0.06492	-0.1136	0.6185	-0.3796	-0.8766
Lachnospiraceae	-0.1372	-0.4786	0.9258	-0.7849	0.2974	-0.6171	-0.6481	-0.3512	-0.7673	-0.05402	0.09733	-0.3797	0.9274	-0.7075	-0.3757	-0.6873	-0.3242	0.2476	0.8023		-0.6109	-0.4513	-0.665	0.9213	0.4725	-0.5299	0.04093	-0.8105	-0.04246	-0.1221	0.2015	-0.7961	-0.5755
Ruminococcaceae	0.1792	-0.371	-0.5169	0.0005609	-0.9302	-0.23	0.5819	0.8191	0.8067	-0.2829	0.3895	0.155	-0.4469	0.4269	0.5746	0.8022	-0.2997	0.3375	-0.1689	-0.6109		0.5232	0.9041	-0.3078	0.2781	0.9289	0.6632	0.2436	0.5776	0.6367	-0.06638	0.4749	-0.2728
unclassified	0.9214	0.1736	-0.08691	0.05325	-0.5409	0.187	0.9714	-0.05562	0.07974	-0.8654	0.817	0.9099	-0.08541	0.9079	-0.3778	0.9147	-0.6411	-0.5429	0.1308	-0.4513	0.5232		0.8335	-0.133	0.4402	0.1712	-0.1584	-0.1449	-0.3615	-0.3027	0.7479	0.8605	-0.1681
unclassified	0.5632	-0.1027	-0.4268	0.08771	-0.8458	-0.000198	0.8706	0.4954	0.5859	-0.5838	0.6214	0.5622	-0.3839	0.7511	0.1943	0.9794	-0.4645	-0.07746	-0.102	-0.665	0.9041	0.8335		-0.3224	0.343	0.6851	0.3209	0.1409	0.1778	0.2481	0.3047	0.7641	-0.2005
Erysipelotrichaceae	0.1266	-0.6535	0.954	-0.9472	-0.05948	-0.7768	-0.3637	-0.1956	-0.6736	-0.3777	0.4505	-0.1807	0.9777	-0.5	-0.3839	-0.3595	-0.6552	0.2642	0.9652	0.9213	-0.3078	-0.133	-0.3224		0.7779	-0.322	0.2103	-0.9562	0.02929	-0.03494	0.4216	-0.6062	-0.8314
unclassified	0.5268	-0.6909	0.6732	-0.8728	-0.6046	-0.7492	0.2343	0.1021	-0.303	-0.7817	0.8724	0.224	0.7223	0.02424	-0.286	0.2999	-0.9666	0.1749	0.8946	0.4725	0.2781	0.4402	0.343	0.7779		0.1081	0.3883	-0.8634	0.1106	0.09392	0.6487	-0.07545	-0.9479
TM7_family_incertae_sedis	-0.1962	-0.4834	-0.5806	0.002622	-0.8315	-0.3242	0.257	0.9688	0.9087	0.05974	0.08166	-0.2108	-0.5015	0.1103	0.8325	0.5341	-0.04923	0.6149	-0.2757	-0.5299	0.9289	0.1712	0.6851	-0.322	0.1081		0.8237	0.3673	0.8181	0.8621	-0.4107	0.1886	-0.2182
unclassified	-0.3852	-0.8773	-0.09243	-0.492	-0.7442	-0.779	-0.1792	0.9163	0.5755	0.1019	0.09407	-0.5532	0.0007948	-0.3829	0.7664	0.1244	-0.2115	0.9146	0.1698	0.04093	0.6632	-0.1584	0.3209	0.2103	0.3883	0.8237		-0.06693	0.9567	0.9538	-0.4107	-0.3432	-0.6021
"Porphyromonadaceae"	-0.4109	0.5248	-0.9534	0.8943	0.1248	0.6565	0.09421	0.3086	0.7217	0.621	-0.6615	-0.1141	-0.968	0.2277	0.5685	0.1366	0.8104	-0.02928	-0.9946	-0.8105	0.2436	-0.1449	0.1409	-0.9562	-0.8634	0.3673	-0.06693		0.1654	0.2147	-0.6668	0.352	0.8237
"Prevotellaceae"	-0.6142	-0.7515	-0.2636	-0.2923	-0.5762	-0.635	-0.3275	0.9349	0.6727	0.3797	-0.1946	-0.7103	-0.179	-0.4806	0.9053	-0.02284	0.0819	0.9543	-0.06492	-0.04246	0.5776	-0.3615	0.1778	0.02929	0.1106	0.8181	0.9567	0.1654		0.9965	-0.6581	-0.4095	-0.3697
"Rikenellaceae"	-0.5797	-0.7186	-0.3281	-0.2408	-0.6153	-0.5919	-0.2566	0.9608	0.7287	0.3583	-0.1765	-0.6607	-0.2432	-0.4085	0.9226	0.0504	0.08359	0.926	-0.1136	-0.1221	0.6367	-0.3027	0.2481	-0.03494	0.09392	0.8621	0.9538	0.2147	0.9965		-0.6513	-0.3327	-0.3391
Bacteroidaceae	0.9414	0.1001	0.5553	-0.3323	-0.1377	-0.006076	0.5938	-0.5453	-0.5915	-0.9337	0.8592	0.8152	0.5319	0.5473	-0.8434	0.4261	-0.8009	-0.6354	0.6185	0.2015	-0.06638	0.7479	0.3047	0.4216	0.6487	-0.4107	-0.4107	-0.6668	-0.6581	-0.6513		0.4296	-0.3844
unclassified	0.7024	0.552	-0.5122	0.5526	-0.3035	0.6096	0.9509	-0.05929	0.3151	-0.5051	0.4092	0.8603	-0.5335	0.9909	-0.2021	0.8685	-0.1606	-0.6579	-0.3796	-0.7961	0.4749	0.8605	0.7641	-0.6062	-0.07545	0.1886	-0.3432	0.352	-0.4095	-0.3327	0.4296		0.3382
unclassified	-0.2293	0.8791	-0.6621	0.9578	0.6032	0.9212	0.03063	-0.2825	0.2087	0.5443	-0.6806	0.09554	-0.7271	0.2591	0.04995	-0.103	0.8356	-0.4672	-0.8766	-0.5755	-0.2728	-0.1681	-0.2005	-0.8314	-0.9479	-0.2182	-0.6021	0.8237	-0.3697	-0.3391	-0.3844	0.3382	

Ahr -/+
Family v Family Pearson correlation p

	Deferribacteraceae	Coriobacteriaceae	unclassified	Sutterellaceae	unclassified	unclassified	Bdellovibrionaceae	unclassified	Desulfovibrionaceae	unclassified	Helicobacteraceae	unclassified	Anaeroplasmataceae	Verrucomicrobiaceae	Lactobacillaceae	Streptococcaceae	unclassified	Clostridiaceae_1	Eubacteriaceae	Lachnospiraceae	Ruminococcaceae	unclassified	unclassified	Erysipelotrichaceae	unclassified	TM7_family_incertae_sedis	unclassified	"Porphyromonadaceae"	"Prevotellaceae"	"Rikenellaceae"	Bacteroidaceae	unclassified	unclassified
Deferribacteraceae		0.777	0.753	0.9032	0.7146	0.8333	0.1706	0.6075	0.6862	0.0594	0.1409	0.0551	0.7727	0.2089	0.2999	0.314	0.2737	0.3061	0.6307	0.8628	0.8208	0.0786	0.4368	0.8734	0.4732	0.8038	0.6148	0.5891	0.3858	0.4203	0.0586	0.2976	0.7707
Coriobacteriaceae	0.777		0.6034	0.1527	0.3851	0.0158	0.6964	0.3859	0.8838	0.8803	0.6931	0.4953	0.5194	0.4702	0.5998	0.9332	0.5038	0.1682	0.3912	0.5214	0.629	0.8264	0.8973	0.3465	0.3091	0.5166	0.1227	0.4752	0.2485	0.2814	0.8999	0.448	0.1209
unclassified	0.753	0.6034		0.1862	0.8323	0.4477	0.6845	0.5197	0.1374	0.5841	0.57	0.9856	0.0045	0.6082	0.3739	0.5957	0.3976	0.9886	0.069	0.0742	0.4831	0.9131	0.5732	0.046	0.3268	0.4194	0.9076	0.0466	0.7364	0.6719	0.4447	0.4878	0.3379
Sutterellaceae	0.9032	0.1527	0.1862		0.6437	0.0756	0.7303	0.8862	0.592	0.5937	0.4711	0.7663	0.1348	0.5352	0.8593	0.8253	0.2706	0.5322	0.0662	0.2151	0.9994	0.9467	0.9123	0.0528	0.1272	0.9974	0.508	0.1057	0.7077	0.7592	0.6677	0.4474	0.0422
unclassified	0.7146	0.3851	0.8323	0.6437		0.4822	0.4889	0.2456	0.4318	0.5172	0.3841	0.8566	0.9125	0.702	0.5849	0.2669	0.4081	0.5893	0.7968	0.7026	0.0698	0.4591	0.1542	0.9405	0.3954	0.1685	0.2558	0.8752	0.4238	0.3847	0.8623	0.6965	0.3968
unclassified	0.8333	0.0158	0.4477	0.0756	0.4822		0.6516	0.5352	0.9387	0.8231	0.6502	0.528	0.3725	0.433	0.7527	0.8499	0.4434	0.2416	0.2715	0.3829	0.77	0.813	0.9998	0.2232	0.2508	0.6758	0.221	0.3435	0.365	0.4081	0.9939	0.3904	0.0788
Bdellovibrionaceae	0.1706	0.6964	0.6845	0.7303	0.4889	0.6516		0.9855	0.7491	0.2782	0.3374	0.1103	0.6822	0.0311	0.7534	0.0481	0.5504	0.4435	0.8925	0.3519	0.4181	0.0286	0.1294	0.6363	0.7657	0.743	0.8208	0.9058	0.6725	0.7434	0.4062	0.0491	0.9694
unclassified	0.6075	0.3859	0.5197	0.8862	0.2456	0.5352	0.9855		0.1452	0.7897	0.9558	0.5607	0.6044	0.8605	0.0921	0.6839	0.9832	0.211	0.7908	0.6488	0.1809	0.9444	0.5046	0.8044	0.8979	0.0312	0.0837	0.6914	0.0651	0.0392	0.4547	0.9407	0.7175
Desulfovibrionaceae	0.6862	0.8838	0.1374	0.592	0.4318	0.9387	0.7491	0.1452		0.6908	0.776	0.8096	0.1892	0.7982	0.1343	0.5262	0.6813	0.569	0.3481	0.2327	0.1933	0.9203	0.4141	0.3264	0.697	0.0913	0.4245	0.2783	0.3273	0.2713	0.4085	0.6849	0.7913
unclassified	0.0594	0.8803	0.5841	0.5937	0.5172	0.8231	0.2782	0.7897	0.6908		0.0198	0.2171	0.5765	0.394	0.4049	0.3478	0.0846	0.576	0.394	0.946	0.7171	0.1346	0.4162	0.6223	0.2183	0.9403	0.8981	0.379	0.6203	0.6417	0.0663	0.4949	0.4557
Helicobacteraceae	0.1409	0.6931	0.57	0.4711	0.3841	0.6502	0.3374	0.9558	0.776	0.0198		0.3396	0.5443	0.4946	0.5417	0.3493	0.0338	0.7634	0.3337	0.9027	0.6105	0.183	0.3786	0.5495	0.1276	0.9183	0.9059	0.3385	0.8054	0.8235	0.1408	0.5908	0.3194
unclassified	0.0551	0.4953	0.9856	0.7663	0.8566	0.528	0.1103	0.5607	0.8096	0.2171	0.3396		0.9478	0.0805	0.3479	0.2867	0.5338	0.159	0.9412	0.6203	0.845	0.0901	0.4378	0.8193	0.776	0.7892	0.4468	0.8859	0.2897	0.3393	0.1848	0.1397	0.9045
Anaeroplasmataceae	0.7727	0.5194	0.0045	0.1348	0.9125	0.3725	0.6822	0.6044	0.1892	0.5765	0.5443	0.9478		0.5846	0.4377	0.6212	0.3625	0.9326	0.0448	0.0726	0.5531	0.9146	0.6161	0.0223	0.2777	0.4985	0.9992	0.032	0.821	0.7568	0.4681	0.4665	0.2729
Verrucomicrobiaceae	0.2089	0.4702	0.6082	0.5352	0.702	0.433	0.0311	0.8605	0.7982	0.394	0.4946	0.0805	0.5846		0.6849	0.1335	0.734	0.293	0.7392	0.2925	0.5731	0.0921	0.2489	0.5	0.9758	0.8897	0.6171	0.7723	0.5194	0.5915	0.4527	0.0091	0.7409
Lactobacillaceae	0.2999	0.5998	0.3739	0.8593	0.5849	0.7527	0.7534	0.0921	0.1343	0.4049	0.5417	0.3479	0.4377	0.6849		0.9751	0.574	0.2132	0.5173	0.6243	0.4254	0.6222	0.8057	0.6161	0.714	0.1675	0.2336	0.4315	0.0947	0.0774	0.1566	0.7979	0.95
Streptococcaceae	0.314	0.9332	0.5957	0.8253	0.2669	0.8499	0.0481	0.6839	0.5262	0.3478	0.3493	0.2867	0.6212	0.1335	0.9751		0.5357	0.7227	0.8821	0.3127	0.1978	0.0853	0.0206	0.6405	0.7001	0.4659	0.8756	0.8634	0.9772	0.9496	0.5739	0.1315	0.897
unclassified	0.2737	0.5038	0.3976	0.2706	0.4081	0.4434	0.5504	0.9832	0.6813	0.0846	0.0338	0.5338	0.3625	0.734	0.574	0.5357		0.9404	0.1753	0.6758	0.7003	0.3589	0.5355	0.3448	0.0334	0.9508	0.7885	0.1896	0.9181	0.9164	0.1991	0.8394	0.1644
Clostridiaceae_1	0.3061	0.1682	0.9886	0.5322	0.5893	0.2416	0.4435	0.211	0.569	0.576	0.7634	0.159	0.9326	0.293	0.2132	0.7227	0.9404		0.878	0.7524	0.6625	0.4571	0.9225	0.7358	0.8251	0.3851	0.0854	0.9707	0.0457	0.074	0.3646	0.3421	0.5328
Eubacteriaceae	0.6307	0.3912	0.069	0.0662	0.7968	0.2715	0.8925	0.7908	0.3481	0.394	0.3337	0.9412	0.0448	0.7392	0.5173	0.8821	0.1753	0.878		0.1977	0.8311	0.8692	0.898	0.0348	0.1054	0.7243	0.8302	0.0054	0.9351	0.8864	0.3815	0.6204	0.1234
Lachnospiraceae	0.8628	0.5214	0.0742	0.2151	0.7026	0.3829	0.3519	0.6488	0.2327	0.946	0.9027	0.6203	0.0726	0.2925	0.6243	0.3127	0.6758	0.7524	0.1977		0.3891	0.5487	0.335	0.0787	0.5275	0.4701	0.9591	0.1895	0.9575	0.8779	0.7985	0.2039	0.4245
Ruminococcaceae	0.8208	0.629	0.4831	0.9994	0.0698	0.77	0.4181	0.1809	0.1933	0.7171	0.6105	0.845	0.5531	0.5731	0.4254	0.1978	0.7003	0.6625	0.8311	0.3891		0.4768	0.0959	0.6922	0.7219	0.0711	0.3368	0.7564	0.4224	0.3633	0.9336	0.5251	0.7272
unclassified	0.0786	0.8264	0.9131	0.9467	0.4591	0.813	0.0286	0.9444	0.9203	0.1346	0.183	0.0901	0.9146	0.0921	0.6222	0.0853	0.3589	0.4571	0.8692	0.5487	0.4768		0.1665	0.867	0.5598	0.8288	0.8416	0.8551	0.6385	0.6973	0.2521	0.1395	0.8319
unclassified	0.4368	0.8973	0.5732	0.9123	0.1542	0.9998	0.1294	0.5046	0.4141	0.4162	0.3786	0.4378	0.6161	0.2489	0.8057	0.0206	0.5355	0.9225	0.898	0.335	0.0959	0.1665		0.6776	0.657	0.3149	0.6791	0.8591	0.8222	0.7519	0.6953	0.2359	0.7995
Erysipelotrichaceae	0.8734	0.3465	0.046	0.0528	0.9405	0.2232	0.6363	0.8044	0.3264	0.6223	0.5495	0.8193	0.0223	0.5	0.6161	0.6405	0.3448	0.7358	0.0348	0.0787	0.6922	0.867	0.6776		0.2221	0.678	0.7897	0.0438	0.9707	0.9651	0.5784	0.3938	0.1686
unclassified	0.4732	0.3091	0.3268	0.1272	0.3954	0.2508	0.7657	0.8979	0.697	0.2183	0.1276	0.776	0.2777	0.9758	0.714	0.7001	0.0334	0.8251	0.1054	0.5275	0.7219	0.5598	0.657	0.2221		0.8919	0.6117	0.1366	0.8894	0.9061	0.3513	0.9245	0.0521
TM7_family_incertae_sedis	0.8038	0.5166	0.4194	0.9974	0.1685	0.6758	0.743	0.0312	0.0913	0.9403	0.9183	0.7892	0.4985	0.8897	0.1675	0.4659	0.9508	0.3851	0.7243	0.4701	0.0711	0.8288	0.3149	0.678	0.8919		0.1763	0.6327	0.1819	0.1379	0.5893	0.8114	0.7818
unclassified	0.6148	0.1227	0.9076	0.508	0.2558	0.221	0.8208	0.0837	0.4245	0.8981	0.9059	0.4468	0.9992	0.6171	0.2336	0.8756	0.7885	0.0854	0.8302	0.9591	0.3368	0.8416	0.6791	0.7897	0.6117	0.1763		0.9331	0.0433	0.0462	0.5893	0.6568	0.3979
"Porphyromonadaceae"	0.5891	0.4752	0.0466	0.1057	0.8752	0.3435	0.9058	0.6914	0.2783	0.379	0.3385	0.8859	0.032	0.7723	0.4315	0.8634	0.1896	0.9707	0.0054	0.1895	0.7564	0.8551	0.8591	0.0438	0.1366	0.6327	0.9331		0.8346	0.7853	0.3332	0.648	0.1763
"Prevotellaceae"	0.3858	0.2485	0.7364	0.7077	0.4238	0.365	0.6725	0.0651	0.3273	0.6203	0.8054	0.2897	0.821	0.5194	0.0947	0.9772	0.9181	0.0457	0.9351	0.9575	0.4224	0.6385	0.8222	0.9707	0.8894	0.1819	0.0433	0.8346		0.0035	0.3419	0.5905	0.6303
"Rikenellaceae"	0.4203	0.2814	0.6719	0.7592	0.3847	0.4081	0.7434	0.0392	0.2713	0.6417	0.8235	0.3393	0.7568	0.5915	0.0774	0.9496	0.9164	0.074	0.8864	0.8779	0.3633	0.6973	0.7519	0.9651	0.9061	0.1379	0.0462	0.7853	0.0035		0.3487	0.6673	0.6609
Bacteroidaceae	0.0586	0.8999	0.4447	0.6677	0.8623	0.9939	0.4062	0.4547	0.4085	0.0663	0.1408	0.1848	0.4681	0.4527	0.1566	0.5739	0.1991	0.3646	0.3815	0.7985	0.9336	0.2521	0.6953	0.5784	0.3513	0.5893	0.5893	0.3332	0.3419	0.3487		0.5704	0.6156
unclassified	0.2976	0.448	0.4878	0.4474	0.6965	0.3904	0.0491	0.9407	0.6849	0.4949	0.5908	0.1397	0.4665	0.0091	0.7979	0.1315	0.8394	0.3421	0.6204	0.2039	0.5251	0.1395	0.2359	0.3938	0.9245	0.8114	0.6568	0.648	0.5905	0.6673	0.5704		0.6618
unclassified	0.7707	0.1209	0.3379	0.0422	0.3968	0.0788	0.9694	0.7175	0.7913	0.4557	0.3194	0.9045	0.2729	0.7409	0.95	0.897	0.1644	0.5328	0.1234	0.4245	0.7272	0.8319	0.7995	0.1686	0.0521	0.7818	0.3979	0.1763	0.6303	0.6609	0.6156	0.6618	

Ahr -/-
Family v Family Pearson correlation r

	Deferribacteraceae	Coriobacteriaceae	unclassified	Sutterellaceae	unclassified	unclassified	Bdellovibrionaceae	unclassified	Desulfovibrionaceae	unclassified	Helicobacteraceae	unclassified	Anaeroplasmataceae	Verrucomicrobiaceae	Lactobacillaceae	Streptococcaceae	unclassified	Clostridiaceae_1	Eubacteriaceae	Lachnospiraceae	Ruminococcaceae	unclassified	unclassified	Erysipelotrichaceae	unclassified	TM7_family_incertae_sedis	unclassified	"Porphyromonadaceae"	"Prevotellaceae"	"Rikenellaceae"	Bacteroidaceae	unclassified	unclassified
Deferribacteraceae		-0.5694	-0.3167	0.4296	-0.6329	0.005748	-0.1997	0.9905	0.1465	0.6071	0.2252	-0.1963	-0.9362	0.1845	0.5575	0.2673	-0.1809	0.1279	-0.3074	0.1881	0.6914	-0.9796	0.8701	-0.1372	0.4121	0.2668	-0.3158	-0.04332	-0.9135	0.2313	0.4338	-0.9052	0.4333
Coriobacteriaceae	-0.5694		0.5984	0.4926	-0.212	-0.0301	-0.6914	-0.602	-0.7747	0.2306	0.5799	0.5528	0.287	0.3279	-0.4907	0.6155	-0.6408	-0.817	-0.3698	-0.7017	-0.783	0.7212	-0.766	0.8884	-0.4324	-0.8862	0.8182	0.6629	0.2213	-0.8598	0.01165	0.1754	-0.7156
unclassified	-0.3167	0.5984		0.2354	0.1173	0.7818	-0.4132	-0.2334	-0.8911	0.4637	0.002247	-0.3222	0.2919	0.8738	-0.9267	0.2003	-0.7115	-0.8253	-0.7864	-0.9594	-0.8967	0.4344	-0.7367	0.6233	-0.9565	-0.2491	0.9314	0.9119	0.2509	-0.8459	0.6986	0.1547	-0.986
Sutterellaceae	0.4296	0.4926	0.2354		-0.9321	-0.1324	-0.9671	0.3704	-0.6362	0.8501	0.9174	0.4834	-0.6901	0.464	0.1353	0.9775	-0.8485	-0.7141	-0.6544	-0.4977	-0.07001	-0.2457	0.1174	0.8147	0.05419	-0.7247	0.5033	0.6113	-0.7378	-0.6525	0.3794	-0.7702	-0.2517
unclassified	-0.6329	-0.212	0.1173	-0.9321		0.3574	0.8176	-0.5508	0.3166	-0.7459	-0.8964	-0.5271	0.8633	-0.2028	-0.4779	-0.8998	0.6141	0.4123	0.425	0.1598	-0.2925	0.4914	-0.4456	-0.5663	-0.3908	0.5756	-0.1565	-0.3005	0.8932	0.3342	-0.2079	0.894	-0.1132
unclassified	0.005748	-0.0301	0.7818	-0.1324	0.3574		0.0579	0.1367	-0.4923	0.3544	-0.4818	-0.839	0.1887	0.8132	-0.7924	-0.2661	-0.3568	-0.3747	-0.6625	-0.6394	-0.5259	0.01662	-0.351	0.0651	-0.8681	0.3922	0.5176	0.6028	0.1894	-0.372	0.836	0.1061	-0.6763
Bdellovibrionaceae	-0.1997	-0.6914	-0.4132	-0.9671	0.8176	0.0579		-0.1491	0.7803	-0.7978	-0.901	-0.5085	0.4852	-0.5303	0.07333	-0.9746	0.9119	0.8501	0.6921	0.6529	0.311	0.001816	0.1387	-0.9354	0.1295	0.8315	-0.6829	-0.7348	0.5435	0.8105	-0.372	0.5886	0.4532
unclassified	0.9905	-0.602	-0.2334	0.3704	-0.5508	0.1367	-0.1491		0.1164	0.6174	0.1242	-0.3256	-0.8895	0.266	0.4579	0.1909	-0.1873	0.116	-0.3623	0.1336	0.636	-0.9758	0.8295	-0.1687	0.3054	0.3524	-0.278	0.00272	-0.8643	0.2178	0.5246	-0.8648	0.365
Desulfovibrionaceae	0.1465	-0.7747	-0.8911	-0.6362	0.3166	-0.4923	0.7803	0.1164		-0.6955	-0.4543	-0.05236	0.01856	-0.8482	0.6772	-0.6227	0.9352	0.9911	0.868	0.9819	0.7837	-0.3256	0.5954	-0.9001	0.7204	0.6019	-0.9833	-0.9868	0.07667	0.9884	-0.6409	0.1643	0.9066
unclassified	0.6071	0.2306	0.4637	0.8501	-0.7459	0.3544	-0.7978	0.6174	-0.6955		0.5704	-0.05006	-0.7092	0.7917	-0.1096	0.7284	-0.8884	-0.7086	-0.9067	-0.6414	-0.1163	-0.4447	0.1602	0.6445	-0.2471	-0.329	0.5567	0.7532	-0.741	-0.6311	0.7997	-0.803	-0.3979
Helicobacteraceae	0.2252	0.5799	0.002247	0.9174	-0.8964	-0.4818	-0.901	0.1242	-0.4543	0.5704		0.7916	-0.5526	0.1176	0.3095	0.9727	-0.6447	-0.5665	-0.3294	-0.2782	-0.003834	-0.06251	0.0794	0.7735	0.2857	-0.8741	0.3498	0.3729	-0.6021	-0.5277	-0.01561	-0.6047	-0.07609
unclassified	-0.1963	0.5528	-0.3222	0.4834	-0.5271	-0.839	-0.5085	-0.3256	-0.05236	-0.05006	0.7916		-0.1281	-0.4347	0.4365	0.6433	-0.1335	-0.1782	0.2652	0.1204	0.05807	0.2753	-0.04627	0.4752	0.5113	-0.8285	0.03126	-0.09097	-0.1658	-0.1902	-0.6093	-0.1245	0.1817
Anaeroplasmataceae	-0.9362	0.287	0.2919	-0.6901	0.8633	0.1887	0.4852	-0.8895	0.01856	-0.7092	-0.5526	-0.1281		-0.1774	-0.6069	-0.5736	0.3691	0.07654	0.3603	-0.08207	-0.6061	0.8629	-0.7837	-0.1485	-0.4759	0.08436	0.1636	-0.07594	0.9977	-0.02558	-0.3446	0.9892	-0.3635
Verrucomicrobiaceae	0.1845	0.3279	0.8738	0.464	-0.2028	0.8132	-0.5303	0.266	-0.8482	0.7917	0.1176	-0.4347	-0.1774		-0.6744	0.3444	-0.8298	-0.7894	-0.9724	-0.8976	-0.5746	-0.05227	-0.3173	0.5753	-0.7798	-0.1212	0.8031	0.9227	-0.2083	-0.7578	0.9463	-0.3038	-0.7995
Lactobacillaceae	0.5575	-0.4907	-0.9267	0.1353	-0.4779	-0.7924	0.07333	0.4579	0.6772	-0.1096	0.3095	0.4365	-0.6069	-0.6744		0.1427	0.3994	0.5874	0.5166	0.7908	0.9234	-0.6111	0.8491	-0.363	0.9861	0.04653	-0.7774	-0.6926	-0.5795	0.6388	-0.5024	-0.496	0.9221
Streptococcaceae	0.2673	0.6155	0.2003	0.9775	-0.8998	-0.2661	-0.9746	0.1909	-0.6227	0.7284	0.9727	0.6433	-0.5736	0.3444	0.1427		-0.8019	-0.7151	-0.5392	-0.4676	-0.128	-0.08315	0.01224	0.8606	0.09406	-0.8488	0.5116	0.5644	-0.6271	-0.6699	0.2095	-0.6515	-0.2513
unclassified	-0.1809	-0.6408	-0.7115	-0.8485	0.6141	-0.3568	0.9119	-0.1873	0.9352	-0.8884	-0.6447	-0.1335	0.3691	-0.8298	0.3994	-0.8019		0.9521	0.9185	0.8766	0.5137	-0.01367	0.2819	-0.9049	0.4818	0.6206	-0.8557	-0.937	0.4237	0.9147	-0.6886	0.5003	0.7067
Clostridiaceae_1	0.1279	-0.817	-0.8253	-0.7141	0.4123	-0.3747	0.8501	0.116	0.9911	-0.7086	-0.5665	-0.1782	0.07654	-0.7894	0.5874	-0.7151	0.9521		0.8374	0.948	0.7382	-0.3168	0.5579	-0.9496	0.6265	0.6962	-0.9643	-0.9632	0.1382	0.9945	-0.5715	0.2191	0.8545
Eubacteriaceae	-0.3074	-0.3698	-0.7864	-0.6544	0.425	-0.6625	0.6921	-0.3623	0.868	-0.9067	-0.3294	0.2652	0.3603	-0.9724	0.5166	-0.5392	0.9185	0.8374		0.8753	0.4739	0.1498	0.2012	-0.6781	0.6327	0.2631	-0.7899	-0.9306	0.3969	0.7908	-0.9184	0.4856	0.7213
Lachnospiraceae	0.1881	-0.7017	-0.9594	-0.4977	0.1598	-0.6394	0.6529	0.1336	0.9819	-0.6414	-0.2782	0.1204	-0.08207	-0.8976	0.7908	-0.4676	0.8766	0.948	0.8753		0.8351	-0.3471	0.6455	-0.8039	0.8354	0.4559	-0.9833	-0.9875	-0.03034	0.9516	-0.707	0.06397	0.9577
Ruminococcaceae	0.6914	-0.783	-0.8967	-0.07001	-0.2925	-0.5259	0.311	0.636	0.7837	-0.1163	-0.003834	0.05807	-0.6061	-0.5746	0.9234	-0.128	0.5137	0.7382	0.4739	0.8351		-0.7871	0.9576	-0.6152	0.8784	0.4101	-0.8835	-0.7407	-0.5592	0.7989	-0.3097	-0.4833	0.9504
unclassified	-0.9796	0.7212	0.4344	-0.2457	0.4914	0.01662	0.001816	-0.9758	-0.3256	-0.4447	-0.06251	0.2753	0.8629	-0.05227	-0.6111	-0.08315	-0.01367	-0.3168	0.1498	-0.3471	-0.7871		-0.9283	0.3331	-0.4768	-0.4284	0.4793	0.2148	0.8279	-0.4141	-0.3382	0.8074	-0.5578
unclassified	0.8701	-0.766	-0.7367	0.1174	-0.4456	-0.351	0.1387	0.8295	0.5954	0.1602	0.0794	-0.04627	-0.7837	-0.3173	0.8491	0.01224	0.2819	0.5579	0.2012	0.6455	0.9576	-0.9283		-0.4798	0.7602	0.3934	-0.7304	-0.5239	-0.7422	0.6404	-0.03589	-0.6873	0.8207
Erysipelotrichaceae	-0.1372	0.8884	0.6233	0.8147	-0.5663	0.0651	-0.9354	-0.1687	-0.9001	0.6445	0.7735	0.4752	-0.1485	0.5753	-0.363	0.8606	-0.9049	-0.9496	-0.6781	-0.8039	-0.6152	0.3331	-0.4798		-0.3798	-0.8816	0.8623	0.8329	-0.2151	-0.9443	0.3335	-0.2739	-0.6901
unclassified	0.4121	-0.4324	-0.9565	0.05419	-0.3908	-0.8681	0.1295	0.3054	0.7204	-0.2471	0.2857	0.5113	-0.4759	-0.7798	0.9861	0.09406	0.4818	0.6265	0.6327	0.8354	0.8784	-0.4768	0.7602	-0.3798		0.006904	-0.7959	-0.7571	-0.4497	0.662	-0.6388	-0.3595	0.929
TM7_family_incertae_sedis	0.2668	-0.8862	-0.2491	-0.7247	0.5756	0.3922	0.8315	0.3524	0.6019	-0.329	-0.8741	-0.8285	0.08436	-0.1212	0.04653	-0.8488	0.6206	0.6962	0.2631	0.4559	0.4101	-0.4284	0.3934	-0.8816	0.006904		-0.5808	-0.4777	0.1472	0.7073	0.1401	0.1634	0.3727
unclassified	-0.3158	0.8182	0.9314	0.5033	-0.1565	0.5176	-0.6829	-0.278	-0.9833	0.5567	0.3498	0.03126	0.1636	0.8031	-0.7774	0.5116	-0.8557	-0.9643	-0.7899	-0.9833	-0.8835	0.4793	-0.7304	0.8623	-0.7959	-0.5808		0.9594	0.1058	-0.9803	0.5676	0.01785	-0.9602
"Porphyromonadaceae"	-0.04332	0.6629	0.9119	0.6113	-0.3005	0.6028	-0.7348	0.00272	-0.9868	0.7532	0.3729	-0.09097	-0.07594	0.9227	-0.6926	0.5644	-0.937	-0.9632	-0.9306	-0.9875	-0.7407	0.2148	-0.5239	0.8329	-0.7571	-0.4777	0.9594		-0.1277	-0.9508	0.7563	-0.2205	-0.9003
"Prevotellaceae"	-0.9135	0.2213	0.2509	-0.7378	0.8932	0.1894	0.5435	-0.8643	0.07667	-0.741	-0.6021	-0.1658	0.9977	-0.2083	-0.5795	-0.6271	0.4237	0.1382	0.3969	-0.03034	-0.5592	0.8279	-0.7422	-0.2151	-0.4497	0.1472	0.1058	-0.1277		0.03715	-0.3563	0.9951	-0.316
"Rikenellaceae"	0.2313	-0.8598	-0.8459	-0.6525	0.3342	-0.372	0.8105	0.2178	0.9884	-0.6311	-0.5277	-0.1902	-0.02558	-0.7578	0.6388	-0.6699	0.9147	0.9945	0.7908	0.9516	0.7989	-0.4141	0.6404	-0.9443	0.662	0.7073	-0.9803	-0.9508	0.03715		-0.5182	0.1177	0.8863
Bacteroidaceae	0.4338	0.01165	0.6986	0.3794	-0.2079	0.836	-0.372	0.5246	-0.6409	0.7997	-0.01561	-0.6093	-0.3446	0.9463	-0.5024	0.2095	-0.6886	-0.5715	-0.9184	-0.707	-0.3097	-0.3382	-0.03589	0.3335	-0.6388	0.1401	0.5676	0.7563	-0.3563	-0.5182		-0.4403	-0.5807
unclassified	-0.9052	0.1754	0.1547	-0.7702	0.894	0.1061	0.5886	-0.8648	0.1643	-0.803	-0.6047	-0.1245	0.9892	-0.3038	-0.496	-0.6515	0.5003	0.2191	0.4856	0.06397	-0.4833	0.8074	-0.6873	-0.2739	-0.3595	0.1634	0.01785	-0.2205	0.9951	0.1177	-0.4403		-0.2245
unclassified	0.4333	-0.7156	-0.986	-0.2517	-0.1132	-0.6763	0.4532	0.365	0.9066	-0.3979	-0.07609	0.1817	-0.3635	-0.7995	0.9221	-0.2513	0.7067	0.8545	0.7213	0.9577	0.9504	-0.5578	0.8207	-0.6901	0.929	0.3727	-0.9602	-0.9003	-0.316	0.8863	-0.5807	-0.2245	

Ahr -/-
Family v Family Pearson correlation p

	Deferribacteraceae	Coriobacteriaceae	unclassified	Sutterellaceae	unclassified	unclassified	Bdellovibrionaceae	unclassified	Desulfovibrionaceae	unclassified	Helicobacteraceae	unclassified	Anaeroplasmataceae	Verrucomicrobiaceae	Lactobacillaceae	Streptococcaceae	unclassified	Clostridiaceae_1	Eubacteriaceae	Lachnospiraceae	Ruminococcaceae	unclassified	unclassified	Erysipelotrichaceae	unclassified	TM7_family_incertae_sedis	unclassified	"Porphyromonadaceae"	"Prevotellaceae"	"Rikenellaceae"	Bacteroidaceae	unclassified	unclassified
Deferribacteraceae		0.4306	0.6833	0.5704	0.3671	0.9943	0.8003	0.0095	0.8535	0.3929	0.7748	0.8037	0.0638	0.8155	0.4425	0.7327	0.8191	0.8721	0.6926	0.8119	0.3086	0.0204	0.1299	0.8628	0.5879	0.7332	0.6842	0.9567	0.0865	0.7687	0.5662	0.0948	0.5667
Coriobacteriaceae	0.4306		0.4016	0.5074	0.788	0.9699	0.3086	0.398	0.2253	0.7694	0.4201	0.4472	0.713	0.6721	0.5093	0.3845	0.3592	0.183	0.6302	0.2983	0.217	0.2788	0.234	0.1116	0.5676	0.1138	0.1818	0.3371	0.7787	0.1402	0.9884	0.8246	0.2844
unclassified	0.6833	0.4016		0.7646	0.8827	0.2182	0.5868	0.7666	0.1089	0.5363	0.9978	0.6778	0.7081	0.1262	0.0733	0.7997	0.2885	0.1747	0.2136	0.0406	0.1033	0.5656	0.2633	0.3767	0.0435	0.7509	0.0686	0.0881	0.7491	0.1541	0.3014	0.8453	0.014
Sutterellaceae	0.5704	0.5074	0.7646		0.0679	0.8676	0.0329	0.6296	0.3638	0.1499	0.0826	0.5166	0.3099	0.536	0.8647	0.0225	0.1515	0.2859	0.3456	0.5023	0.93	0.7543	0.8826	0.1853	0.9458	0.2753	0.4967	0.3887	0.2622	0.3475	0.6206	0.2298	0.7483
unclassified	0.3671	0.788	0.8827	0.0679		0.6426	0.1824	0.4492	0.6834	0.2541	0.1036	0.4729	0.1367	0.7972	0.5221	0.1002	0.3859	0.5877	0.575	0.8402	0.7075	0.5086	0.5544	0.4337	0.6092	0.4244	0.8435	0.6995	0.1068	0.6658	0.7921	0.106	0.8868
unclassified	0.9943	0.9699	0.2182	0.8676	0.6426		0.9421	0.8633	0.5077	0.6456	0.5182	0.161	0.8113	0.1868	0.2076	0.7339	0.6433	0.6253	0.3375	0.3606	0.4741	0.9834	0.649	0.9349	0.1319	0.6078	0.4824	0.3972	0.8106	0.628	0.164	0.8939	0.3237
Bdellovibrionaceae	0.8003	0.3086	0.5868	0.0329	0.1824	0.9421		0.8509	0.2197	0.2022	0.099	0.4915	0.5148	0.4697	0.9267	0.0254	0.0881	0.1499	0.3079	0.3471	0.689	0.9982	0.8613	0.0646	0.8705	0.1685	0.3171	0.2652	0.4565	0.1895	0.628	0.4114	0.5468
unclassified	0.0095	0.398	0.7666	0.6296	0.4492	0.8633	0.8509		0.8836	0.3826	0.8758	0.6744	0.1105	0.734	0.5421	0.8091	0.8127	0.884	0.6377	0.8664	0.364	0.0242	0.1705	0.8313	0.6946	0.6476	0.722	0.9973	0.1357	0.7822	0.4754	0.1352	0.635
Desulfovibrionaceae	0.8535	0.2253	0.1089	0.3638	0.6834	0.5077	0.2197	0.8836		0.3045	0.5457	0.9476	0.9814	0.1518	0.3228	0.3773	0.0648	0.0089	0.132	0.0181	0.2163	0.6744	0.4046	0.0999	0.2796	0.3981	0.0167	0.0132	0.9233	0.0116	0.3591	0.8357	0.0934
unclassified	0.3929	0.7694	0.5363	0.1499	0.2541	0.6456	0.2022	0.3826	0.3045		0.4296	0.9499	0.2908	0.2083	0.8904	0.2716	0.1116	0.2914	0.0933	0.3586	0.8837	0.5553	0.8398	0.3555	0.7529	0.671	0.4433	0.2468	0.259	0.3689	0.2003	0.197	0.6021
Helicobacteraceae	0.7748	0.4201	0.9978	0.0826	0.1036	0.5182	0.099	0.8758	0.5457	0.4296		0.2084	0.4474	0.8824	0.6905	0.0273	0.3553	0.4335	0.6706	0.7218	0.9962	0.9375	0.9206	0.2265	0.7143	0.1259	0.6502	0.6271	0.3979	0.4723	0.9844	0.3953	0.9239
unclassified	0.8037	0.4472	0.6778	0.5166	0.4729	0.161	0.4915	0.6744	0.9476	0.9499	0.2084		0.8719	0.5653	0.5635	0.3567	0.8665	0.8218	0.7348	0.8796	0.9419	0.7247	0.9537	0.5248	0.4887	0.1715	0.9687	0.909	0.8342	0.8098	0.3907	0.8755	0.8183
Anaeroplasmataceae	0.0638	0.713	0.7081	0.3099	0.1367	0.8113	0.5148	0.1105	0.9814	0.2908	0.4474	0.8719		0.8226	0.3931	0.4264	0.6309	0.9235	0.6397	0.9179	0.3939	0.1371	0.2163	0.8515	0.5241	0.9156	0.8364	0.9241	0.0023	0.9744	0.6554	0.0108	0.6365
Verrucomicrobiaceae	0.8155	0.6721	0.1262	0.536	0.7972	0.1868	0.4697	0.734	0.1518	0.2083	0.8824	0.5653	0.8226		0.3256	0.6556	0.1702	0.2106	0.0276	0.1024	0.4254	0.9477	0.6827	0.4247	0.2202	0.8788	0.1969	0.0773	0.7917	0.2422	0.0537	0.6962	0.2005
Lactobacillaceae	0.4425	0.5093	0.0733	0.8647	0.5221	0.2076	0.9267	0.5421	0.3228	0.8904	0.6905	0.5635	0.3931	0.3256		0.8573	0.6006	0.4126	0.4834	0.2092	0.0766	0.3889	0.1509	0.637	0.0139	0.9535	0.2226	0.3074	0.4205	0.3612	0.4976	0.504	0.0779
Streptococcaceae	0.7327	0.3845	0.7997	0.0225	0.1002	0.7339	0.0254	0.8091	0.3773	0.2716	0.0273	0.3567	0.4264	0.6556	0.8573		0.1981	0.2849	0.4608	0.5324	0.872	0.9168	0.9878	0.1394	0.9059	0.1512	0.4884	0.4356	0.3729	0.3301	0.7905	0.3485	0.7487
unclassified	0.8191	0.3592	0.2885	0.1515	0.3859	0.6433	0.0881	0.8127	0.0648	0.1116	0.3553	0.8665	0.6309	0.1702	0.6006	0.1981		0.0479	0.0815	0.1234	0.4863	0.9863	0.7181	0.0951	0.5182	0.3794	0.1443	0.063	0.5763	0.0853	0.3114	0.4997	0.2933
Clostridiaceae_1	0.8721	0.183	0.1747	0.2859	0.5877	0.6253	0.1499	0.884	0.0089	0.2914	0.4335	0.8218	0.9235	0.2106	0.4126	0.2849	0.0479		0.1626	0.052	0.2618	0.6832	0.4421	0.0504	0.3735	0.3038	0.0357	0.0368	0.8618	0.0055	0.4285	0.7809	0.1455
Eubacteriaceae	0.6926	0.6302	0.2136	0.3456	0.575	0.3375	0.3079	0.6377	0.132	0.0933	0.6706	0.7348	0.6397	0.0276	0.4834	0.4608	0.0815	0.1626		0.1247	0.5261	0.8502	0.7988	0.3219	0.3673	0.7369	0.2101	0.0694	0.6031	0.2092	0.0816	0.5144	0.2787
Lachnospiraceae	0.8119	0.2983	0.0406	0.5023	0.8402	0.3606	0.3471	0.8664	0.0181	0.3586	0.7218	0.8796	0.9179	0.1024	0.2092	0.5324	0.1234	0.052	0.1247		0.1649	0.6529	0.3545	0.1961	0.1646	0.5441	0.0167	0.0125	0.9697	0.0484	0.293	0.936	0.0423
Ruminococcaceae	0.3086	0.217	0.1033	0.93	0.7075	0.4741	0.689	0.364	0.2163	0.8837	0.9962	0.9419	0.3939	0.4254	0.0766	0.872	0.4863	0.2618	0.5261	0.1649		0.2129	0.0424	0.3848	0.1216	0.5899	0.1165	0.2593	0.4408	0.2011	0.6903	0.5167	0.0496
unclassified	0.0204	0.2788	0.5656	0.7543	0.5086	0.9834	0.9982	0.0242	0.6744	0.5553	0.9375	0.7247	0.1371	0.9477	0.3889	0.9168	0.9863	0.6832	0.8502	0.6529	0.2129		0.0717	0.6669	0.5232	0.5716	0.5207	0.7852	0.1721	0.5859	0.6618	0.1926	0.4422
unclassified	0.1299	0.234	0.2633	0.8826	0.5544	0.649	0.8613	0.1705	0.4046	0.8398	0.9206	0.9537	0.2163	0.6827	0.1509	0.9878	0.7181	0.4421	0.7988	0.3545	0.0424	0.0717		0.5202	0.2398	0.6066	0.2696	0.4761	0.2578	0.3596	0.9641	0.3127	0.1793
Erysipelotrichaceae	0.8628	0.1116	0.3767	0.1853	0.4337	0.9349	0.0646	0.8313	0.0999	0.3555	0.2265	0.5248	0.8515	0.4247	0.637	0.1394	0.0951	0.0504	0.3219	0.1961	0.3848	0.6669	0.5202		0.6202	0.1184	0.1377	0.1671	0.7849	0.0557	0.6665	0.7261	0.3099
unclassified	0.5879	0.5676	0.0435	0.9458	0.6092	0.1319	0.8705	0.6946	0.2796	0.7529	0.7143	0.4887	0.5241	0.2202	0.0139	0.9059	0.5182	0.3735	0.3673	0.1646	0.1216	0.5232	0.2398	0.6202		0.9931	0.2041	0.2429	0.5503	0.338	0.3612	0.6405	0.071
TM7_family_incertae_sedis	0.7332	0.1138	0.7509	0.2753	0.4244	0.6078	0.1685	0.6476	0.3981	0.671	0.1259	0.1715	0.9156	0.8788	0.9535	0.1512	0.3794	0.3038	0.7369	0.5441	0.5899	0.5716	0.6066	0.1184	0.9931		0.4192	0.5223	0.8528	0.2927	0.8599	0.8366	0.6273
unclassified	0.6842	0.1818	0.0686	0.4967	0.8435	0.4824	0.3171	0.722	0.0167	0.4433	0.6502	0.9687	0.8364	0.1969	0.2226	0.4884	0.1443	0.0357	0.2101	0.0167	0.1165	0.5207	0.2696	0.1377	0.2041	0.4192		0.0406	0.8942	0.0197	0.4324	0.9821	0.0398
"Porphyromonadaceae"	0.9567	0.3371	0.0881	0.3887	0.6995	0.3972	0.2652	0.9973	0.0132	0.2468	0.6271	0.909	0.9241	0.0773	0.3074	0.4356	0.063	0.0368	0.0694	0.0125	0.2593	0.7852	0.4761	0.1671	0.2429	0.5223	0.0406		0.8723	0.0492	0.2437	0.7795	0.0997
"Prevotellaceae"	0.0865	0.7787	0.7491	0.2622	0.1068	0.8106	0.4565	0.1357	0.9233	0.259	0.3979	0.8342	0.0023	0.7917	0.4205	0.3729	0.5763	0.8618	0.6031	0.9697	0.4408	0.1721	0.2578	0.7849	0.5503	0.8528	0.8942	0.8723		0.9629	0.6437	0.0049	0.684
"Rikenellaceae"	0.7687	0.1402	0.1541	0.3475	0.6658	0.628	0.1895	0.7822	0.0116	0.3689	0.4723	0.8098	0.9744	0.2422	0.3612	0.3301	0.0853	0.0055	0.2092	0.0484	0.2011	0.5859	0.3596	0.0557	0.338	0.2927	0.0197	0.0492	0.9629		0.4818	0.8823	0.1137
Bacteroidaceae	0.5662	0.9884	0.3014	0.6206	0.7921	0.164	0.628	0.4754	0.3591	0.2003	0.9844	0.3907	0.6554	0.0537	0.4976	0.7905	0.3114	0.4285	0.0816	0.293	0.6903	0.6618	0.9641	0.6665	0.3612	0.8599	0.4324	0.2437	0.6437	0.4818		0.5597	0.4193
unclassified	0.0948	0.8246	0.8453	0.2298	0.106	0.8939	0.4114	0.1352	0.8357	0.197	0.3953	0.8755	0.0108	0.6962	0.504	0.3485	0.4997	0.7809	0.5144	0.936	0.5167	0.1926	0.3127	0.7261	0.6405	0.8366	0.9821	0.7795	0.0049	0.8823	0.5597		0.7755
unclassified	0.5667	0.2844	0.014	0.7483	0.8868	0.3237	0.5468	0.635	0.0934	0.6021	0.9239	0.8183	0.6365	0.2005	0.0779	0.7487	0.2933	0.1455	0.2787	0.0423	0.0496	0.4422	0.1793	0.3099	0.071	0.6273	0.0398	0.0997	0.684	0.1137	0.4193	0.7755	

Ahr -/+

Genus v Genus Pearson correlation r

Genus	Values
Mucispirillum	[illegible]
unclassified	[illegible]
Enterorhabdus	[illegible]
unclassified	[illegible]
Parasutterella	[illegible]
unclassified	[illegible]
unclassified	[illegible]
unclassified	[illegible]
Vampirovibrio	[illegible]
unclassified	[illegible]
Bilophila	[illegible]
unclassified	[illegible]
unclassified	[illegible]
Helicobacter	[illegible]
unclassified	[illegible]
Anaeroplasma	[illegible]
Akkermansia	[illegible]
Lactobacillus	[illegible]
unclassified	[illegible]
Streptococcus	[illegible]
unclassified	[illegible]
Anaerosporobacter	[illegible]
Eubacterium	[illegible]
Dorea	[illegible]
Johnsonella	[illegible]
Lachnospiracea_incertae_sedis	[illegible]
Marvinbryantia	[illegible]
Anaerostipes	[illegible]
unclassified	[illegible]
Blautia	[illegible]
Clostridium_XlVa	[illegible]
Clostridium_XlVb	[illegible]
Flavonifractor	[illegible]
Hydrogenoanaerobacterium	[illegible]
Oscillibacter	[illegible]
Pseudoflavonifractor	[illegible]
Ruminococcus	[illegible]
Sporobacter	[illegible]
unclassified	[illegible]
Anaerotruncus	[illegible]
Butyricicoccus	[illegible]
Clostridium_IV	[illegible]
unclassified	[illegible]
unclassified	[illegible]
Allobaculum	[illegible]
Turicibacter	[illegible]
unclassified	[illegible]
unclassified	[illegible]
TM7_genus_incertae_sedis	[illegible]
unclassified	[illegible]
Barnesiella	[illegible]
unclassified	[illegible]
Odoribacter	[illegible]
Parabacteroides	[illegible]
unclassified	[illegible]
Alistipes	[illegible]
Bacteroides	[illegible]
unclassified	[illegible]
unclassified	[illegible]

Ahr -/+

Genus v Genus Pearson correlation p

Genus	Values
Mucispirillum	[illegible]
unclassified	[illegible]
Enterorhabdus	[illegible]
unclassified	[illegible]
Parasutterella	[illegible]
unclassified	[illegible]
unclassified	[illegible]
unclassified	[illegible]
Vampirovibrio	[illegible]
unclassified	[illegible]
Bilophila	[illegible]
unclassified	[illegible]
unclassified	[illegible]
Helicobacter	[illegible]
unclassified	[illegible]
Anaeroplasma	[illegible]
Akkermansia	[illegible]
Lactobacillus	[illegible]
unclassified	[illegible]
Streptococcus	[illegible]
unclassified	[illegible]
Anaerosporobacter	[illegible]
Eubacterium	[illegible]
Dorea	[illegible]
Johnsonella	[illegible]
Lachnospiracea_incertae_sedis	[illegible]
Marvinbryantia	[illegible]
Anaerostipes	[illegible]
unclassified	[illegible]
Blautia	[illegible]
Clostridium_XlVa	[illegible]
Clostridium_XlVb	[illegible]
Flavonifractor	[illegible]
Hydrogenoanaerobacterium	[illegible]
Oscillibacter	[illegible]
Pseudoflavonifractor	[illegible]
Ruminococcus	[illegible]
Sporobacter	[illegible]
unclassified	[illegible]
Anaerotruncus	[illegible]
Butyricicoccus	[illegible]
Clostridium_IV	[illegible]
unclassified	[illegible]
unclassified	[illegible]
Allobaculum	[illegible]
Turicibacter	[illegible]
unclassified	[illegible]
unclassified	[illegible]
TM7_genus_incertae_sedis	[illegible]
unclassified	[illegible]
Barnesiella	[illegible]
unclassified	[illegible]
Odoribacter	[illegible]
Parabacteroides	[illegible]
unclassified	[illegible]
Alistipes	[illegible]
Bacteroides	[illegible]
unclassified	[illegible]
unclassified	[illegible]

Ahr -/-

Genus v Genus Pearson correlation r

Genus	Values
Mucispirillum	[illegible]
unclassified	[illegible]
Enterorhabdus	[illegible]
unclassified	[illegible]
Parasutterella	[illegible]
unclassified	[illegible]
unclassified	[illegible]
unclassified	[illegible]
Vampirovibrio	[illegible]
unclassified	[illegible]
Bilophila	[illegible]
unclassified	[illegible]
unclassified	[illegible]
Helicobacter	[illegible]
unclassified	[illegible]
Anaeroplasma	[illegible]
Akkermansia	[illegible]
Lactobacillus	[illegible]
unclassified	[illegible]
Streptococcus	[illegible]
unclassified	[illegible]
Anaerosporobacter	[illegible]
Eubacterium	[illegible]
Dorea	[illegible]
Johnsonella	[illegible]
Lachnospiracea_incertae_sedis	[illegible]
Marvinbryantia	[illegible]
Anaerostipes	[illegible]
unclassified	[illegible]
Blautia	[illegible]
Clostridium_XlVa	[illegible]
Clostridium_XlVb	[illegible]
Flavonifractor	[illegible]
Hydrogenoanaerobacterium	[illegible]
Oscillibacter	[illegible]
Pseudoflavonifractor	[illegible]
Ruminococcus	[illegible]
Sporobacter	[illegible]
unclassified	[illegible]
Anaerotruncus	[illegible]
Butyricicoccus	[illegible]
Clostridium_IV	[illegible]
unclassified	[illegible]
unclassified	[illegible]
Allobaculum	[illegible]
Turicibacter	[illegible]
unclassified	[illegible]
unclassified	[illegible]
TM7_genus_incertae_sedis	[illegible]
unclassified	[illegible]
Barnesiella	[illegible]
unclassified	[illegible]
Odoribacter	[illegible]
Parabacteroides	[illegible]
unclassified	[illegible]
Alistipes	[illegible]
Bacteroides	[illegible]
unclassified	[illegible]
unclassified	[illegible]

Ahr -/-

[illegible]

Genus	Values
Mucispirillum	[illegible]
unclassified	[illegible]
Enterorhabdus	[illegible]
unclassified	[illegible]
Parasutterella	[illegible]
unclassified	[illegible]
unclassified	[illegible]
unclassified	[illegible]
Vampirovibrio	[illegible]
unclassified	[illegible]
Bilophila	[illegible]
unclassified	[illegible]
unclassified	[illegible]
Helicobacter	[illegible]
unclassified	[illegible]
Anaeroplasma	[illegible]
Akkermansia	[illegible]
Lactobacillus	[illegible]
unclassified	[illegible]
Streptococcus	[illegible]
unclassified	[illegible]
Anaerosporobacter	[illegible]
Eubacterium	[illegible]
Dorea	[illegible]
Johnsonella	[illegible]
Lachnospiracea_incertae_sedis	[illegible]
Marvinbryantia	[illegible]
Anaerostipes	[illegible]
unclassified	[illegible]
Blautia	[illegible]
Clostridium_XlVa	[illegible]
Clostridium_XlVb	[illegible]
Flavonifractor	[illegible]
Hydrogenoanaerobacterium	[illegible]
Oscillibacter	[illegible]
Pseudoflavonifractor	[illegible]
Ruminococcus	[illegible]
Sporobacter	[illegible]
unclassified	[illegible]
Anaerotruncus	[illegible]
Butyricicoccus	[illegible]
Clostridium_IV	[illegible]
unclassified	[illegible]
unclassified	[illegible]
Allobaculum	[illegible]
Turicibacter	[illegible]
unclassified	[illegible]
unclassified	[illegible]
TM7_genus_incertae_sedis	[illegible]
unclassified	[illegible]
Barnesiella	[illegible]
unclassified	[illegible]
Odoribacter	[illegible]
Parabacteroides	[illegible]
unclassified	[illegible]
Alistipes	[illegible]
Bacteroides	[illegible]
unclassified	[illegible]
unclassified	[illegible]

Ahr -/+

Phyla v Metabolite Pearson correlation r

	"Deferribacteres"	"Actinobacteria"	"Proteobacteria"	"Tenericutes"	"Verrucomicrobia"	Firmicutes	TM7	unclassified	"Bacteroidetes"
cecal butyrate	-0.1276	0.7912	-0.5523	-0.9127	0.4636	-0.9331	0.1062	-0.4008	0.9595
cecal propionate	0.14	0.8372	-0.3105	-0.8705	0.6856	-0.9554	0.1065	-0.4587	0.9413
cecal acetate	-0.969	-0.5069	-0.7771	-0.08862	-0.8543	0.1784	0.3047	0.5754	-0.04925
cecal baa	0.3779	0.6764	-0.04206	-0.814	0.8652	-0.9358	0.3102	-0.2819	0.8749
cecal lactate	0.2768	0.7587	-0.1641	-0.8551	0.7997	-0.962	0.2275	-0.3633	0.9211
cecal alanine	0.5469	0.6907	0.1414	-0.6871	0.9439	-0.852	0.2131	-0.3615	0.7693
cecal lysine	0.4778	0.4477	0.1283	-0.7196	0.8923	-0.8426	0.495	-0.05885	0.7558
cecal glutamate	0.3016	0.5298	0.2687	0.4529	0.0188	0.3143	-0.9916	-0.8774	-0.3132
cecal tyrosine	0.6465	0.7805	0.4316	0.1283	0.5419	-0.09901	-0.7746	-0.9512	0.04202
cecal phenylalanine	0.7941	0.6869	0.4469	-0.3871	0.9887	-0.6168	-0.03095	-0.5173	0.5059
cecal glucose	-0.1598	-0.2971	0.1611	0.8613	-0.6811	0.8941	-0.7053	-0.1792	-0.8454
cecal oligosacc	-0.6017	-0.229	-0.6849	-0.7238	-0.1205	-0.5413	0.8521	0.6583	0.596

Ahr -/+

Phyla v Metabolite Pearson correlation p

	"Deferribacteres"	"Actinobacteria"	"Proteobacteria"	"Tenericutes"	"Verrucomicrobia"	Firmicutes	TM7	unclassified	"Bacteroidetes"
cecal butyrate	0.8724	0.2088	0.4477	0.0873	0.5364	0.0669	0.8938	0.5992	0.0405
cecal propionate	0.86	0.1628	0.6895	0.1295	0.3144	0.0446	0.8935	0.5413	0.0587
cecal acetate	0.031	0.4931	0.2229	0.9114	0.1457	0.8216	0.6953	0.4246	0.9507
cecal baa	0.6221	0.3236	0.9579	0.186	0.1348	0.0642	0.6898	0.7181	0.1251
cecal lactate	0.7232	0.2413	0.8359	0.1449	0.2003	0.038	0.7725	0.6367	0.0789
cecal alanine	0.4531	0.3093	0.8586	0.3129	0.0561	0.148	0.7869	0.6385	0.2307
cecal lysine	0.5222	0.5523	0.8717	0.2804	0.1077	0.1574	0.505	0.9412	0.2442
cecal glutamate	0.6984	0.4702	0.7313	0.5471	0.9812	0.6857	0.0084	0.1226	0.6868
cecal tyrosine	0.3535	0.2195	0.5684	0.8717	0.4581	0.901	0.2254	0.0488	0.958
cecal phenylalanine	0.2059	0.3131	0.5531	0.6129	0.0113	0.3832	0.969	0.4827	0.4941
cecal glucose	0.8402	0.7029	0.8389	0.1387	0.3189	0.1059	0.2947	0.8208	0.1546
cecal oligosacc	0.3983	0.771	0.3151	0.2762	0.8795	0.4587	0.1479	0.3417	0.404

Ahr -/-

Phyla v Metabolite Pearson correlation r

	"Deferribacteres"	"Actinobacteria"	"Proteobacteria"	"Tenericutes"	"Verrucomicrobia"	Firmicutes	TM7	unclassified	"Bacteroidetes"
cecal butyrate	-0.7403	0.3043	-0.6262	0.8489	0.3548	-0.5793	0.1419	0.5085	0.5986
cecal propionate	0.8501	-0.2019	0.6751	-0.9665	-0.0385	0.2969	-0.2009	-0.2654	-0.3175
cecal acetate	0.5602	0.25	0.9325	-0.8141	0.02859	0.1102	-0.6193	0.05626	-0.1425
cecal baa	0.1826	0.6983	0.945	-0.5031	0.3127	-0.3103	-0.8929	0.5333	0.275
cecal lactate	0.7258	-0.1464	0.05366	-0.6532	0.8051	-0.5562	0.1538	0.3392	0.5583
cecal alanine	0.2855	0.6212	0.6592	-0.4962	0.7513	-0.6969	-0.6565	0.7666	0.673
cecal lysine	-0.1823	0.924	0.7121	-0.1128	0.5288	-0.6422	-0.9058	0.8498	0.6135
cecal glutamate	-0.2412	-0.6549	-0.9486	0.5543	-0.3139	0.2909	0.8657	-0.5025	-0.2559
cecal tyrosine	0.5151	0.128	0.8961	-0.7572	-0.2407	0.3675	-0.5485	-0.1764	-0.3986
cecal phenylalanine	0.3202	0.1557	0.8641	-0.5755	-0.4542	0.5153	-0.5733	-0.2715	-0.5463
cecal glucose	0.734	-0.9028	-0.1407	-0.5648	-0.4417	0.7135	0.621	-0.8704	-0.7034
cecal oligosacc	0.5201	-0.9876	-0.4686	-0.2637	-0.473	0.6902	0.8345	-0.8999	-0.6687

Ahr -/-

Phyla v Metabolite Pearson correlation p

	"Deferribacteres"	"Actinobacteria"	"Proteobacteria"	"Tenericutes"	"Verrucomicrobia"	Firmicutes	TM7	unclassified	"Bacteroidetes"
cecal butyrate	0.2597	0.6957	0.3738	0.1511	0.6452	0.4207	0.8581	0.4915	0.4014
cecal propionate	0.1499	0.7981	0.3249	0.0335	0.9615	0.7031	0.7991	0.7346	0.6825
cecal acetate	0.4398	0.75	0.0675	0.1859	0.9714	0.8898	0.3807	0.9437	0.8575
cecal baa	0.8174	0.3017	0.055	0.4969	0.6873	0.6897	0.1071	0.4667	0.725
cecal lactate	0.2742	0.8536	0.9463	0.3468	0.1949	0.4438	0.8462	0.6608	0.4417
cecal alanine	0.7145	0.3788	0.3408	0.5038	0.2487	0.3031	0.3435	0.2334	0.327
cecal lysine	0.8177	0.076	0.2879	0.8872	0.4712	0.3578	0.0942	0.1502	0.3865
cecal glutamate	0.7588	0.3451	0.0514	0.4457	0.6861	0.7091	0.1343	0.4975	0.7441
cecal tyrosine	0.4849	0.872	0.1039	0.2428	0.7593	0.6325	0.4515	0.8236	0.6014
cecal phenylalanine	0.6798	0.8443	0.1359	0.4245	0.5458	0.4847	0.4267	0.7285	0.4537
cecal glucose	0.266	0.0972	0.8593	0.4352	0.5583	0.2865	0.379	0.1296	0.2966
cecal oligosacc	0.4799	0.0124	0.5314	0.7363	0.527	0.3098	0.1655	0.1001	0.3313

Ahr -/+

Class v Metabolite Pearson correlation r

	Deferribacteres	Actinobacteria	Alphaproteobacteria	Betaproteobacteria	Deltaproteobacteria	Epsilonproteobacteria	unclassified	Mollicutes	Verrucomicrobiae	Bacilli	Clostridia	Erysipelotrichia	unclassified	TM7_class_incertae_sedis	unclassified	"Bacteroidia"	unclassified
cecal butyrate	-0.1276	0.7912	-0.8692	0.8787	0.4953	-0.5267	0.1998	-0.9127	0.4636	0.2482	-0.9256	-0.9748	-0.862	0.1062	-0.4008	0.9297	0.9318
cecal propionate	0.14	0.8372	-0.8266	0.8151	0.4544	-0.2796	0.4487	-0.8705	0.6856	0.09693	-0.9447	-0.95	-0.7031	0.1065	-0.4587	0.9221	0.8488
cecal acetate	-0.969	-0.5069	-0.1272	-0.1179	0.3233	-0.7063	-0.9897	-0.08862	-0.8543	0.7422	0.1555	0.04744	-0.3064	0.3047	0.5754	-0.0531	-0.01275
cecal baa	0.3779	0.6764	-0.7932	0.5526	0.5451	0.05298	0.6206	-0.814	0.8652	0.09453	-0.9296	-0.8632	-0.406	0.3102	-0.2819	0.8869	0.5929
cecal lactate	0.2768	0.7587	-0.8239	0.6788	0.5169	-0.09525	0.5533	-0.8551	0.7997	0.1001	-0.9538	-0.9181	-0.546	0.2275	-0.3633	0.9195	0.7173
cecal alanine	0.5469	0.6907	-0.6617	0.4954	0.4089	0.2116	0.7597	-0.6871	0.9439	-0.07708	-0.8419	-0.7579	-0.2752	0.2131	-0.3615	0.7819	0.5081
cecal lysine	0.4778	0.4477	-0.7237	0.2768	0.6174	0.2795	0.6451	-0.7196	0.8923	0.1508	-0.8427	-0.7272	-0.1461	0.495	-0.05885	0.794	0.3293
cecal glutamate	0.3016	0.5298	0.5366	0.4062	-0.892	-0.01412	0.3321	0.4529	0.0188	-0.8739	0.3461	0.2614	-0.1027	-0.9916	-0.8774	-0.3879	0.2504
cecal tyrosine	0.6465	0.7805	0.2123	0.5277	-0.6283	0.2176	0.7526	0.1283	0.5419	-0.8808	-0.06514	-0.07855	-0.1157	-0.7746	-0.9512	-0.01185	0.3862
cecal phenylalanine	0.7941	0.6869	-0.3508	0.3836	0.08958	0.4514	0.941	-0.3871	0.9887	-0.4092	-0.599	-0.4994	-0.0428	-0.03095	-0.5173	0.5133	0.3398
cecal glucose	-0.1598	-0.2971	0.8833	-0.2566	-0.8482	-0.04524	-0.3404	0.8613	-0.6811	-0.4806	0.9038	0.8123	0.2803	-0.7053	-0.1792	-0.8893	-0.3608
cecal oligosacc	-0.6017	-0.229	-0.7788	0.02915	0.9464	-0.4494	-0.4971	-0.7238	-0.1205	0.978	-0.5687	-0.5661	-0.3846	0.8521	0.6583	0.6355	0.1977

Ahr -/+

Class v Metabolite Pearson correlation p

	Deferribacteres	Actinobacteria	Alphaproteobacteria	Betaproteobacteria	Deltaproteobacteria	Epsilonproteobacteria	unclassified	Mollicutes	Verrucomicrobiae	Bacilli	Clostridia	Erysipelotrichia	unclassified	TM7_class_incertae_sedis	unclassified	"Bacteroidia"	unclassified
cecal butyrate	0.8724	0.2088	0.1308	0.1213	0.5047	0.4733	0.8002	0.0873	0.5364	0.7518	0.0744	0.0252	0.138	0.8938	0.5992	0.0703	0.0682
cecal propionate	0.86	0.1628	0.1734	0.1849	0.5456	0.7204	0.5513	0.1295	0.3144	0.9031	0.0553	0.05	0.2969	0.8935	0.5413	0.0779	0.1512
cecal acetate	0.031	0.4931	0.8728	0.8821	0.6767	0.2937	0.0103	0.9114	0.1457	0.2578	0.8445	0.9526	0.6936	0.6953	0.4246	0.9469	0.9873
cecal baa	0.6221	0.3236	0.2068	0.4474	0.4549	0.947	0.3794	0.186	0.1348	0.9055	0.0704	0.1368	0.594	0.6898	0.7181	0.1131	0.4071
cecal lactate	0.7232	0.2413	0.1761	0.3212	0.4831	0.9047	0.4467	0.1449	0.2003	0.8999	0.0462	0.0819	0.454	0.7725	0.6367	0.0805	0.2827
cecal alanine	0.4531	0.3093	0.3383	0.5046	0.5911	0.7884	0.2403	0.3129	0.0561	0.9229	0.1581	0.2421	0.7248	0.7869	0.6385	0.2181	0.4919
cecal lysine	0.5222	0.5523	0.2763	0.7232	0.3826	0.7205	0.3549	0.2804	0.1077	0.8492	0.1573	0.2728	0.8539	0.505	0.9412	0.206	0.6707
cecal glutamate	0.6984	0.4702	0.4634	0.5938	0.108	0.9859	0.6679	0.5471	0.9812	0.1261	0.6539	0.7386	0.8973	0.0084	0.1226	0.6121	0.7496
cecal tyrosine	0.3535	0.2195	0.7877	0.4723	0.3717	0.7824	0.2474	0.8717	0.4581	0.1192	0.9349	0.9215	0.8843	0.2254	0.0488	0.9881	0.6138
cecal phenylalanine	0.2059	0.3131	0.6492	0.6164	0.9104	0.5486	0.059	0.6129	0.0113	0.5908	0.401	0.5006	0.9572	0.969	0.4827	0.4867	0.6602
cecal glucose	0.8402	0.7029	0.1167	0.7434	0.1518	0.9548	0.6596	0.1387	0.3189	0.5194	0.0962	0.1877	0.7197	0.2947	0.8208	0.1107	0.6392
cecal oligosacc	0.3983	0.771	0.2212	0.9708	0.0536	0.5506	0.5029	0.2762	0.8795	0.022	0.4313	0.4339	0.6154	0.1479	0.3417	0.3645	0.8023

Ahr -/-

Class v Metabolite Pearson correlation r

	Deferribacteres	Actinobacteria	Alphaproteobacteria	Betaproteobacteria	Deltaproteobacteria	Epsilonproteobacteria	unclassified	Mollicutes	Verrucomicrobiae	Bacilli	Clostridia	Erysipelotrichia	unclassified	TM7_class_incertae_sedis	unclassified	"Bacteroidia"	unclassified
cecal butyrate	-0.7403	0.3043	0.708	0.749	-0.3594	-0.5646	-0.4586	0.8489	0.3548	-0.9189	-0.4578	0.05672	-0.8609	0.1419	0.5085	0.6275	-0.7198
cecal propionate	0.8501	-0.2019	-0.4582	-0.5328	0.2774	0.6496	0.349	-0.9665	-0.0385	0.7499	0.1679	0.1419	0.6541	-0.2009	-0.2654	-0.3555	0.4938
cecal acetate	0.5602	0.25	-0.245	-0.579	-0.1782	0.9214	0.6573	-0.8141	0.02859	0.5756	-0.0544	0.5196	0.5182	-0.6193	0.05626	-0.1596	0.2221
cecal baa	0.1826	0.6983	0.2188	-0.2975	-0.6371	0.9773	0.6895	-0.5031	0.3127	0.1093	-0.4665	0.8822	0.08031	-0.8929	0.5333	0.2775	-0.2761
cecal lactate	0.7258	-0.1464	0.4107	0.5246	0.2211	0.1358	-0.4933	-0.6532	0.8051	-0.1612	-0.5729	0.2531	-0.3177	0.1538	0.3392	0.5075	-0.2876
cecal alanine	0.2855	0.6212	0.5867	0.207	-0.5491	0.7436	0.2297	-0.4962	0.7513	-0.2473	-0.8043	0.8964	-0.3291	-0.6565	0.7666	0.6596	-0.5815
cecal lysine	-0.1823	0.924	0.6083	0.06324	-0.8852	0.7797	0.5176	-0.1128	0.5288	-0.3573	-0.7506	0.998	-0.3593	-0.9058	0.8498	0.6311	-0.6771
cecal glutamate	-0.2412	-0.6549	-0.1905	0.3071	0.59	-0.9797	-0.6727	0.5543	-0.3139	-0.1469	0.4489	-0.8601	-0.1102	0.8657	-0.5025	-0.2549	0.2408
cecal tyrosine	0.5151	0.128	-0.4821	-0.7731	-0.0705	0.853	0.7392	-0.7572	-0.2407	0.752	0.2074	0.3333	0.7205	-0.5485	-0.1764	-0.4107	0.4444
cecal phenylalanine	0.3202	0.1557	-0.59	-0.902	-0.119	0.7993	0.8545	-0.5755	-0.4542	0.7887	0.3626	0.253	0.7986	-0.5733	-0.2715	-0.545	0.5211
cecal glucose	0.734	-0.9028	-0.7963	-0.3914	0.9228	-0.2114	-0.2105	-0.5648	-0.4417	0.7886	0.7174	-0.7255	0.7225	0.621	-0.8704	-0.7446	0.8802
cecal oligosacc	0.5201	-0.9876	-0.7221	-0.2027	0.9805	-0.5376	-0.4281	-0.2637	-0.473	0.5922	0.752	-0.9147	0.5502	0.8345	-0.8999	-0.7027	0.8109

Ahr -/-

Class v Metabolite Pearson correlation p

	Deferribacteres	Actinobacteria	Alphaproteobacteria	Betaproteobacteria	Deltaproteobacteria	Epsilonproteobacteria	unclassified	Mollicutes	Verrucomicrobiae	Bacilli	Clostridia	Erysipelotrichia	unclassified	TM7_class_incertae_sedis	unclassified	"Bacteroidia"	unclassified
cecal butyrate	0.2597	0.6957	0.292	0.251	0.6406	0.4354	0.5414	0.1511	0.6452	0.0811	0.5422	0.9433	0.1391	0.8581	0.4915	0.3725	0.2802
cecal propionate	0.1499	0.7981	0.5418	0.4672	0.7226	0.3504	0.651	0.0335	0.9615	0.2501	0.8321	0.8581	0.3459	0.7991	0.7346	0.6445	0.5062
cecal acetate	0.4398	0.75	0.755	0.421	0.8218	0.0786	0.3427	0.1859	0.9714	0.4244	0.9456	0.4804	0.4818	0.3807	0.9437	0.8404	0.7779
cecal baa	0.8174	0.3017	0.7812	0.7025	0.3629	0.0227	0.3105	0.4969	0.6873	0.8907	0.5335	0.1178	0.9197	0.1071	0.4667	0.7225	0.7239
cecal lactate	0.2742	0.8536	0.5893	0.4754	0.7789	0.8642	0.5067	0.3468	0.1949	0.8388	0.4271	0.7469	0.6823	0.8462	0.6608	0.4925	0.7124
cecal alanine	0.7145	0.3788	0.4133	0.793	0.4509	0.2564	0.7703	0.5038	0.2487	0.7527	0.1957	0.1036	0.6709	0.3435	0.2334	0.3404	0.4185
cecal lysine	0.8177	0.076	0.3917	0.9368	0.1148	0.2203	0.4824	0.8872	0.4712	0.6427	0.2494	0.002	0.6407	0.0942	0.1502	0.3689	0.3229
cecal glutamate	0.7588	0.3451	0.8095	0.6929	0.41	0.0203	0.3273	0.4457	0.6861	0.8531	0.5511	0.1399	0.8898	0.1343	0.4975	0.7451	0.7592
cecal tyrosine	0.4849	0.872	0.5179	0.2269	0.9295	0.147	0.2608	0.2428	0.7593	0.248	0.7926	0.6667	0.2795	0.4515	0.8236	0.5893	0.5556
cecal phenylalanine	0.6798	0.8443	0.41	0.098	0.881	0.2007	0.1455	0.4245	0.5458	0.2113	0.6374	0.747	0.2014	0.4267	0.7285	0.455	0.4789
cecal glucose	0.266	0.0972	0.2037	0.6086	0.0772	0.7886	0.7895	0.4352	0.5583	0.2114	0.2826	0.2745	0.2775	0.379	0.1296	0.2554	0.1198
cecal oligosacc	0.4799	0.0124	0.2779	0.7973	0.0195	0.4624	0.5719	0.7363	0.527	0.4078	0.248	0.0853	0.4498	0.1655	0.1001	0.2973	0.1891

Ahr -/+
Order v Metabolite Pearson correlation r

	Deferribacterales	Coriobacteriales	unclassified	Burkholderiales	unclassified	Bdellovibrionales	unclassified	Desulfovibrionales	Campylobacterales	unclassified	Anaeroplasmatales	Verrucomicrobiales	Lactobacillales	Clostridiales	unclassified	Erysipelotrichales	unclassified	TM7_order_incertae_sedis	unclassified	"Bacteroidales"	unclassified
cecal butyrate	-0.1276	0.79	-0.8699	0.864	0.8833	0.2848	-0.01031	0.5375	-0.5267	0.1998	-0.9127	0.4636	0.2444	-0.9229	0.15	-0.9748	-0.862	0.1062	-0.4008	0.9297	0.9318
cecal propionate	0.14	0.8188	-0.8269	0.7806	0.9047	0.5264	-0.06527	0.4579	-0.2796	0.4487	-0.8705	0.6856	0.09264	-0.9436	0.3432	-0.95	-0.7031	0.1065	-0.4587	0.9221	0.8488
cecal acetate	-0.969	-0.4568	-0.1281	-0.04652	-0.4008	-0.8265	0.5165	0.432	-0.7063	-0.9897	-0.08862	-0.8543	0.7448	0.1591	-0.4849	0.04744	-0.3064	0.3047	0.5754	-0.0531	-0.01275
cecal baa	0.3779	0.6346	-0.7927	0.4993	0.735	0.7835	0.09302	0.4944	0.05298	0.6206	-0.814	0.8652	0.09	-0.9313	0.6622	-0.8632	-0.406	0.3102	-0.2819	0.8869	0.5929
cecal lactate	0.2768	0.7266	-0.8237	0.6329	0.8227	0.6823	0.02688	0.4895	-0.09525	0.5533	-0.8551	0.7997	0.09561	-0.9543	0.5326	-0.9181	-0.546	0.2275	-0.3633	0.9195	0.7173
cecal alanine	0.5469	0.6426	-0.6611	0.4328	0.7187	0.87	-0.02324	0.3392	0.2116	0.7597	-0.6871	0.9439	-0.08158	-0.8442	0.6938	-0.7579	-0.2752	0.2131	-0.3615	0.7819	0.5081
cecal lysine	0.4778	0.3924	-0.7226	0.2166	0.504	0.8862	0.2705	0.5319	0.2795	0.6451	-0.7196	0.8923	0.1465	-0.8465	0.8566	-0.7272	-0.1461	0.495	-0.05885	0.794	0.3293
cecal glutamate	0.3016	0.5531	0.5353	0.402	0.3976	-0.1419	-0.9927	-0.8574	-0.01412	0.3321	0.4529	0.0188	-0.8734	0.3498	-0.591	0.2614	-0.1027	-0.9916	-0.8774	-0.3879	0.2504
cecal tyrosine	0.6465	0.7707	0.2117	0.4863	0.663	0.3816	-0.9069	-0.6496	0.2176	0.7526	0.1283	0.5419	-0.8826	-0.06386	-0.1121	-0.07855	-0.1157	-0.7746	-0.9512	-0.01185	0.3862
cecal phenylalanine	0.7941	0.6344	-0.3501	0.3114	0.6532	0.9237	-0.2775	-0.002719	0.4514	0.941	-0.3871	0.9887	-0.4132	-0.602	0.6435	-0.4994	-0.0428	-0.03095	-0.5173	0.5133	0.3398
cecal glucose	-0.1598	-0.2531	0.8823	-0.216	-0.4053	-0.6893	-0.5326	-0.7922	-0.04524	-0.3404	0.8613	-0.6811	-0.4767	0.9069	-0.7998	0.8123	0.2803	-0.7053	-0.1792	-0.8893	-0.3608
cecal oligosacc	-0.6017	-0.2286	-0.7785	0.05086	-0.06091	-0.06975	0.8787	0.9768	-0.4494	-0.4971	-0.7238	-0.1205	0.9771	-0.569	0.3121	-0.5661	-0.3846	0.8521	0.6583	0.6355	0.1977

Ahr -/+
Order v Metabolite Pearson correlation p

	Deferribacterales	Coriobacteriales	unclassified	Burkholderiales	unclassified	Bdellovibrionales	unclassified	Desulfovibrionales	Campylobacterales	unclassified	Anaeroplasmatales	Verrucomicrobiales	Lactobacillales	Clostridiales	unclassified	Erysipelotrichales	unclassified	TM7_order_incertae_sedis	unclassified	"Bacteroidales"	unclassified
cecal butyrate	0.8724	0.21	0.1301	0.136	0.1167	0.7152	0.9897	0.4625	0.4733	0.8002	0.0873	0.5364	0.7556	0.0771	0.85	0.0252	0.138	0.8938	0.5992	0.0703	0.0682
cecal propionate	0.86	0.1812	0.1731	0.2194	0.0953	0.4736	0.9347	0.5421	0.7204	0.5513	0.1295	0.3144	0.9074	0.0564	0.6568	0.05	0.2969	0.8935	0.5413	0.0779	0.1512
cecal acetate	0.5432	0.8719	0.9535	0.5992	0.1735	0.4835	0.568	0.2937	0.0103	0.9114	0.1457	0.2552	0.8409	0.5151	0.9526	0.6936	0.6953	0.4246	0.9469	0.9873	
cecal baa	0.6221	0.3654	0.2073	0.5007	0.265	0.2165	0.907	0.5056	0.947	0.3794	0.186	0.1348	0.91	0.0687	0.3378	0.1368	0.594	0.6898	0.7181	0.1131	0.4071
cecal lactate	0.7232	0.2734	0.1763	0.3671	0.1773	0.3177	0.9731	0.5105	0.9047	0.4467	0.1449	0.2003	0.9044	0.0457	0.4674	0.0819	0.454	0.7725	0.6367	0.0805	0.2827
cecal alanine	0.4531	0.3574	0.3389	0.5672	0.2813	0.13	0.9768	0.6608	0.7884	0.2403	0.3129	0.0561	0.9184	0.1558	0.3062	0.2421	0.7248	0.7869	0.6385	0.2181	0.4919
cecal lysine	0.5222	0.6076	0.2774	0.7834	0.496	0.1138	0.7295	0.4681	0.7205	0.3549	0.2804	0.1077	0.8535	0.1535	0.1434	0.2728	0.8539	0.505	0.9412	0.206	0.6707
cecal glutamate	0.6984	0.4469	0.4647	0.598	0.6024	0.8581	0.0073	0.1426	0.9859	0.6679	0.5471	0.9812	0.1266	0.6502	0.409	0.7386	0.8973	0.0084	0.1226	0.6121	0.7496
cecal tyrosine	0.3535	0.2293	0.7883	0.5137	0.337	0.6184	0.0931	0.3504	0.7824	0.2474	0.8717	0.4581	0.1174	0.9361	0.8879	0.9215	0.8843	0.2254	0.0488	0.9881	0.6138
cecal phenylalanine	0.2059	0.3656	0.6499	0.6886	0.3468	0.0763	0.7225	0.9973	0.5486	0.059	0.6129	0.0113	0.5868	0.398	0.3565	0.5006	0.9572	0.969	0.4827	0.4867	0.6602
cecal glucose	0.8402	0.7469	0.1177	0.784	0.5947	0.3107	0.4674	0.2078	0.9548	0.6596	0.1387	0.3189	0.5233	0.0931	0.2002	0.1877	0.7197	0.2947	0.8208	0.1107	0.6392
cecal oligosacc	0.3983	0.7714	0.2215	0.9491	0.9391	0.9303	0.1213	0.0232	0.5506	0.5029	0.2762	0.8795	0.0229	0.431	0.6879	0.4339	0.6154	0.1479	0.3417	0.3645	0.8023

Ahr -/-
Order v Metabolite Pearson correlation r

	Deferribacterales	Coriobacteriales	unclassified	Burkholderiales	unclassified	Bdellovibrionales	unclassified	Desulfovibrionales	Campylobacterales	unclassified	Anaeroplasmatales	Verrucomicrobiales	Lactobacillales	Clostridiales	unclassified	Erysipelotrichales	unclassified	TM7_order_incertae_sedis	unclassified	"Bacteroidales"	unclassified
cecal butyrate	-0.7403	0.3299	0.7086	0.9794	0.662	0.275	-0.6421	-0.732	-0.5646	-0.4586	0.8486	0.3548	-0.9188	-0.443	-0.8506	0.05672	-0.8609	0.1419	0.5085	0.6275	-0.7198
cecal propionate	0.8501	-0.2333	-0.4577	-0.8883	-0.434	-0.4802	0.7723	0.6043	0.6496	0.349	-0.9663	-0.0385	0.7497	0.1504	0.7967	0.1419	0.6541	-0.2009	-0.2654	-0.3555	0.4938
cecal acetate	0.5602	0.2199	-0.2495	-0.6921	-0.5242	-0.7677	0.459	0.2283	0.9214	0.6573	-0.8141	0.02859	0.5754	-0.0666	0.4574	0.5196	0.5182	-0.6193	0.05626	-0.1596	0.2221
cecal baa	0.1826	0.6743	0.2116	-0.2249	-0.2946	-0.9711	0.1028	-0.3054	0.9773	0.6895	-0.5033	0.3127	0.1091	-0.4717	-0.05556	0.8822	0.08031	-0.8929	0.5333	0.2775	-0.2761
cecal lactate	0.7258	-0.17	0.4178	-0.1004	0.6151	-0.4181	0.7857	0.09498	0.1358	-0.4933	-0.6536	0.8051	-0.1615	-0.5887	0.3063	0.2531	-0.3177	0.1538	0.3392	0.5075	-0.2876
cecal alanine	0.2855	0.5967	0.584	0.04179	0.227	-0.9548	0.2732	-0.4302	0.7436	0.2297	-0.4966	0.7513	-0.2475	-0.812	-0.1494	0.8964	-0.3291	-0.6565	0.7666	0.6596	-0.5815
cecal lysine	-0.1823	0.9108	0.6007	0.2613	0.02152	-0.9237	-0.2189	-0.7148	0.7797	0.5176	-0.1131	0.5288	-0.3575	-0.7487	-0.5018	0.998	-0.3593	-0.9058	0.8498	0.6311	-0.6771
cecal glutamate	-0.2412	-0.6295	-0.1837	0.2715	0.2965	0.9691	-0.1612	0.2546	-0.9797	-0.6727	0.5544	-0.3139	-0.1467	0.4553	-0.001024	-0.8601	-0.1102	0.8657	-0.5025	-0.2549	0.2408
cecal tyrosine	0.5151	0.1016	-0.4871	-0.8095	-0.7221	-0.5927	0.396	0.3791	0.853	0.7392	-0.757	-0.2407	0.7519	0.1966	0.5505	0.3333	0.7205	-0.5485	-0.1764	-0.4107	0.4444
cecal phenylalanine	0.3202	0.1361	-0.5967	-0.7759	-0.8749	-0.4668	0.1872	0.3566	0.7993	0.8545	-0.5752	-0.4542	0.7886	0.356	0.4683	0.253	0.7986	-0.5733	-0.2715	-0.545	0.5211
cecal glucose	0.734	-0.9134	-0.7896	-0.8079	-0.2889	0.4352	0.7143	0.995	-0.2114	-0.2105	-0.5645	-0.4417	0.7886	0.7037	0.9519	-0.7255	0.7225	0.621	-0.8704	-0.7446	0.8802
cecal oligosacc	0.5201	-0.987	-0.714	-0.564	-0.1217	0.7152	0.5342	0.9138	-0.5376	-0.4281	-0.2634	-0.473	0.5923	0.743	0.785	-0.9147	0.5502	0.8345	-0.8999	-0.7027	0.8109

Ahr -/-
Order v Metabolite Pearson correlation p

	Deferribacterales	Coriobacteriales	unclassified	Burkholderiales	unclassified	Bdellovibrionales	unclassified	Desulfovibrionales	Campylobacterales	unclassified	Anaeroplasmatales	Verrucomicrobiales	Lactobacillales	Clostridiales	unclassified	Erysipelotrichales	unclassified	TM7_order_incertae_sedis	unclassified	"Bacteroidales"	unclassified
cecal butyrate	0.2597	0.6701	0.2914	0.0206	0.338	0.725	0.3579	0.268	0.4354	0.5414	0.1514	0.6452	0.0812	0.557	0.1494	0.9433	0.1391	0.8581	0.4915	0.3725	0.2802
cecal propionate	0.1499	0.7667	0.5423	0.1117	0.566	0.5198	0.2277	0.3957	0.3504	0.651	0.0337	0.9615	0.2503	0.8496	0.2033	0.8581	0.3459	0.7991	0.7346	0.6445	0.5062
cecal acetate	0.4398	0.7801	0.7505	0.3079	0.4758	0.2323	0.541	0.7717	0.0786	0.3427	0.1859	0.9714	0.4246	0.9334	0.5426	0.4804	0.4818	0.3807	0.9437	0.8404	0.7779
cecal baa	0.8174	0.3257	0.7884	0.7751	0.7054	0.0289	0.8972	0.6946	0.0227	0.3105	0.4967	0.6873	0.8909	0.5283	0.9444	0.1178	0.9197	0.1071	0.4667	0.7225	0.7239
cecal lactate	0.2742	0.83	0.5822	0.8996	0.3849	0.5819	0.2143	0.905	0.8642	0.5067	0.3464	0.1949	0.8385	0.4113	0.6937	0.7469	0.6823	0.8462	0.6608	0.4925	0.7124
cecal alanine	0.7145	0.4033	0.416	0.9582	0.773	0.0452	0.7268	0.5698	0.2564	0.7703	0.5034	0.2487	0.7525	0.188	0.8506	0.1036	0.6709	0.3435	0.2334	0.3404	0.4185
cecal lysine	0.8177	0.0892	0.3993	0.7387	0.9785	0.0763	0.7811	0.2852	0.2203	0.4824	0.8869	0.4712	0.6425	0.2513	0.4982	0.002	0.6407	0.0942	0.1502	0.3689	0.3229
cecal glutamate	0.7588	0.3705	0.8163	0.7285	0.7035	0.0309	0.8388	0.7454	0.0203	0.3273	0.4456	0.6861	0.8533	0.5447	0.999	0.1399	0.8898	0.1343	0.4975	0.7451	0.7592
cecal tyrosine	0.4849	0.8984	0.5129	0.1905	0.2779	0.4073	0.604	0.6209	0.147	0.2608	0.243	0.7593	0.2481	0.8034	0.4495	0.6667	0.2795	0.4515	0.8236	0.5893	0.5556
cecal phenylalanine	0.6798	0.8639	0.4033	0.2241	0.1251	0.5332	0.8128	0.6434	0.2007	0.1455	0.4248	0.5458	0.2114	0.644	0.5317	0.747	0.2014	0.4267	0.7285	0.455	0.4789
cecal glucose	0.266	0.0866	0.2104	0.1921	0.7111	0.5648	0.2857	0.005	0.7886	0.7895	0.4355	0.5583	0.2114	0.2963	0.0481	0.2745	0.2775	0.379	0.1296	0.2554	0.1198
cecal oligosacc	0.4799	0.013	0.286	0.436	0.8783	0.2848	0.4658	0.0862	0.4624	0.5719	0.7366	0.527	0.4077	0.257	0.215	0.0853	0.4498	0.1655	0.1001	0.2973	0.1891

Ahr -/+

Family v Metabolite Pearson correlation r

	Deferribacteraceae	Coriobacteriaceae	unclassified	Sutterellaceae	unclassified	unclassified	Bdellovibrionaceae	unclassified	Desulfovibrionaceae	unclassified	Helicobacteraceae	unclassified	Anaeroplasmataceae	Verrucomicrobiaceae	Lactobacillaceae	Streptococcaceae	unclassified	Clostridiaceae_1	Eubacteriaceae	Lachnospiraceae	Ruminococcaceae	unclassified	unclassified	Erysipelotrichaceae	unclassified	TM7_family_incertae_sedis	unclassified	"Porphyromonadaceae"	"Prevotellaceae"	"Rikenellaceae"	Bacteroidaceae	unclassified	unclassified
cecal butyrate	-0.1276	0.79	-0.8699	0.9946	0.273	0.8833	0.2843	-0.01031	0.5009	0.4205	-0.527	0.1998	-0.9127	0.4636	0.2322	0.2197	0.7279	-0.3942	-0.9596	-0.8317	0.09257	0.05953	0.15	-0.9748	-0.862	0.1062	-0.4008	0.9301	-0.2003	-0.1451	-0.3826	0.5598	0.9318
cecal propionate	0.14	0.8188	-0.8269	0.9601	0.1454	0.9047	0.5265	-0.06527	0.4605	0.1595	-0.28	0.4487	-0.8705	0.6856	0.08244	0.438	0.518	-0.5495	-0.865	-0.8927	0.1932	0.3242	0.3432	-0.95	-0.7031	0.1065	-0.4587	0.8293	-0.3237	-0.2569	-0.1412	0.7608	0.8488
cecal acetate	-0.969	-0.4568	-0.1281	-0.1247	0.1022	-0.4008	-0.8318	0.5165	0.3193	0.8281	-0.7061	-0.9897	-0.08862	-0.8543	0.7427	-0.6402	0.5375	0.8446	-0.185	0.2432	-0.06622	-0.8833	-0.4849	0.04744	-0.3064	0.3047	0.5754	0.2444	0.7527	0.713	-0.8856	-0.7786	-0.01275
cecal baa	0.3779	0.6346	-0.7927	0.7916	-0.1983	0.735	0.7823	0.09302	0.5505	-0.1415	0.05254	0.6206	-0.814	0.8652	0.08477	0.7394	0.2016	-0.5148	-0.7015	-0.9552	0.482	0.614	0.6622	-0.8632	-0.406	0.3102	-0.2819	0.6836	-0.2398	-0.1594	0.05893	0.9237	0.5929
cecal lactate	0.2768	0.7266	-0.8237	0.8809	-0.0502	0.8227	0.6817	0.02688	0.5227	-0.008461	-0.09569	0.5533	-0.8551	0.7997	0.08808	0.6185	0.347	-0.5371	-0.788	-0.9454	0.3638	0.495	0.5326	-0.9181	-0.546	0.2275	-0.3633	0.7621	-0.2788	-0.2029	-0.03141	0.867	0.7173
cecal alanine	0.5469	0.6426	-0.6611	0.7087	-0.209	0.7187	0.8701	-0.02324	0.4144	-0.3134	0.2112	0.7597	-0.6871	0.9439	-0.08548	0.7935	0.04695	-0.6279	-0.5641	-0.8862	0.4459	0.7361	0.6938	-0.7579	-0.2752	0.2131	-0.3615	0.5365	-0.3667	-0.2877	0.2497	0.9779	0.5081
cecal lysine	0.4778	0.3924	-0.7226	0.5819	-0.4777	0.504	0.8834	0.2705	0.6214	-0.324	0.2791	0.6451	-0.7196	0.8923	0.1458	0.8999	-0.04175	-0.3778	-0.529	-0.927	0.698	0.7514	0.8566	-0.7272	-0.1461	0.495	-0.05885	0.533	-0.08536	-0.001823	0.1535	0.9389	0.3293
cecal glutamate	0.3016	0.5531	0.5353	0.0551	0.7963	0.3976	-0.1322	-0.9927	-0.8891	-0.141	-0.01407	0.3321	0.4529	0.0188	-0.8795	-0.4242	0.0122	-0.7099	0.2463	0.4428	-0.8786	-0.05326	-0.591	0.2614	-0.1027	-0.9916	-0.8774	-0.3426	-0.8852	-0.9202	0.4861	-0.06148	0.2504
cecal tyrosine	0.6465	0.7707	0.2117	0.3283	0.5337	0.663	0.3904	-0.9069	-0.6231	-0.4039	0.2175	0.7526	0.1283	0.5419	-0.8884	0.08984	-0.0888	-0.9731	0.02992	-0.02343	-0.5205	0.4154	-0.1121	-0.07855	-0.1157	-0.7746	-0.9512	-0.1291	-0.9974	-0.9879	0.6668	0.4743	0.3862
cecal phenylalanine	0.7941	0.6344	-0.3501	0.5125	-0.1546	0.6532	0.9264	-0.2775	0.09495	-0.5762	0.451	0.941	-0.3871	0.9887	-0.415	0.7823	-0.2099	-0.8034	-0.2715	-0.6636	0.288	0.866	0.6435	-0.4994	-0.0428	-0.03095	-0.5173	0.2242	-0.6002	-0.5328	0.5719	0.9741	0.3398
cecal glucose	-0.1598	-0.2531	0.8823	-0.6093	0.5238	-0.4053	-0.6839	-0.5326	-0.8512	0.04445	-0.04486	-0.3404	0.8613	-0.6811	-0.4753	-0.7844	-0.1591	0.08306	0.6768	0.9683	-0.7894	-0.5124	-0.7998	0.8123	0.2803	-0.7053	-0.1792	-0.7069	-0.2176	-0.2979	0.1804	-0.7652	-0.3608
cecal oligosacc	-0.6017	-0.2286	-0.7785	0.323	-0.4181	-0.06091	-0.07863	0.8787	0.9453	0.5583	-0.4495	-0.4971	-0.7238	-0.1205	0.9757	0.1685	0.4742	0.6355	-0.6298	-0.5686	0.6378	-0.2441	0.3121	-0.5661	-0.3846	0.8521	0.6583	0.7066	0.8069	0.8405	-0.8075	0.001888	0.1977

Ahr -/+

Family v Metabolite Pearson correlation p

	Deferribacteraceae	Coriobacteriaceae	unclassified	Sutterellaceae	unclassified	unclassified	Bdellovibrionaceae	unclassified	Desulfovibrionaceae	unclassified	Helicobacteraceae	unclassified	Anaeroplasmataceae	Verrucomicrobiaceae	Lactobacillaceae	Streptococcaceae	unclassified	Clostridiaceae_1	Eubacteriaceae	Lachnospiraceae	Ruminococcaceae	unclassified	unclassified	Erysipelotrichaceae	unclassified	TM7_family_incertae_sedis	unclassified	"Porphyromonadaceae"	"Prevotellaceae"	"Rikenellaceae"	Bacteroidaceae	unclassified	unclassified
cecal butyrate	0.8724	0.21	0.1301	0.0054	0.727	0.1167	0.7157	0.9897	0.4991	0.5795	0.473	0.8002	0.0873	0.5364	0.7678	0.7803	0.2721	0.6058	0.0404	0.1683	0.9074	0.9405	0.85	0.0252	0.138	0.8938	0.5992	0.0699	0.7997	0.8549	0.6174	0.4402	0.0682
cecal propionate	0.86	0.1812	0.1731	0.0399	0.8546	0.0953	0.4735	0.9347	0.5395	0.8405	0.72	0.5513	0.1295	0.3144	0.9176	0.562	0.482	0.4505	0.135	0.1073	0.8068	0.6758	0.6568	0.05	0.2969	0.8935	0.5413	0.1707	0.6763	0.7431	0.8588	0.2392	0.1512
cecal acetate	0.031	0.5432	0.8719	0.8753	0.8978	0.5992	0.1682	0.4835	0.6807	0.1719	0.2939	0.0103	0.9114	0.1457	0.2573	0.3598	0.4625	0.1554	0.815	0.7568	0.9338	0.1167	0.5151	0.9526	0.6936	0.6953	0.4246	0.7556	0.2473	0.287	0.1144	0.2214	0.9873
cecal baa	0.6221	0.3654	0.2073	0.2084	0.8017	0.265	0.2177	0.907	0.4495	0.8585	0.9475	0.3794	0.186	0.1348	0.9152	0.2606	0.7984	0.4852	0.2985	0.0448	0.518	0.386	0.3378	0.1368	0.594	0.6898	0.7181	0.3164	0.7602	0.8406	0.9411	0.0763	0.4071
cecal lactate	0.7232	0.2734	0.1763	0.1191	0.9498	0.1773	0.3183	0.9731	0.4773	0.9915	0.9043	0.4467	0.1449	0.2003	0.9119	0.3815	0.653	0.4629	0.212	0.0546	0.6362	0.505	0.4674	0.0819	0.454	0.7725	0.6367	0.2379	0.7212	0.7971	0.9686	0.133	0.2827
cecal alanine	0.4531	0.3574	0.3389	0.2913	0.791	0.2813	0.1299	0.9768	0.5856	0.6866	0.7888	0.2403	0.3129	0.0561	0.9145	0.2065	0.9531	0.3721	0.4359	0.1138	0.5541	0.2639	0.3062	0.2421	0.7248	0.7869	0.6385	0.4635	0.6333	0.7123	0.7503	0.0221	0.4919
cecal lysine	0.5222	0.6076	0.2774	0.4181	0.5223	0.496	0.1166	0.7295	0.3786	0.676	0.7209	0.3549	0.2804	0.1077	0.8542	0.1001	0.9583	0.6222	0.471	0.073	0.302	0.2486	0.1434	0.2728	0.8539	0.505	0.9412	0.467	0.9146	0.9982	0.8465	0.0611	0.6707
cecal glutamate	0.4469	0.4647	0.9449	0.2037	0.6024	0.8678	0.0073	0.1109	0.859	0.9859	0.6679	0.5471	0.9812	0.1205	0.5758	0.9878	0.2901	0.7537	0.5572	0.1214	0.9467	0.409	0.7386	0.8973	0.0084	0.1226	0.6574	0.1148	0.0798	0.5139	0.9385	0.7496	
cecal tyrosine	0.3535	0.2293	0.7883	0.6717	0.4663	0.337	0.6096	0.0931	0.3769	0.5961	0.7825	0.2474	0.8717	0.4581	0.1116	0.9102	0.9112	0.0269	0.9701	0.9766	0.4795	0.5846	0.8879	0.9215	0.8843	0.2254	0.0488	0.8709	0.0026	0.0121	0.3332	0.5257	0.6138
cecal phenylalanine	0.2059	0.3656	0.6499	0.4875	0.8454	0.3468	0.0736	0.7225	0.905	0.4238	0.549	0.059	0.6129	0.0113	0.585	0.2177	0.7901	0.1966	0.7285	0.3364	0.712	0.134	0.3565	0.5006	0.9572	0.969	0.4827	0.7758	0.3998	0.4672	0.4281	0.0259	0.6602
cecal glucose	0.8402	0.7469	0.1177	0.3907	0.4762	0.5947	0.3161	0.4674	0.1488	0.9555	0.9551	0.6596	0.1387	0.3189	0.5247	0.2156	0.8409	0.9169	0.3232	0.0317	0.2106	0.4876	0.2002	0.1877	0.7197	0.2947	0.8208	0.2931	0.7824	0.7021	0.8196	0.2348	0.6392
cecal oligosacc	0.3983	0.7714	0.2215	0.677	0.5819	0.9391	0.9214	0.1213	0.0547	0.4417	0.5505	0.5029	0.2762	0.8795	0.0243	0.8315	0.5258	0.3645	0.3702	0.4314	0.3622	0.7559	0.6879	0.4339	0.6154	0.1479	0.3417	0.2934	0.1931	0.1595	0.1925	0.9981	0.8023

Ahr -/-

Family v Metabolite Pearson correlation r

	Deferribacteraceae	Coriobacteriaceae	unclassified	Sutterellaceae	unclassified	unclassified	Bdellovibrionaceae	unclassified	Desulfovibrionaceae	unclassified	Helicobacteraceae	unclassified	Anaeroplasmataceae	Verrucomicrobiaceae	Lactobacillaceae	Streptococcaceae	unclassified	Clostridiaceae_1	Eubacteriaceae	Lachnospiraceae	Ruminococcaceae	unclassified	unclassified	Erysipelotrichaceae	unclassified	TM7_family_incertae_sedis	unclassified	"Porphyromonadaceae"	"Prevotellaceae"	"Rikenellaceae"	Bacteroidaceae	unclassified	unclassified
cecal butyrate	-0.7403	0.3299	0.7086	-0.488	0.7705	0.662	0.275	-0.6421	-0.36	-0.2796	-0.5646	-0.4586	0.8486	0.3548	-0.9216	-0.4593	-0.02079	-0.2624	-0.1512	-0.4991	-0.8208	0.7221	-0.8506	0.05672	-0.8609	0.1419	0.5085	0.3623	0.838	-0.3393	0.2035	0.781	-0.7198
cecal propionate	0.8501	-0.2333	-0.4577	0.6842	-0.8957	-0.434	-0.4802	0.7723	0.09152	0.5695	0.6496	0.349	-0.9663	-0.0385	0.7543	0.614	-0.2644	0.006447	-0.1705	0.2244	0.6775	-0.7846	0.7967	0.1419	0.6541	-0.2009	-0.2654	-0.06976	-0.966	0.09912	0.1025	-0.9377	0.4938
cecal acetate	0.5602	0.2199	-0.2495	0.8821	-0.9824	-0.5242	-0.7677	0.459	-0.2055	0.6104	0.9214	0.6573	-0.8141	0.02859	0.5807	0.8822	-0.4993	-0.3166	-0.261	-0.03239	0.3532	-0.4331	0.4574	0.5196	0.5182	-0.6193	0.05626	0.1664	-0.8427	-0.2473	0.02185	-0.8278	0.2221
cecal baa	0.1826	0.6743	0.2116	0.9556	-0.8646	-0.2946	-0.9711	0.1028	-0.6315	0.6757	0.9773	0.6895	-0.5033	0.3127	0.115	0.996	-0.7877	-0.7264	-0.5029	-0.4745	-0.1776	0.001577	-0.05556	0.8822	0.08031	-0.8929	0.5333	0.5607	-0.5595	-0.6898	0.1536	-0.5829	-0.2761
cecal lactate	0.7258	-0.17	0.4178	0.5117	-0.4554	0.6151	-0.4181	0.7857	-0.4661	0.8804	0.1358	-0.4933	-0.6536	0.8051	-0.1563	0.3232	-0.6385	-0.4267	-0.8433	-0.4901	0.02288	-0.6344	0.3063	0.2531	-0.3177	0.1538	0.3392	0.5888	-0.6571	-0.3442	0.9322	-0.7187	-0.2876
cecal alanine	0.2855	0.5967	0.584	0.924	-0.7373	0.227	-0.9548	0.2732	-0.8681	0.9165	0.7436	0.2297	-0.4966	0.7513	-0.2408	0.8783	-0.9859	-0.9032	-0.8745	-0.7842	-0.3795	-0.08877	-0.1494	0.8964	-0.3291	-0.6565	0.7666	0.8655	-0.5498	-0.855	0.6303	-0.6155	-0.5815
cecal lysine	-0.1823	0.9108	0.6007	0.7963	-0.5497	0.02152	-0.9237	-0.2189	-0.8808	0.598	0.7797	0.5176	-0.1131	0.5288	-0.3524	0.8552	-0.8764	-0.9342	-0.632	-0.7807	-0.6208	0.3752	-0.5018	0.998	-0.3593	-0.9058	0.8498	0.8045	-0.1804	-0.9337	0.276	-0.2357	-0.6771
cecal glutamate	-0.2412	-0.6295	-0.1837	-0.9685	0.8928	0.2965	0.9691	-0.1612	0.6098	-0.7004	-0.9797	-0.6727	0.5544	-0.3139	-0.1528	-0.9992	0.7846	0.7052	0.5098	0.4516	0.1289	0.05868	-0.001024	-0.8601	-0.1102	0.8657	-0.5025	-0.5456	0.6084	0.6628	-0.1723	0.6304	0.2408
cecal tyrosine	0.5151	0.1016	-0.4871	0.7272	-0.9008	-0.7221	-0.5927	0.396	0.04109	0.3842	0.853	0.7392	-0.757	-0.2407	0.7558	0.7549	-0.2544	-0.0815	0.007763	0.2209	0.5159	-0.4296	0.5505	0.3333	0.7205	-0.5485	-0.1764	-0.09545	-0.775	-0.0208	-0.2246	-0.7345	0.4444
cecal phenylalanine	0.3202	0.1361	-0.5967	0.5763	-0.765	-0.8749	-0.4668	0.1872	0.1715	0.1426	0.7993	0.8545	-0.5752	-0.4542	0.7911	0.6525	-0.07516	0.04061	0.2351	0.3545	0.5097	-0.2616	0.4683	0.253	0.7986	-0.5733	-0.2715	-0.2566	-0.5914	0.07892	-0.4691	-0.5347	0.5211
cecal glucose	0.734	-0.9134	-0.7896	-0.1921	-0.1508	-0.2889	0.4352	0.7143	0.7774	-0.09397	-0.2114	-0.2105	-0.5645	-0.4417	0.7876	-0.2924	0.5332	0.7663	0.3875	0.7819	0.9637	-0.8478	0.9519	-0.7255	0.7225	0.621	-0.8704	-0.6965	-0.5087	0.8295	-0.1355	-0.4457	0.8802
cecal oligosacc	0.5201	-0.987	-0.714	-0.5142	0.2046	-0.1217	0.7152	0.5342	0.8622	-0.3304	-0.5376	-0.4281	-0.2634	-0.473	0.5893	-0.6098	0.725	0.889	0.5011	0.8068	0.8406	-0.6809	0.785	-0.9147	0.5502	0.8345	-0.8999	-0.7708	-0.1974	0.9259	-0.1673	-0.138	0.8109

Ahr -/-

Family v Metabolite Pearson correlation p

	Deferribacteraceae	Coriobacteriaceae	unclassified	Sutterellaceae	unclassified	unclassified	Bdellovibrionaceae	unclassified	Desulfovibrionaceae	unclassified	Helicobacteraceae	unclassified	Anaeroplasmataceae	Verrucomicrobiaceae	Lactobacillaceae	Streptococcaceae	unclassified	Clostridiaceae_1	Eubacteriaceae	Lachnospiraceae	Ruminococcaceae	unclassified	unclassified	Erysipelotrichaceae	unclassified	TM7_family_incertae_sedis	unclassified	"Porphyromonadaceae"	"Prevotellaceae"	"Rikenellaceae"	Bacteroidaceae	unclassified	unclassified
cecal butyrate	0.2597	0.6701	0.2914	0.512	0.2295	0.338	0.725	0.3579	0.64	0.7204	0.4354	0.5414	0.1514	0.6452	0.0784	0.5407	0.9792	0.7376	0.8488	0.5009	0.1792	0.2779	0.1494	0.9433	0.1391	0.8581	0.4915	0.6377	0.162	0.6607	0.7965	0.219	0.2802
cecal propionate	0.1499	0.7667	0.5423	0.3158	0.1043	0.566	0.5198	0.2277	0.9085	0.4305	0.3504	0.651	0.0337	0.9615	0.2457	0.386	0.7356	0.9936	0.8295	0.7756	0.3225	0.2154	0.2033	0.8581	0.3459	0.7991	0.7346	0.9302	0.034	0.9009	0.8975	0.0623	0.5062
cecal acetate	0.4398	0.7801	0.7505	0.1179	0.0176	0.4758	0.2323	0.541	0.7945	0.3896	0.0786	0.3427	0.1859	0.9714	0.4193	0.1178	0.5007	0.6834	0.739	0.9676	0.6468	0.5669	0.5426	0.4804	0.4818	0.3807	0.9437	0.8336	0.1573	0.7527	0.9782	0.1722	0.7779
cecal baa	0.8174	0.3257	0.7884	0.0444	0.1354	0.7054	0.0289	0.8972	0.3685	0.3243	0.0227	0.3105	0.4967	0.6873	0.885	0.004	0.2123	0.2736	0.4971	0.5255	0.8224	0.9984	0.9444	0.1178	0.9197	0.1071	0.4667	0.4393	0.4405	0.3102	0.8464	0.4171	0.7239
cecal lactate	0.2742	0.83	0.5822	0.4883	0.5446	0.3849	0.5819	0.2143	0.5339	0.1196	0.8642	0.5067	0.3464	0.1949	0.8437	0.6768	0.3615	0.5733	0.1567	0.5099	0.9771	0.3656	0.6937	0.7469	0.6823	0.8462	0.6608	0.4112	0.3429	0.6558	0.0678	0.2813	0.7124
cecal alanine	0.7145	0.4033	0.416	0.076	0.2627	0.773	0.0452	0.7268	0.1319	0.0835	0.2564	0.7703	0.5034	0.2487	0.7592	0.1217	0.0141	0.0968	0.1255	0.2158	0.6205	0.9112	0.8506	0.1036	0.6709	0.3435	0.2334	0.1345	0.4502	0.145	0.3697	0.3845	0.4185
cecal lysine	0.8177	0.0892	0.3993	0.2037	0.4503	0.9785	0.0763	0.7811	0.1192	0.402	0.2203	0.4824	0.8869	0.4712	0.6476	0.1448	0.1236	0.0658	0.368	0.2193	0.3792	0.6248	0.4982	0.002	0.6407	0.0942	0.1502	0.1955	0.8196	0.0663	0.724	0.7643	0.3229
cecal glutamate	0.7588	0.3705	0.8163	0.0315	0.1072	0.7035	0.0309	0.8388	0.3902	0.2996	0.0203	0.3273	0.4456	0.6861	0.8472	0.0008	0.2154	0.2948	0.4902	0.5484	0.8711	0.9413	0.999	0.1399	0.8898	0.1343	0.4975	0.4544	0.3916	0.3372	0.8277	0.3696	0.7592
cecal tyrosine	0.4849	0.8984	0.5129	0.2728	0.0992	0.2779	0.4073	0.604	0.9589	0.6158	0.147	0.2608	0.243	0.7593	0.2442	0.2451	0.7456	0.9185	0.9922	0.7791	0.4841	0.5704	0.4495	0.6667	0.2795	0.4515	0.8236	0.9045	0.225	0.9792	0.7754	0.2655	0.5556
cecal phenylalanine	0.6798	0.8639	0.4033	0.4237	0.235	0.1251	0.5332	0.8128	0.8285	0.8574	0.2007	0.1455	0.4248	0.5458	0.2089	0.3475	0.9248	0.9594	0.7649	0.6455	0.4903	0.7384	0.5317	0.747	0.2014	0.4267	0.7285	0.7434	0.4086	0.9211	0.5309	0.4653	0.4789
cecal glucose	0.266	0.0866	0.2104	0.8079	0.8492	0.7111	0.5648	0.2857	0.2226	0.906	0.7886	0.7895	0.4355	0.5583	0.2124	0.7076	0.4668	0.2337	0.6125	0.2181	0.0363	0.1522	0.0481	0.2745	0.2775	0.379	0.1296	0.3035	0.4913	0.1705	0.8645	0.5543	0.1198
cecal oligosacc	0.4799	0.013	0.286	0.4858	0.7954	0.8783	0.2848	0.4658	0.1378	0.6696	0.4624	0.5719	0.7366	0.527	0.4107	0.3902	0.275	0.111	0.4989	0.1932	0.1594	0.3191	0.215	0.0853	0.4498	0.1655	0.1001	0.2292	0.8026	0.0741	0.8327	0.862	0.1891

Ahr -/+
Genus v Metabolite Pearson correlation r

	Mucispirillum	unclassified	Enterorhabdus	unclassified	Parasutterella	unclassified	unclassified	unclassified	Vampirovibrio	unclassified	Bilophila	unclassified	unclassified	Helicobacter	unclassified
cecal butyrate	-0.1276	0.002636	0.942	-0.8699	0.642	0.8012	0.273	0.8833	0.2843	-0.01031	0.2692	0.5093	0.4205	-0.5281	0.1998
cecal propionate	0.14	0.1705	0.8184	-0.8269	0.6599	0.7484	0.1454	0.9047	0.5265	-0.06527	0.1451	0.4755	0.1595	-0.2812	0.4487
cecal acetate	-0.969	-0.843	0.206	-0.1281	0.05008	-0.1817	0.1022	-0.4008	-0.8318	0.5165	0.6606	0.2937	0.8281	-0.7057	-0.9897
cecal baa	0.3779	0.1679	0.5732	-0.7927	0.7682	0.4776	-0.1983	0.735	0.7823	0.09302	0.1786	0.5704	-0.1415	0.05127	0.6206
cecal lactate	0.2768	0.1692	0.6933	-0.8237	0.7356	0.6057	-0.0502	0.8227	0.6817	0.02688	0.1697	0.5407	-0.008461	-0.09693	0.5533
cecal alanine	0.5469	0.3269	0.4444	-0.6611	0.6625	0.4434	-0.209	0.7187	0.8701	-0.02324	0.01507	0.4375	-0.3134	0.21	0.7597
cecal lysine	0.4778	0.08146	0.3392	-0.7226	0.8178	0.1937	-0.4777	0.504	0.8834	0.2705	0.2537	0.6422	-0.324	0.2778	0.6451
cecal glutamate	0.3016	0.841	-0.004979	0.5353	-0.7624	0.5409	0.7963	0.3976	-0.1322	-0.9927	-0.9159	-0.884	-0.141	-0.01346	0.3321
cecal tyrosine	0.6465	0.9672	0.1134	0.2117	-0.3745	0.6294	0.5337	0.663	0.3904	-0.9069	-0.863	-0.6052	-0.4039	0.2174	0.7526
cecal phenylalanine	0.7941	0.6136	0.193	-0.3501	0.3787	0.383	-0.1546	0.6532	0.9264	-0.2775	-0.3149	0.1214	-0.5762	0.45	0.941
cecal glucose	-0.1598	0.2555	-0.4633	0.8823	-0.9663	-0.1342	0.5238	-0.4053	-0.6839	-0.5326	-0.5668	-0.8647	0.04445	-0.04356	-0.3404
cecal oligosacc	-0.6017	-0.8875	0.4588	-0.7785	0.8196	-0.1201	-0.4181	-0.06091	-0.07863	0.8787	0.9881	0.9362	0.5583	-0.4502	-0.4971

	Anaeroplasma	Akkermansia	Lactobacillus	unclassified	Streptococcus	unclassified	Anaerosporobacter	Eubacterium	Dorea	Johnsonella	Lachnospiracea_incertae_sedis	Marvinbryantia	Anaerostipes	unclassified	Blautia	Clostridium_XIVa
cecal butyrate	-0.9127	0.4636	0.2197	0.7125	0.2197	0.7279	-0.4047	-0.9596	-0.8378	0.6622	-0.6487	0.04553	-0.3552	-0.8346	-0.907	-0.06936
cecal propionate	-0.8705	0.6856	0.07055	0.7493	0.438	0.518	-0.5851	-0.865	-0.8467	0.4524	-0.7806	0.313	-0.4294	-0.8961	-0.8896	-0.2802
cecal acetate	-0.08862	-0.8543	0.7445	-0.497	-0.6402	0.5375	0.8972	-0.185	0.03442	0.4889	0.7596	-0.8833	0.04338	0.2477	-0.006402	0.9493
cecal baa	-0.814	0.8852	0.07566	0.5627	0.7394	0.2016	-0.5982	-0.7015	-0.8752	0.1028	-0.7319	0.6028	-0.6534	-0.9571	-0.8606	-0.3506
cecal lactate	-0.8551	0.7997	0.07758	0.6542	0.6185	0.347	-0.6008	-0.788	-0.8796	0.2608	-0.7649	0.4827	-0.5672	-0.948	-0.8902	-0.323
cecal alanine	-0.6871	0.9439	-0.09369	0.5887	0.7935	0.04695	-0.7138	-0.5641	-0.7704	-0.03008	-0.798	0.7265	-0.5874	-0.8888	-0.7465	-0.5118
cecal lysine	-0.7196	0.8923	0.1399	0.3199	0.8899	-0.04175	-0.493	-0.529	-0.8406	-0.1676	-0.5759	0.7428	-0.8036	-0.9271	-0.7801	-0.3071
cecal glutamate	0.4529	0.0188	-0.8825	0.6457	-0.4242	0.0122	-0.6093	0.2463	0.5486	0.2017	-0.5189	-0.05154	0.8567	0.4355	0.4528	-0.682
cecal tyrosine	0.1283	0.5419	-0.8942	0.8332	0.08984	-0.0888	-0.9359	0.02992	0.1475	0.04399	-0.8652	0.4133	0.4565	-0.03112	0.08896	-0.9312
cecal phenylalanine	-0.3871	0.9887	-0.4212	0.6207	0.7823	-0.2099	-0.8791	-0.2715	-0.4925	-0.2316	-0.8685	0.8599	-0.3693	-0.6675	-0.462	-0.7749
cecal glucose	0.8613	-0.6811	-0.4694	-0.1528	-0.7844	-0.1591	0.1958	0.6768	0.9537	0.007459	0.3453	-0.5027	0.917	0.9664	0.8983	-0.03645
cecal oligosacc	-0.7238	-0.1205	0.9734	-0.3398	0.1685	0.8741	0.5783	-0.6298	-0.7185	0.2962	0.3668	-0.251	-0.7373	-0.5631	-0.6929	0.7843

	Clostridium_XIVb	Flavonifractor	Hydrogenoanaerobacterium	Oscillibacter	Pseudoflavonifractor	Ruminococcus	Sporobacter	unclassified	Anaerotruncus	Butyricicoccus	Clostridium_IV	unclassified	unclassified	Allobaculum	Turicibacter
cecal butyrate	-0.2078	0.3119	0.3074	-0.0861	0.2434	0.3046	0.07595	0.2359	-0.9183	0.1346	-0.8949	0.05953	0.15	0.5108	0.2906
cecal propionate	-0.2883	0.4785	0.1261	-0.3006	0.03394	0.5462	0.01677	0.3188	-0.9776	0.3324	-0.8846	0.3242	0.3432	0.7241	0.02097
cecal acetate	0.01923	-0.4193	0.8016	0.9579	0.8835	-0.8451	0.5117	-0.03738	0.464	-0.5025	0.00861	-0.8833	-0.4849	-0.8613	0.8803
cecal baa	-0.5449	0.7578	0.0655	-0.3777	-0.06667	0.7919	0.1604	0.5722	-0.892	0.6548	-0.8686	0.614	0.6622	0.8717	-0.2777
cecal lactate	-0.4423	0.6479	0.09641	-0.3476	-0.02039	0.6959	0.102	0.4714	-0.9455	0.5236	-0.8926	0.495	0.5326	0.8204	-0.1482
cecal alanine	-0.4892	0.7613	-0.1175	-0.5375	-0.2518	0.8809	0.03579	0.5166	-0.8723	0.6913	-0.7573	0.7361	0.6938	0.9497	-0.4431
cecal lysine	-0.7327	0.917	0.07781	-0.3376	-0.07374	0.8824	0.3202	0.7538	-0.7189	0.8512	-0.7977	0.7514	0.8566	0.8697	-0.4448
cecal glutamate	0.8939	-0.6294	-0.7884	-0.6598	-0.7084	-0.09992	-0.9936	-0.8827	-0.1807	-0.575	0.4709	-0.05326	-0.591	0.09633	-0.1233
cecal tyrosine	0.527	-0.1322	-0.8404	-0.9248	-0.8434	0.4228	-0.8852	-0.5042	-0.5605	-0.09659	0.09617	0.4154	-0.1121	0.607	-0.4482
cecal phenylalanine	-0.2943	0.6563	-0.459	-0.7945	-0.5793	0.9403	-0.2375	0.3204	-0.7732	0.6503	-0.475	0.866	0.6435	0.998	-0.6821
cecal glucose	0.8471	-0.8945	-0.4184	-0.00457	-0.2761	-0.6768	-0.5871	-0.8637	0.6304	-0.7865	0.9129	-0.5124	-0.7998	-0.6507	0.1616
cecal oligosacc	-0.7119	0.4281	0.9621	0.7647	0.9077	-0.09789	0.9129	0.7091	-0.08524	0.2885	-0.6956	-0.2441	0.3123	-0.1586	0.5109

	unclassified	unclassified	TM7_genus_incertae_sedis	unclassified	Barnesiella	unclassified	Odoribacter	Parabacteroides	unclassified	Alistipes	Bacteroides	unclassified	unclassified
cecal butyrate	-0.9861	-0.862	0.1062	-0.4008	0.2803	0.9537	-0.02095	-0.6234	-0.2003	-0.1454	-0.3826	0.5598	0.9318
cecal propionate	-0.9496	-0.7031	0.1065	-0.4587	0.4775	0.8592	-0.209	-0.4031	-0.3237	-0.2571	-0.1412	0.7608	0.8489
cecal acetate	0.02659	-0.3064	0.3047	0.5754	-0.935	0.3955	0.8945	-0.7279	0.7527	0.7128	-0.8856	-0.7786	-0.01275
cecal baa	-0.8384	-0.406	0.3102	-0.2819	0.5177	0.7026	-0.2391	-0.1747	-0.2398	-0.1595	0.05893	0.9237	0.5929
cecal lactate	-0.9037	-0.546	0.2275	-0.3633	0.5067	0.7861	-0.2272	-0.2815	-0.2788	-0.203	-0.03141	0.867	0.7179
cecal alanine	-0.7308	-0.2752	0.2131	-0.3615	0.653	0.5631	-0.4013	0.01368	-0.3667	-0.2877	0.2497	0.9779	0.5087
cecal lysine	-0.6845	-0.1461	0.495	-0.05885	0.4383	0.537	-0.1723	-0.02658	-0.08536	-0.001735	0.1535	0.9389	0.3298
cecal glutamate	0.2137	-0.1027	-0.9916	-0.8774	0.636	-0.2745	-0.7802	0.3853	-0.8852	-0.9203	0.4861	-0.06148	0.2504
cecal tyrosine	-0.1021	-0.1157	-0.7746	-0.9512	0.943	-0.05493	-0.9571	0.4671	-0.9974	-0.9879	0.6668	0.4743	0.3862
cecal phenylalanine	-0.4742	-0.0428	-0.03095	-0.5173	0.8576	0.2653	-0.6825	0.3466	-0.6002	-0.5327	0.5719	0.9741	0.3398
cecal glucose	0.7706	0.2801	-0.7053	-0.1792	-0.1188	-0.6907	-0.1707	0.3208	-0.2176	-0.2979	0.1804	-0.7652	-0.3608
cecal oligosacc	-0.5441	-0.3846	0.8521	0.6583	-0.6608	0.651	0.8547	-0.7795	0.8069	0.8404	-0.8075	0.001888	0.1972

Ahr -/+
Genus v Metabolite Pearson correlation p

	Mucispirillum	unclassified	Enterorhabdus	unclassified	Parasutterella	unclassified	unclassified	unclassified	Vampirovibrio	unclassified	Bilophila	unclassified	unclassified	Helicobacter	unclassified
cecal butyrate	0.8724	0.9974	0.058	0.1301	0.358	0.1988	0.727	0.1167	0.7157	0.9897	0.7308	0.4907	0.5795	0.4719	0.8002
cecal propionate	0.86	0.8295	0.1816	0.1731	0.3401	0.2516	0.8546	0.0953	0.4735	0.9347	0.8549	0.5245	0.8405	0.7188	0.5513
cecal acetate	0.031	0.157	0.794	0.8719	0.9499	0.8183	0.8978	0.5992	0.1682	0.4835	0.3394	0.7063	0.1719	0.2943	0.0103
cecal baa	0.6221	0.8321	0.4268	0.2073	0.2318	0.5224	0.8017	0.265	0.2177	0.907	0.8214	0.4296	0.8585	0.9487	0.3794
cecal lactate	0.7232	0.8308	0.3067	0.1763	0.2644	0.3943	0.9498	0.1773	0.3183	0.9731	0.8303	0.4593	0.9915	0.9031	0.4467
cecal alanine	0.4531	0.6731	0.5556	0.3389	0.3375	0.5566	0.791	0.2813	0.1299	0.9768	0.9849	0.5625	0.6866	0.79	0.2403
cecal lysine	0.5222	0.9185	0.6608	0.2774	0.1822	0.8063	0.5223	0.496	0.1166	0.7295	0.7463	0.3578	0.676	0.7222	0.3549
cecal glutamate	0.6984	0.159	0.995	0.4647	0.2376	0.4591	0.2037	0.6024	0.8678	0.0073	0.0841	0.116	0.859	0.9865	0.6679
cecal tyrosine	0.3535	0.0328	0.8866	0.7883	0.6255	0.3706	0.4663	0.337	0.6096	0.0931	0.137	0.3948	0.5961	0.7826	0.2474
cecal phenylalanine	0.2059	0.3864	0.807	0.6499	0.6213	0.617	0.8454	0.3468	0.0736	0.7225	0.6851	0.8786	0.4238	0.55	0.059
cecal glucose	0.8402	0.7445	0.5367	0.1177	0.0337	0.8658	0.4762	0.5947	0.3161	0.4674	0.4332	0.1353	0.9555	0.9564	0.6596
cecal oligosacc	0.3983	0.1125	0.5412	0.2215	0.1804	0.8799	0.5819	0.9391	0.9214	0.1213	0.0119	0.0638	0.4417	0.5498	0.5029

	Anaeroplasma	Akkermansia	Lactobacillus	unclassified	Streptococcus	unclassified	Anaerosporobacter	Eubacterium	Dorea	Johnsonella	Lachnospiracea_incertae_sedis	Marvinbryantia	Anaerostipes	unclassified	Blautia	Clostridium_XIVa
cecal butyrate	0.0873	0.5364	0.7803	0.2875	0.7803	0.2721	0.5953	0.0404	0.1622	0.3378	0.3513	0.9545	0.6448	0.1654	0.093	0.9306
cecal propionate	0.1295	0.3144	0.9295	0.2507	0.562	0.482	0.4149	0.135	0.1533	0.5476	0.2194	0.689	0.5706	0.1039	0.1104	0.7198
cecal acetate	0.9114	0.1457	0.2555	0.503	0.3598	0.4625	0.1023	0.815	0.9656	0.5111	0.2404	0.1167	0.9566	0.7523	0.9936	0.0507
cecal baa	0.186	0.1348	0.9243	0.4373	0.2606	0.7984	0.4018	0.2985	0.1248	0.8972	0.2681	0.3971	0.3466	0.0429	0.1394	0.6494
cecal lactate	0.1449	0.2003	0.9224	0.3458	0.3815	0.653	0.3992	0.212	0.1204	0.7392	0.2351	0.5173	0.4328	0.052	0.1098	0.677
cecal alanine	0.3129	0.0561	0.9063	0.4113	0.2065	0.9531	0.2862	0.4359	0.2296	0.9699	0.202	0.2735	0.4126	0.1112	0.2535	0.4882
cecal lysine	0.2804	0.1077	0.8601	0.6802	0.1001	0.9583	0.507	0.471	0.1594	0.8324	0.4241	0.2572	0.1964	0.0729	0.2199	0.6929
cecal glutamate	0.5471	0.9812	0.1175	0.3543	0.5758	0.9878	0.3907	0.7537	0.4514	0.7983	0.4811	0.9485	0.1433	0.5645	0.5472	0.318
cecal tyrosine	0.8717	0.4581	0.1058	0.1668	0.9102	0.9112	0.0641	0.9701	0.8525	0.956	0.1348	0.5867	0.5435	0.9689	0.911	0.0688
cecal phenylalanine	0.6129	0.0113	0.5788	0.3793	0.2177	0.7901	0.1209	0.7285	0.5075	0.7684	0.1315	0.1401	0.6307	0.3325	0.538	0.2251
cecal glucose	0.1387	0.3189	0.5306	0.8472	0.2156	0.8409	0.8042	0.3232	0.0463	0.9925	0.6547	0.4973	0.083	0.0336	0.1017	0.9635
cecal oligosacc	0.2762	0.8795	0.0266	0.6602	0.8315	0.5258	0.4217	0.3702	0.2815	0.7038	0.6332	0.749	0.2627	0.4369	0.3071	0.2157

	Clostridium_XIVb	Flavonifractor	Hydrogenoanaerobacterium	Oscillibacter	Pseudoflavonifractor	Ruminococcus	Sporobacter	unclassified	Anaerotruncus	Butyricicoccus	Clostridium_IV	unclassified	unclassified	Allobaculum	Turicibacter
cecal butyrate	0.7922	0.6881	0.6926	0.9139	0.7566	0.6954	0.9241	0.7641	0.0817	0.8654	0.1051	0.9405	0.85	0.4892	0.7094
cecal propionate	0.7117	0.5215	0.8739	0.6994	0.9661	0.4538	0.9832	0.6812	0.0224	0.6676	0.1154	0.6758	0.6568	0.2759	0.979
cecal acetate	0.9808	0.5807	0.1984	0.0421	0.1165	0.1549	0.4883	0.9626	0.536	0.4975	0.9914	0.1167	0.5151	0.1387	0.1197
cecal baa	0.4551	0.2422	0.9345	0.6223	0.9333	0.2081	0.8396	0.4278	0.108	0.3452	0.1314	0.386	0.3378	0.1283	0.7223
cecal lactate	0.5577	0.3521	0.9036	0.6524	0.9796	0.3041	0.898	0.5286	0.0545	0.4764	0.1074	0.505	0.4674	0.1796	0.8518
cecal alanine	0.5108	0.2387	0.8825	0.4625	0.7482	0.1191	0.9642	0.4834	0.1277	0.3087	0.2427	0.2639	0.3062	0.0503	0.5569
cecal lysine	0.2673	0.083	0.9222	0.6624	0.9263	0.1176	0.6798	0.2462	0.2811	0.1488	0.2023	0.2486	0.1434	0.1303	0.5552
cecal glutamate	0.1061	0.3706	0.2116	0.3402	0.2916	0.9001	0.0064	0.1173	0.8193	0.425	0.5291	0.9467	0.409	0.9037	0.8767
cecal tyrosine	0.473	0.8678	0.1596	0.0752	0.1566	0.5771	0.1148	0.4958	0.4395	0.9034	0.9038	0.5846	0.8879	0.393	0.5518
cecal phenylalanine	0.7057	0.3437	0.541	0.2055	0.4207	0.0597	0.7625	0.6796	0.2268	0.3497	0.525	0.134	0.3565	0.002	0.3179
cecal glucose	0.1529	0.1055	0.5816	0.9954	0.7239	0.3232	0.4129	0.1363	0.3696	0.2135	0.0871	0.4876	0.2002	0.3493	0.8384
cecal oligosacc	0.2881	0.5719	0.0379	0.2353	0.0923	0.9021	0.0871	0.2909	0.9148	0.7115	0.3044	0.7559	0.6879	0.8414	0.4891

	unclassified	unclassified	TM7_genus_incertae_sedis	unclassified	Barnesiella	unclassified	Odoribacter	Parabacteroides	unclassified	Alistipes	Bacteroides	unclassified	unclassified
cecal butyrate	0.0139	0.138	0.8938	0.5992	0.7197	0.0463	0.9791	0.3766	0.7997	0.8546	0.6174	0.4402	0.0682
cecal propionate	0.0504	0.2969	0.8935	0.5413	0.5225	0.1408	0.791	0.5969	0.6763	0.7429	0.8588	0.2392	0.1512
cecal acetate	0.9734	0.6936	0.6953	0.4246	0.065	0.8045	0.1055	0.2721	0.2473	0.2872	0.1144	0.2214	0.9873
cecal baa	0.1616	0.594	0.6898	0.7181	0.4823	0.2974	0.7609	0.8253	0.7602	0.8405	0.9411	0.0763	0.4071
cecal lactate	0.0963	0.454	0.7725	0.6367	0.4933	0.2139	0.7728	0.7185	0.7212	0.797	0.9686	0.133	0.2827
cecal alanine	0.2692	0.7248	0.7869	0.6385	0.347	0.4369	0.5987	0.9863	0.6333	0.7123	0.7503	0.0221	0.4919
cecal lysine	0.3155	0.8539	0.505	0.9412	0.5617	0.463	0.8277	0.9734	0.9146	0.9983	0.8465	0.0611	0.6707
cecal glutamate	0.7863	0.8973	0.0084	0.1226	0.364	0.7255	0.2198	0.6147	0.1148	0.0797	0.5139	0.9385	0.7496
cecal tyrosine	0.8979	0.8843	0.2254	0.0488	0.057	0.9451	0.0429	0.5329	0.0026	0.0121	0.3332	0.5257	0.6138
cecal phenylalanine	0.5258	0.9572	0.969	0.4827	0.1424	0.7347	0.3175	0.6534	0.3998	0.4673	0.4281	0.0259	0.6602
cecal glucose	0.2294	0.7197	0.2947	0.8208	0.8812	0.3093	0.8293	0.6792	0.7824	0.7021	0.8196	0.2348	0.6392
cecal oligosacc	0.4559	0.6154	0.1479	0.3417	0.3392	0.349	0.1453	0.2205	0.1931	0.1596	0.1925	0.9981	0.8023

Ahr -/-
Genus v Metabolite Pearson correlation r

	Mucispirillum	unclassified	Enterorhabdus	unclassified	Parasutterella	unclassified	unclassified	unclassified	Vampirovibrio	unclassified	Bilophila	unclassified	unclassified	Helicobacter	unclassified
cecal butyrate	-0.7403	-0.2786	0.3134	0.7086	-0.4974	0.0439	0.7705	0.662	0.275	-0.6421	-0.734	-0.3627	-0.2796	-0.5646	-0.4586
cecal propionate	0.8501	0.5645	-0.3897	-0.4577	0.6966	-0.04112	-0.8957	-0.434	-0.4802	0.7723	0.5042	0.09405	0.5695	0.6496	0.349
cecal acetate	0.5602	0.4831	0.03918	-0.2495	0.8831	0.4068	-0.9824	-0.5242	-0.7677	0.459	0.2515	-0.2024	0.6104	0.9214	0.6573
cecal baa	0.1826	0.4619	0.4	0.2116	0.947	0.7331	-0.8646	-0.2946	-0.9711	0.1028	-0.2393	-0.629	0.6757	0.9773	0.6895
cecal lactate	0.7258	0.973	-0.657	0.4178	0.5326	-0.3838	-0.4554	0.6151	-0.4181	0.7857	-0.3124	-0.4664	0.8804	0.1358	-0.4933
cecal alanine	0.2855	0.776	0.126	0.584	0.9238	0.4641	-0.7373	0.227	-0.9548	0.2732	-0.5656	-0.8668	0.9165	0.7436	0.2297
cecal lysine	-0.1823	0.3641	0.6003	0.6007	0.7821	0.8266	-0.5497	0.02152	-0.9237	-0.2189	-0.6441	-0.8793	0.598	0.7797	0.5176
cecal glutamate	-0.2412	-0.4968	-0.347	-0.1837	-0.9615	-0.6932	0.8928	0.2965	0.9691	-0.1612	0.2054	0.6072	-0.7004	-0.9797	-0.6727
cecal tyrosine	0.5151	0.2682	0.06163	-0.4871	0.7267	0.3732	-0.9008	-0.7221	-0.5927	0.396	0.4761	0.04431	0.3842	0.853	0.7392
cecal phenylalanine	0.3202	0.006147	0.2273	-0.5967	0.5705	0.4612	-0.765	-0.8749	-0.4668	0.1872	0.5586	0.1748	0.1426	0.7993	0.8545
cecal glucose	0.734	0.09893	-0.773	-0.7896	-0.1715	-0.722	-0.1508	-0.2889	0.4352	0.7143	0.8591	0.7779	-0.09397	-0.2114	-0.2105
cecal oligosacc	0.5201	-0.09515	-0.7707	-0.714	-0.4945	-0.8553	0.2046	-0.1217	0.7152	0.5342	0.7802	0.8617	-0.3304	-0.5376	-0.4281

	Anaeroplasma	Akkermansia	Lactobacillus	unclassified	Streptococcus	unclassified	Anaerosporobacter	Eubacterium	Dorea	Johnsonella	Lachnospiracea_incertae_sedis	Marvinbryantia	Anaerostipes	unclassified	Blautia	Clostridium_XIVa
cecal butyrate	0.8488	0.3548	-0.9285	0.3479	-0.4593	-0.02079	-0.2624	-0.187	0.468	-0.1514	-0.5651	-0.8373	0.5896	-0.5016	0.6604	0.7171
cecal propionate	-0.9663	-0.0385	0.7661	-0.6235	0.614	-0.2644	0.006447	-0.1338	-0.3261	-0.008022	0.4343	0.7442	-0.7436	0.2296	-0.4572	-0.49
cecal acetate	-0.8138	0.02859	0.5937	-0.6886	0.8122	-0.4993	-0.3166	-0.2407	0.101	0.3398	0.6897	0.4023	-0.9402	-0.03377	-0.5918	-0.2189
cecal baa	-0.5028	0.3127	0.1287	-0.7444	0.996	-0.7877	-0.7264	-0.5042	0.5974	0.4706	0.6449	-0.1248	-0.9649	-0.4801	-0.4038	0.2786
cecal lactate	-0.6535	0.8051	-0.1411	-0.8317	0.3232	-0.6385	-0.4267	-0.8191	-0.04212	-0.7377	-0.4885	0.1897	-0.4092	-0.4759	0.5797	0.2918
cecal alanine	-0.4961	0.7513	-0.2238	-0.9331	0.8783	-0.9859	-0.9032	-0.8746	0.6305	0.01463	0.1624	-0.2602	-0.8543	-0.7831	0.1156	0.5848
cecal lysine	-0.1125	0.5288	-0.341	-0.6379	0.8552	-0.8764	-0.9342	-0.652	0.8983	0.4364	0.413	-0.5711	-0.7536	-0.7881	-0.09927	0.6784
cecal glutamate	0.554	-0.3139	-0.1669	0.7686	-0.9992	0.7846	0.7052	0.5084	-0.5526	-0.4362	-0.6348	0.07091	0.9789	0.4563	0.4034	-0.2435
cecal tyrosine	-0.7568	-0.2407	0.765	-0.4743	0.7549	-0.2544	-0.0815	0.02956	-0.0562	0.4491	0.7955	0.5265	-0.8224	0.2179	-0.7667	-0.4419
cecal phenylalanine	-0.5751	-0.4542	0.796	-0.2441	0.6525	-0.07516	0.04061	-0.2505	-0.05328	0.6329	0.9099	0.4759	-0.6976	0.3478	-0.9084	-0.5197
cecal glucose	-0.565	-0.4417	0.7864	0.09248	-0.2924	0.5332	0.7663	0.429	-0.9611	-0.3866	-0.06728	0.9712	0.3201	0.7919	-0.2053	-0.8787
cecal oligosacc	-0.2639	-0.473	0.5831	0.355	-0.6098	0.725	0.889	0.5349	-0.9965	-0.4869	-0.2961	0.8267	0.4598	0.8169	-0.01336	-0.8106

	Clostridium_XIVb	Flavonifractor	Hydrogenoanaerobacterium	Oscillibacter	Pseudoflavonifractor	Ruminococcus	Sporobacter	unclassified	Anaerotruncus	Butyricicoccus	Clostridium_IV	unclassified	unclassified	Allobaculum	Turicibacter
cecal butyrate	0.2049	-0.5434	-0.8602	0.9981	0.2125	-0.5947	0.03455	-0.9464	-0.7925	0.394	-0.9208	0.7221	-0.8506	-0.3721	0.07676
cecal propionate	-0.4429	0.3864	0.9413	-0.9273	-0.4433	0.4784	-0.3506	0.8305	0.5827	-0.1873	0.7735	-0.7846	0.7967	0.5937	0.1189
cecal acetate	-0.1849	0.6301	0.7059	-0.81	-0.1716	0.05915	-0.4091	0.5962	0.5999	0.1969	0.5439	-0.4331	0.4574	0.3363	0.5022
cecal baa	-0.04302	0.5723	0.3185	-0.4007	-0.01473	-0.459	-0.5709	0.1021	0.2879	0.6681	0.04758	0.001577	-0.05556	0.1379	0.8734
cecal lactate	-0.9684	-0.5608	0.6189	-0.09717	-0.9589	0.1373	-0.892	0.05281	-0.4715	0.2015	-0.0492	-0.6344	0.3063	0.9524	0.2353
cecal alanine	-0.4341	0.07232	0.3176	-0.1029	-0.4029	-0.503	-0.9024	-0.1606	-0.2028	0.7849	-0.2482	-0.08877	-0.1494	0.4599	0.8862
cecal lysine	0.05701	0.3449	-0.0957	0.07808	0.09224	-0.812	-0.6102	-0.379	-0.09629	0.9381	-0.4257	0.3752	-0.5018	-0.03997	0.9988
cecal glutamate	0.08751	-0.5612	-0.375	0.4425	0.06021	0.4087	0.5868	-0.1492	-0.3019	-0.6338	-0.09143	0.05868	-0.001024	-0.1873	-0.8499
cecal tyrosine	-0.01749	0.757	0.6841	-0.9064	-0.01159	0.1994	-0.153	0.735	0.7921	-0.007059	0.7067	-0.4296	0.5505	0.1878	0.3173
cecal phenylalanine	0.2362	0.8915	0.5143	-0.8706	0.2392	0.1689	0.08888	0.7159	0.9071	-0.06905	0.7151	-0.2616	0.4683	-0.06398	0.2419
cecal glucose	-0.3669	-0.04454	0.7199	-0.682	-0.396	0.9932	0.2489	0.8658	0.4533	-0.8955	0.8639	-0.8478	0.9519	0.4523	-0.7418
cecal oligosacc	-0.2776	-0.2506	0.4577	-0.3968	-0.3117	0.9701	0.4154	0.6568	0.2561	-0.9706	0.6759	-0.6809	0.785	0.3105	-0.9245

	unclassified	unclassified	TM7_genus_incertae_sedis	unclassified	Barnesiella	unclassified	Odoribacter	Parabacteroides	unclassified	Alistipes	Bacteroides	unclassified	unclassified
cecal butyrate	-0.8518	-0.8609	0.1419	0.5085	-0.1061	0.5539	-0.1487	0.9985	0.8383	-0.3398	0.2035	0.783	-0.7198
cecal propionate	0.9593	0.6543	-0.2009	-0.2654	0.3304	-0.2941	0.3629	-0.9293	-0.9662	0.09962	0.1025	-0.9377	0.4938
cecal acetate	0.7742	0.5182	-0.6193	0.05626	0.6462	-0.0177	0.05865	-0.8032	-0.8433	-0.2468	0.02185	-0.8278	0.2227
cecal baa	0.4353	0.08031	-0.8929	0.5333	0.9277	0.4483	-0.1088	-0.3881	-0.5602	-0.6895	0.1536	-0.5829	-0.2761
cecal lactate	0.6521	-0.3177	0.1538	0.3392	0.4154	0.4179	0.9245	-0.1093	-0.6564	-0.344	0.9322	-0.7187	-0.2876
cecal alanine	0.4364	-0.3291	-0.6565	0.7666	0.965	0.7434	0.2986	-0.09761	-0.55	-0.8548	0.6303	-0.6155	-0.5815
cecal lysine	0.03691	-0.3593	-0.9058	0.8498	0.9638	0.7802	-0.1948	0.09213	-0.181	-0.9336	0.776	-0.2357	-0.6771
cecal glutamate	-0.4888	-0.1102	0.8657	-0.5025	-0.9184	-0.4207	0.06389	0.4307	0.6091	0.6624	-0.1723	0.6304	0.2408
cecal tyrosine	0.7276	0.7205	-0.5485	-0.1764	0.4487	-0.2621	-0.08796	-0.8989	-0.7757	-0.02032	-0.2246	-0.7345	0.4444
cecal phenylalanine	0.5473	0.7986	-0.5733	-0.2715	0.3248	-0.3762	-0.3275	-0.8602	-0.5923	0.07935	-0.4691	-0.5347	0.5213
cecal glucose	0.6229	0.7225	0.621	-0.8704	-0.5678	-0.8297	0.4268	-0.6952	-0.5083	0.8298	-0.1355	-0.4457	0.8802
cecal oligosacc	0.3358	0.5502	0.8345	-0.8999	-0.8053	-0.8353	0.3829	-0.4121	-0.1968	0.326	-0.1673	-0.138	0.8103

Ahr -/-
Genus v Metabolite Pearson correlation p

	Mucispirillum	unclassified	Enterorhabdus	unclassified	Parasutterella	unclassified	unclassified	unclassified	Vampirovibrio	unclassified	Bilophila	unclassified	unclassified	Helicobacter	unclassified
cecal butyrate	0.2597	0.7214	0.6866	0.2914	0.5026	0.9561	0.2295	0.338	0.725	0.3579	0.266	0.6373	0.7204	0.4354	0.5414
cecal propionate	0.1499	0.4355	0.6103	0.5423	0.3034	0.9589	0.1043	0.566	0.5198	0.2277	0.4958	0.9059	0.4305	0.3504	0.651
cecal acetate	0.4398	0.5169	0.9608	0.7505	0.1169	0.5932	0.0176	0.4758	0.2323	0.541	0.7485	0.7976	0.3896	0.0786	0.3427
cecal baa	0.8174	0.5381	0.6	0.7884	0.053	0.2669	0.1354	0.7054	0.0289	0.8972	0.7607	0.371	0.3243	0.0227	0.3105
cecal lactate	0.2742	0.027	0.343	0.5822	0.4674	0.6162	0.5446	0.3849	0.5819	0.2143	0.6876	0.5336	0.1196	0.8642	0.5067
cecal alanine	0.7145	0.224	0.874	0.416	0.0762	0.5359	0.2627	0.773	0.0452	0.7268	0.4344	0.1332	0.0835	0.2564	0.7703
cecal lysine	0.8177	0.6359	0.3997	0.3993	0.2179	0.1734	0.4503	0.9785	0.0763	0.7811	0.3559	0.1207	0.402	0.2203	0.4824
cecal glutamate	0.7588	0.5032	0.653	0.8163	0.0385	0.3068	0.1072	0.7035	0.0309	0.8388	0.7946	0.3928	0.2996	0.0203	0.3273
cecal tyrosine	0.4849	0.7318	0.9384	0.5129	0.2733	0.6268	0.0992	0.2779	0.4073	0.604	0.5239	0.9557	0.6158	0.147	0.2608
cecal phenylalanine	0.6798	0.9939	0.7727	0.4033	0.4295	0.5388	0.235	0.1251	0.5332	0.8128	0.4414	0.8252	0.8574	0.2007	0.1455
cecal glucose	0.266	0.9011	0.227	0.2104	0.8285	0.278	0.8492	0.7111	0.5648	0.2857	0.1409	0.2221	0.906	0.7886	0.7895
cecal oligosacc	0.4799	0.9049	0.2293	0.286	0.5055	0.1447	0.7954	0.8783	0.2848	0.4658	0.2198	0.1383	0.6696	0.4624	0.5719

	Anaeroplasma	Akkermansia	Lactobacillus	unclassified	Streptococcus	unclassified	Anaerosporobacter	Eubacterium	Dorea	Johnsonella	Lachnospiracea_incertae_sedis	Marvinbryantia	Anaerostipes	unclassified	Blautia	Clostridium_XIVa
cecal butyrate	0.1512	0.6452	0.0715	0.6521	0.5407	0.9792	0.7376	0.813	0.532	0.8486	0.4349	0.1627	0.4104	0.4984	0.3396	0.2829
cecal propionate	0.0337	0.9615	0.2339	0.3765	0.386	0.7356	0.9936	0.8662	0.6739	0.992	0.5657	0.2558	0.2564	0.7704	0.5428	0.51
cecal acetate	0.1862	0.9714	0.4063	0.3114	0.1178	0.5007	0.6834	0.7593	0.899	0.6602	0.3103	0.5977	0.0598	0.9662	0.4082	0.7811
cecal baa	0.4972	0.6873	0.8713	0.2556	0.004	0.2123	0.2736	0.4958	0.4026	0.5294	0.3555	0.8752	0.0351	0.5199	0.5962	0.7214
cecal lactate	0.3465	0.1949	0.8589	0.1683	0.6768	0.3615	0.5733	0.1809	0.9579	0.2623	0.5115	0.8103	0.5908	0.5241	0.4203	0.7082
cecal alanine	0.5039	0.2487	0.7762	0.0669	0.1217	0.0141	0.0968	0.1254	0.3695	0.9854	0.8376	0.7398	0.1457	0.2169	0.8844	0.4152
cecal lysine	0.8875	0.4712	0.659	0.3621	0.1448	0.1236	0.0658	0.348	0.1017	0.5636	0.587	0.4289	0.2464	0.2119	0.9007	0.3216
cecal glutamate	0.446	0.6861	0.8331	0.2314	0.0008	0.2154	0.2948	0.4916	0.4474	0.5638	0.3652	0.9291	0.0211	0.5437	0.5966	0.7565
cecal tyrosine	0.2432	0.7593	0.235	0.5257	0.2451	0.7456	0.9185	0.9704	0.9438	0.5509	0.2045	0.4735	0.1776	0.7821	0.2333	0.5581
cecal phenylalanine	0.4249	0.5458	0.204	0.7559	0.3475	0.9248	0.9594	0.7495	0.9467	0.3671	0.0901	0.5241	0.3024	0.6522	0.0916	0.4803
cecal glucose	0.435	0.5583	0.2136	0.9075	0.7076	0.4668	0.2337	0.571	0.0389	0.6134	0.9327	0.0288	0.8799	0.2081	0.7947	0.1213
cecal oligosacc	0.7361	0.527	0.4169	0.645	0.3902	0.275	0.111	0.4651	0.0035	0.5131	0.7039	0.1733	0.5402	0.1831	0.9866	0.1894

	Clostridium_XIVb	Flavonifractor	Hydrogenoanaerobacterium	Oscillibacter	Pseudoflavonifractor	Ruminococcus	Sporobacter	unclassified	Anaerotruncus	Butyricicoccus	Clostridium_IV	unclassified	unclassified	Allobaculum	Turicibacter
cecal butyrate	0.7955	0.4566	0.1398	0.0019	0.7875	0.4053	0.9654	0.0536	0.2075	0.606	0.0792	0.2779	0.1494	0.6279	0.9232
cecal propionate	0.5571	0.6136	0.0587	0.0727	0.5567	0.5216	0.6494	0.1695	0.4173	0.8127	0.2265	0.2154	0.2033	0.4063	0.8811
cecal acetate	0.8151	0.3699	0.2941	0.19	0.8284	0.9408	0.5909	0.4038	0.4001	0.8031	0.4561	0.5669	0.5426	0.6637	0.4978
cecal baa	0.957	0.4277	0.6815	0.5993	0.9853	0.541	0.4291	0.8979	0.7121	0.3319	0.9524	0.9984	0.9444	0.8621	0.1266
cecal lactate	0.0316	0.4392	0.3811	0.9028	0.0411	0.8627	0.108	0.9472	0.5285	0.7985	0.9508	0.3656	0.6937	0.0476	0.7647
cecal alanine	0.5659	0.9277	0.6824	0.8971	0.5971	0.497	0.0976	0.8394	0.7972	0.2151	0.7518	0.9112	0.8506	0.5401	0.1138
cecal lysine	0.943	0.6551	0.9043	0.9219	0.9078	0.188	0.3898	0.621	0.9037	0.0619	0.5743	0.6248	0.4982	0.96	0.0012
cecal glutamate	0.9125	0.4388	0.625	0.5575	0.9398	0.5913	0.4132	0.8508	0.6981	0.3662	0.9086	0.9413	0.999	0.8127	0.1501
cecal tyrosine	0.9825	0.243	0.3159	0.0936	0.9884	0.8006	0.847	0.265	0.2079	0.9929	0.2933	0.5704	0.4495	0.8122	0.6827
cecal phenylalanine	0.7638	0.1085	0.4857	0.1294	0.7608	0.8311	0.9111	0.2841	0.0929	0.9309	0.2849	0.7384	0.5317	0.936	0.7581
cecal glucose	0.6331	0.9555	0.2801	0.318	0.604	0.0068	0.7511	0.1342	0.5467	0.1045	0.1361	0.1522	0.0481	0.5477	0.2582
cecal oligosacc	0.7224	0.7494	0.5423	0.6032	0.6883	0.0299	0.5846	0.3432	0.7439	0.0294	0.3241	0.3191	0.215	0.6895	0.0755

	unclassified	unclassified	TM7_genus_incertae_sedis	unclassified	Barnesiella	unclassified	Odoribacter	Parabacteroides	unclassified	Alistipes	Bacteroides	unclassified	unclassified
cecal butyrate	0.1482	0.1391	0.8581	0.4915	0.8939	0.4461	0.8513	0.0015	0.1617	0.6602	0.7965	0.219	0.2802
cecal propionate	0.0407	0.3459	0.7991	0.7346	0.6696	0.7059	0.6371	0.0707	0.0338	0.9004	0.8975	0.0623	0.5062
cecal acetate	0.2258	0.4818	0.3807	0.9437	0.3538	0.9823	0.9414	0.1968	0.1567	0.7532	0.9782	0.1722	0.7779
cecal baa	0.5647	0.9197	0.1071	0.4667	0.0723	0.5517	0.8912	0.6119	0.4398	0.3105	0.8464	0.4171	0.7239
cecal lactate	0.3479	0.6823	0.8462	0.6608	0.5846	0.5821	0.0755	0.8907	0.3436	0.656	0.0678	0.2813	0.7124
cecal alanine	0.5636	0.6709	0.3435	0.2334	0.035	0.2566	0.7014	0.9024	0.45	0.1453	0.3697	0.3845	0.4185
cecal lysine	0.9631	0.6407	0.0942	0.1502	0.0362	0.2198	0.8052	0.9079	0.819	0.0664	0.724	0.7643	0.3229
cecal glutamate	0.5112	0.8898	0.1343	0.4975	0.0816	0.5793	0.9361	0.5693	0.3909	0.3376	0.8277	0.3696	0.7592
cecal tyrosine	0.2724	0.2795	0.4515	0.8236	0.5513	0.7379	0.912	0.1011	0.2243	0.9797	0.7754	0.2655	0.5556
cecal phenylalanine	0.4527	0.2014	0.4267	0.7285	0.6752	0.6238	0.6725	0.1398	0.4077	0.9207	0.5309	0.4653	0.4789
cecal glucose	0.3771	0.2775	0.379	0.1296	0.4322	0.1703	0.5732	0.3048	0.4917	0.1702	0.8645	0.5543	0.1198
cecal oligosacc	0.6642	0.4498	0.1655	0.1001	0.1947	0.1647	0.6171	0.5879	0.8032	0.074	0.8327	0.862	0.1891
